# Multi-omics analysis to uncover constitutive priming and dynamic metabolic reprogramming conferring white rust resistance in *Brassica juncea*

**DOI:** 10.3389/fpls.2026.1918745

**Published:** 2026-08-19

**Authors:** Prajjwal Rai, Lakshman Prasad, Malkhan Singh Gurjar, Kaushal Pratap Singh, Nitish Rattan Bhardwaj, Praveen Kumar, Naveen Singh, Vishal Singh Somvanshi, Mahender Singh Saharan

**Affiliations:** 1 Division of Plant Pathology, Indian Agricultural Research Institute (ICAR-IARI), New Delhi, India; 2 National Institute for Research on Commercial Agricultural (ICAR-NIRCA), Research Station, Dinhata, Cooch Behar, India; 3 Indian Institute of Rapeseed Mustard Research (ICAR-IIRMR), Bharatpur, India; 4 National Institute of Plant Genome Research, New Delhi, India; 5 Division of Genetics, ICAR-IARI, New Delhi, India; 6 Division of Nematology, ICAR-IARI, New Delhi, India

**Keywords:** biotic stress tolerance, *Brassica juncea*, defense priming, genotypic resistance, multi-omics profiling, ubiquitin-proteasome pathway

## Abstract

White rust, caused by *Albugo candida*, is one of the most devastating diseases of Indian mustard (*Brassica juncea*), causing yield losses of up to 90%. Durable resistance sources within cultivated Brassica germplasm remain limited. In this study, near-isogenic lines (NILs) of *B. juncea* cv. *Varuna* harbouring resistance from an East European source (Donskaja-IV, possessing a single CC-NB-LRR protein-coding R gene) was used to investigate the molecular basis of resistance through integrated transcriptomic and metabolomic analyses at 48 and 96 hours post-inoculation (hpi). Transcriptomic profiling revealed that the resistant Varuna_WRR line exhibited significantly higher unique transcript expression (18.76%) compared to the susceptible parent (8.41%) during the progression of infection. Principal component analysis showed clear separation between genotypes based on infection status, time, and genetic background. In the resistant line, upregulated genes were enriched in ethylene-activated signaling, protein phosphorylation, endoplasmic reticulum stress response, pectin biosynthesis, and hypersensitive response at 48 hpi, shifting toward programmed cell death, protein ubiquitination, abscisic acid metabolism, and starch biosynthesis at 96 hpi. Conversely, the susceptible line displayed broad downregulation of primary metabolic processes, indicating metabolic exhaustion. Metabolomic analysis demonstrated that the resistant genotype accumulated higher levels of defense-related amino acids (proline, glutamine, glutamic acid, serine, threonine, glycine), carbohydrates, organic acids, and polyamines, supporting enhanced nitrogen assimilation, energy reserves, membrane stability, and signaling. Together, these findings indicate that constitutive priming and dynamic activation of defense signaling, protein turnover, and osmoprotectant accumulation underpin the enhanced resistance in Varuna_WRR against *Albugo candida*. This integrated multi-omics approach provides valuable insights for breeding durable white rust resistance in *Brassica juncea*.

## 1 Introduction

The Brassicaceae family comprises approximately 338 genera and 3,709 species, exhibiting remarkable diversity and serving as an important source of edible oil, vegetables, mustard condiments, and fodder (Warwick et al., 2006). *Brassica juncea* (L.) Czern & Coss is an amphidiploid (AABB, 2n = 36) derived from the natural hybridization of *Brassica rapa* (AA, 2n = 20) and *Brassica nigra* (BB, 2n = 16). It is widely cultivated across the world for its edible oil (Singh et al., 2021a). The total area, production, and yield of rapeseed-mustard in the world were 42.28 Mha, 85.1 Mt, and 2010 kg/ha, respectively, during 2024-25. India contributed approximately 21.05% to global area (8.90 Mha) and ~13.54% to global production (~11.52 million tonnes) (USDA, 2025). The genetic uniformity among cultivated varieties of *B. juncea* makes them highly vulnerable to pathogen attacks. The major biotic diseases posing a serious threat to mustard production worldwide include: *Alternaria brassicae* (Berk.) Sacc., *A. brassicicola* (Schwein.) Wiltshire, *Sclerotinia sclerotiorum* (Lib.) de Bary, *Albugo candida* (Pers.) Kunze, *Plasmodiophora brassicae* Woronin, *Erysiphe polygoni* DC., *Leptosphaeria maculans* (Desmaz.) Ces. & De Not., and *Hyaloperonospora parasitica* (Pers.: Fr.) Fr (Williams and Saha, 1993; Singh et al., 2021b). These diseases cause substantial yield losses globally due to the lack of effective vertical and horizontal resistance in currently cultivated varieties. Among these diseases, white rust caused by *A. candida* is characterized by localized white to pale cream-colored pustules on the lower side of leaves and inflorescences, often accompanied by hypertrophied flowers (staghead). Staghead formation results in complete failure of seed set and can cause yield losses of up to 90%. Yield losses vary with disease severity and are commonly reported in the range of 17–34% on average, with maximum losses reaching 60–90% in *B. juncea* in India (Vignesh et al., 2011; Anupriya et al., 2020; Dev et al., 2026).

Most cultivars of Indian mustard exhibit high susceptibility to white rust disease (Li et al., 2008; Awasthi et al., 2012). However, some genotypes from the east European gene pool have demonstrated resistance against the pathogen (Panjabi-Massand et al., 2010; Awasthi et al., 2012; Arora et al., 2019). Several studies have demonstrated that resistance to white rust exhibits quantitative inheritance (Singh et al., 2026). A large number of resistance loci and candidate genes have been characterized in several cruciferous species, including *Arabidopsis thaliana* (Borhan et al., 2001, 2008; Cevik et al., 2019), *B. carinata* (Thukral and Singh, 1986), *B. juncea* (Panjabi-Massand et al., 2010; Singh et al., 2015), *B. napus* (Ferreira et al., 1995), *B. nigra* (Delwiche and Williams, 1974), *B. rapa* (Ebrahimi et al., 1976; Kole et al., 2002), and *Raphanus sativus* (Humaydan and Williams, 1976). The near-isogenic lines (NILs) were developed from the popular *B. juncea* varieties (Varuna, Rohini, Pusa Jaikisan, and Pusa Bold) with introgression of BjuA5.WRR.a1 (a single CC-NB-LRR protein-coding R gene) from east European genepool Brassica line (Donskaja-IV) has shown resistance to multiple isolates of *A. candida* collected from diverse locations of India (Arora et al., 2019).

Metabolic changes are crucial for plant immunity against biotrophic pathogens, involving coordinated shifts in primary and secondary metabolism to limit pathogen proliferation while supporting host survival. In *B. juncea*, infection by the obligate oomycete *A. candida*, the causal agent of white rust, induces significant alterations in the host metabolome. Biochemical analyses have shown that resistant genotypes exhibit elevated levels of defense-related metabolites, including total phenols, polyphenols, proline, and antioxidant compounds, which correlate positively with reduced disease severity through enhanced ROS scavenging and osmotic protection (Rai et al., 2024; Kaur et al., 2011). GC-MS-based untargeted metabolomics, following protocols such as those established by Schauer et al. (2005) for extraction and derivatization of polar metabolites, has proven effective in capturing these dynamic responses. Studies have identified increased accumulation of sugars, amino acids, organic acids, phenylpropanoids, and glucosinolate breakdown products in resistant interactions, contributing to cell wall reinforcement and antimicrobial activity (Al-Khayri et al., 2023). In contrast, susceptible genotypes often display pathogen-mediated manipulation of host primary metabolism, resulting in higher availability of soluble sugars and amino acids that support pathogen nutrition (Mishra et al., 2009).

Comparative metabolomic investigations across Brassica species further highlight the redirection of carbon flux toward specialized metabolite biosynthesis, including phenylpropanoid and flavonoids and lignification precursors, as a key resistance mechanism against oomycete invasion (Shaw et al., 2022; Wei et al., 2024). Time-resolved metabolite profiling has revealed rapid induction of osmoprotectants and secondary metabolites within 24–48 hours post-inoculation in resistant lines, followed by sustained accumulation that restricts pustule formation and sporulation (Hahlbrock et al., 2003; Serag et al., 2023). These findings are consistent with broader plant-pathogen metabolomics research demonstrating that successful defense involves tight regulation of central carbon metabolism, the shikimate pathway, and phenylpropanoid biosynthesis (Castro-Moretti et al., 2020). These metabolic insights provide a biochemical foundation for understanding quantitative resistance against white rust and offer promising targets for metabolic engineering and biomarker development in oilseed mustard breeding programs. In the present investigation, we used *B. juncea* cv. Varuna and *B. juncea* cv. Varuna_WRR to investigate transcriptomic and metabolomic changes upon challenging of white rust pathogen at different time intervals and identify key transcripts and metabolites that confer immunity to the plant.

## 2 Materials and methods

### 2.1 Plant materials

*B. juncea* var. Varuna (highly susceptible) and *B. juncea* var. Varuna_WRR (resistant NIL possessing *BjuWRR1* gene) were used as plant material in the present investigation. The seeds of both the plant varieties were procured from Centre for Genetic Manipulation of Crop Plants (CGMCP), Delhi University South Campus, Delhi. The seeds were surface sterilized with 70% ethyl alcohol for two minutes, followed by three times washing with sterilized double distilled water (DDW), then 4% sodium hypochlorite for ten minutes and finally rinsed with sterilized double distilled water three times. After soaking on sterile filter paper, the seeds were sown in earthen plastic pots of 24cm filled with a mixture of sterilized soil:vermicompost (3:1) in three replications under poly-house conditions. The plants were provided sterile water as and when required, and only a single plant was grown in each pot of replications. At two leaf stages, the plants were transferred to a growth chamber and maintained at 22 °C temperature along with 90% relative humidity and 16/8 hours light and dark cycles, respectively, until the artificial inoculation of the pathogen was done.

### 2.2 Artificial inoculation of the pathogen

**Figure 1:**
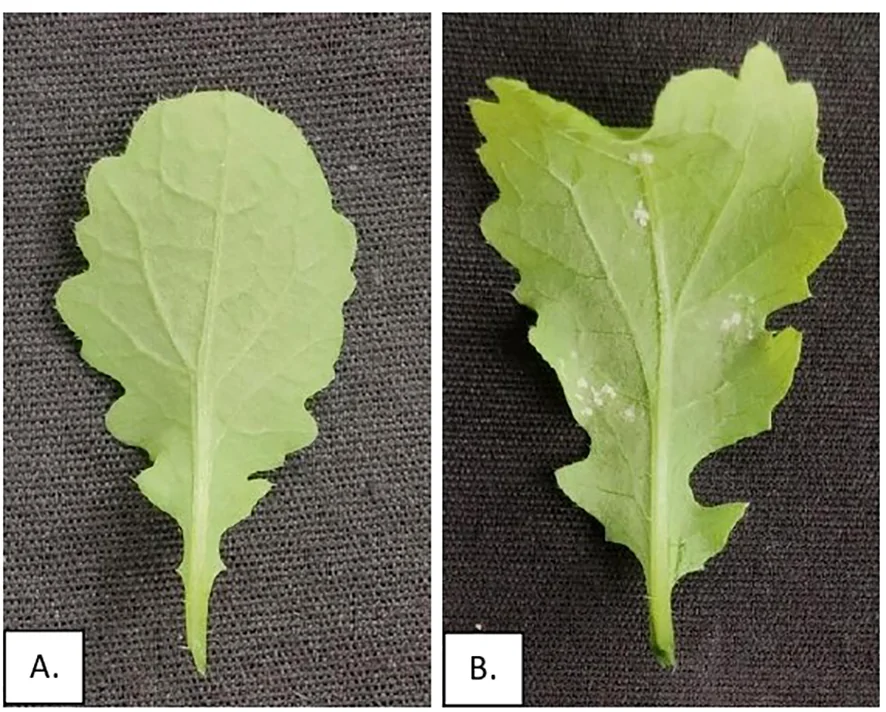
Disease development after artificial inoculation of *Albugo candida* to *B. juncea* var. VarunaWRR **(A)** and *B. juncea* var. Varuna **(B)** on 8^th^ day of inoculation.

For the pathogen inoculation, the zoosporangia of the white rust pathogen were collected from highly susceptible Brassica plant leaves of the early-sown susceptible lines growing in the agricultural farm of ICAR-Indian Agricultural Research Institute, New Delhi. The inoculum was prepared in sterilized DDW by dipping infected leaves for 2 h, after which the infected leaves were rubbed gently in water to harvest zoosporangia. The DDW containing zoosporangia of the white rust pathogen was kept at 8 °C for 4 hours to allow germination. The inoculum containing zoospores and zoosporangia of white rust (2 × 10^5^ spores/ml) was sprayed on the foliage of potted Brassica plants at the age of 21 days using a hand atomizer, while control plants were mock-sprayed with sterile water. The temperature was maintained at 20°C at 90% relative humidity and 12/12 hours light and dark cycles (**Figure 1**).

### 2.3 Leaf sampling, total RNA isolation, library preparation and sequencing

The total RNA was isolated from the fresh leaves of inoculated and uninoculated (mocked) plants of *B. juncea* cv. Varuna and *B. juncea* cv. Varuna_WRR at 48 and 96 hours post-inoculation (hpi) of the pathogen suspension in three biological replications. The leaf samples were collected in liquid nitrogen and stored further at (-)80°C until the RNA extraction experiment. The total RNA was isolated from the collected leaf samples of susceptible and resistant plants (pathogen-inoculated and mock-inoculated) using TRIzol reagent (Invitrogen, CA, United States) using standard instructions of the manufacturer. The RNA samples were treated with 1 mL of 2 U DNase I (Invitrogen, USA) by incubating at 37 °C for 30 minutes to remove DNA from the samples. After total RNA isolation, the samples were checked for quality and quantity using the NanoDrop 8000 (OD260/280) and Qubit3.0 fluorometer (Invitrogen Life Technologies, United States), respectively. The quality of the isolated RNA samples was also checked using a 1% agarose gel electrophoresis for RNA degradation and contamination. The total RNA samples from each set of experiments that passed for both quality and quantity were pooled for all the time points (48 and 96 hpi). The pooled total RNA of all three biological replicates from each set of experiments was outsourced to Biokart India Pvt. Ltd., Bangalore (India) for whole transcriptome sequencing using the Illumina HiSeq 2500 platform.

### 2.4 RNA-seq analysis

The raw data of the allohexaploid Brassica transcriptomesequence were submitted to the Sequence Read Archive (SRA) of the National Center for Biotechnology Information (NCBI) under accession number SRR14934389 and BioProject number PRJNA741791. Principal Component Analysis (PCA) was conducted to identify the overall relationships among samples. The raw RNA-seq reads generated after Illumina sequencing were checked for standard quality control, GC content, and removed the low quality sequences (Q<30) and adapters by trimming with FastQC v0.12.0 (https://github.com/s-andrews/FastQC) and Trim Galore tools (https://github.com/FelixKrueger/TrimGalore), respectively. Following trimming, reads were normalized using BBNorm to reduce biases introduced by highly abundant sequences and ensure uniform coverage across transcripts. The high-quality reads obtained after removing adapters and low-quality reads were mapped against the reference genome assembly of *A. candida* (GCA_905220665.1) using HISAT2 to check the potential similarity of reads and remove identical reads. The unmapped reads were extracted and again mapped against the *B. juncea* cv. Varuna (GCA_015484525) reference genome assembly using the HISAT2 tool (Kim et al., 2019).

### 2.5 *De-novo* transcriptome assembly and quantification

*De-novo* transcriptome assembly was performed using Trinity (Grabherr et al., 2011), a widely used assembler for reconstructing transcript sequences from short RNA-Seq reads. To remove redundant sequences, the assembled transcripts were processed using CD-HIT, which clusters similar sequences based on a sequence identity threshold of 0.9. For transcript quantification, Kallisto, a pseudo-alignment-based method, was employed to estimate transcript abundances efficiently. The assembled transcripts were annotated by performing a BLASTX search against the UniProt database. This step facilitated the functional characterization of transcripts by aligning them to known protein sequences.

### 2.6 Differential expression and functional enrichment analysis

Differentially expressed genes (DEGs) were identified using DESeq2, a statistical package designed for RNA-Seq differential expression analysis. The following criteria were used to determine significant DEGs:

• |Log2(fold_change)| > 2
• p-value < 0.05
• False discovery rate (FDR) < 0.1

To gain insights into the biological significance of DEGs, functional enrichment analysis was performed using the Database for Annotation, Visualization, and Integrated Discovery (DAVID). This analysis identified enriched Gene Ontology (GO) terms and KEGG pathways, aiding in the interpretation of molecular functions, biological processes, and cellular components associated with differentially expressed genes (Singh et al., 2022).

### 2.7 Metabolite dynamics of resistant and susceptible *B. juncea* genotypes

The modified Schauer et al. (2005) protocol was used for GC-MS-based metabolite analysis. A total of 20–25 mg of lyophilized leaf samples collected from 21-day-old *A. candida*-inoculated *B. juncea* plants were extracted separately at different time intervals. Extraction was performed using 480 µL of pure methanol, followed by the addition of 20 µL of 0.2 mg mL^-^¹ ribitol (adonitol) as an internal standard. Healthy (non-inoculated) plant samples served as controls. The mixture was vigorously shaken for 2 min and heated at 70 °C for 15 min. Thereafter, an equal volume of water was added, shaken vigorously, and 250 µL of chloroform was incorporated with thorough mixing. This mixture was centrifuged at 2200 g for 10 min at room temperature (~25 °C). The upper aqueous phase was collected and dried in a speed vacuum concentrator at 35 °C. The dried fraction was then re-dissolved in 40 µL of 20 mg mL^-^¹ methoxyamine hydrochloride in pyridine and incubated for 90 min at 37 °C. After that, 60 µL of MSTFA [N-methyl-N-(trimethylsilyl) trifluoroacetamide] was added and incubated for 30 min at 37 °C. Following derivatization, the sample was transferred to a GC-MS vial containing an insert. The injection volume was set at 0.2 µL in split mode with a split ratio of 5.

Gas chromatography–mass spectrometry (GC–MS) analysis for untargeted metabolite profiling was performed on a Shimadzu system consisting of a GC-2010 Plus gas chromatograph coupled with a TQ 8050 mass spectrometer and an AOC-20s autosampler–AOC-20i autoinjector. Separation was carried out on an SH-Rxi-5Sil MS capillary column (30 m × 0.25 mm × 0.25 µm) (Restek Corporation, USA) using helium as carrier gas at a flow rate of 1 mL min^-^¹. The oven temperature program started with 80 °C isothermal for 2 min, followed by a ramp of 5 °C min^-^¹ to 250 °C (hold 2 min), and a final ramp of 10 °C min^-^¹ with a hold time of 24 min. Total run time was 67 min with a solvent delay of 4.5 min. Chromatogram integration and mass spectral analysis were performed using Shimadzu LabSolutions software (GC-MS solution Version 4.53 SP1). Derivatized metabolites were identified using the NIST17 spectral library.

## 3 Results

### 3.1 Transcriptome sequencing and quality control

Paired-end RNA sequencing of 8 *Brassica juncea* samples (comprising *Albugo candida*-inoculated and mock-inoculated plants) generated high-quality raw reads on the Illumina platform. MultiQC analysis of FastQC reports revealed consistently high sequence quality across all samples. The mean per-base Phred quality scores remained above 35 throughout the 150 bp read length, with the majority of reads exhibiting Phred scores ≥34. The per-sequence quality score distribution showed a strong peak between Phred 37–40, indicating excellent base-calling accuracy. GC content across the samples ranged from 49% to 74%, consistent with the expected GC distribution for *Brassica* transcriptome data. Sequence duplication levels were typical for RNA-seq datasets, reflecting the inherent variation in transcript abundance. However, several samples exhibited moderate to high levels of overrepresented sequences (ranging from 5% to >20% of total reads in certain libraries), likely originating from highly expressed transcripts or residual adapters (adapters and poly A, G, and T sequences). Adapter contamination was detected in multiple samples, with adapter content increasing toward the 3′ end of reads and reaching up to 30% in a few libraries. Despite the presence of some overrepresented sequences and residual adapter content, the overall data quality was high, with no samples showing critical failures in per-base sequence quality or N-content. These results confirm that the raw sequencing data were of sufficient quality for downstream processing, including adapter trimming, quality filtering, and subsequent transcriptome analysis (**Table 1**).

**Table 1:** Summary of raw sequence data.

Sample	Unique reads	Duplicate reads	Total reads (R1+R2)	Total GC %	Gbp
Varuna-I48	10720301	17683191	28403492	62.25	4.29
Varuna-I96	9888272	16007926	25896198	61.8	3.91
Varuna- M48	10501923	11296751	21798674	51.85	3.29
Varuna- M96	11035284	10809512	21844796	49.5	3.30
Varuna_WRR- I48	8148332	13955080	22103412	63.15	3.34
Varuna_WRR-I96	9676654	11251374	20928028	53.75	3.16
Varuna_WRR-M48	5382950	15568704	20951654	74.35	3.16
Varuna_WRR-M96	8122037	12779071	20901108	61.15	3.16

### 3.2 Principal component analysis

**Figure 2:**
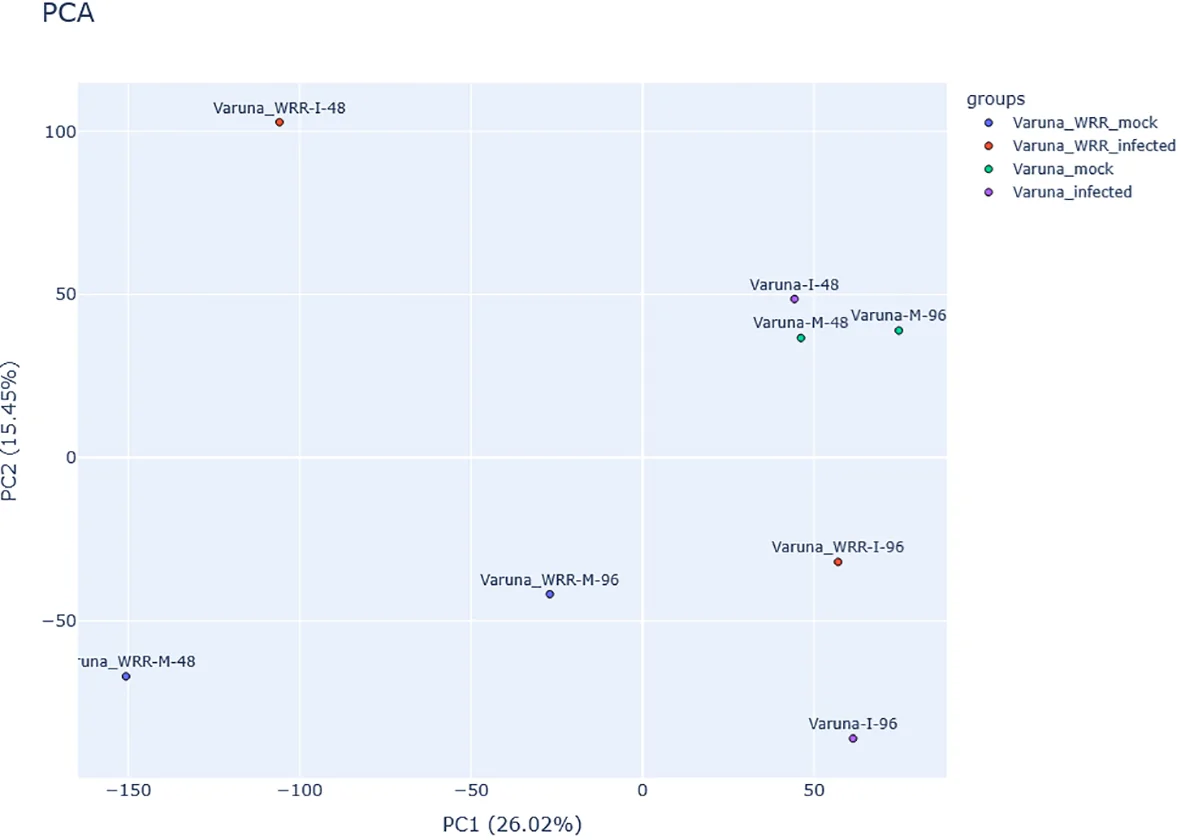
Principal component analysis (PCA) of transcriptome profiles in *B. juncea* cv. Varuna and *B. juncea* cv. Varuna_WRR varieties under mock and infection conditions on 48 and 96 hours time intervals.

PCA was performed to explore the overall variance and clustering patterns in the transcriptome data sets. The first two principal components (PC1 and PC 2) accounted for 26.02% and 15.45% of the total variance, respectively, cumulatively explaining 41.47% of the variation. As shown in **Figure 1**, a clear separation of samples was observed according to both infection status and time point. Mock-treated samples at 48 hours post-treatment, particularly Varuna_WRR-M-48, were distinctly positioned on the negative side of PC1. In contrast, infected samples, especially at 96 hours (Varuna_WRR-I-96 and Varuna-I-96), tended toward the positive PC1 and negative PC 2 quadrant. Samples at 48 hours generally exhibited higher PC2 scores compared to 96-hour samples, indicating a strong temporal effect on the molecular profile. Furthermore, the WRR genotype displayed noticeable separation from the standard Varuna background under mock conditions, suggesting inherent baseline differences between the resistant and susceptible lines. Overall, these results demonstrate that infection status, post-inoculation time, and genetic background are the major sources of variation in the data set, with progressive transcriptional/metabolic expression occurring during the infection process (**Figure 2**).

### 3.3 Differential gene expressions in *A. candida*-inoculated and mock plants

The DEGs analysis revealed a robust transcriptional response to mock and pathogen inoculation in the Varuna variety at both 48 and 96 hours post-inoculation. At 48 hours, 4,795 genes were differentially expressed (|Log2 fold_change| > 2, adjusted p-value < 0.05), including 2,180 up-regulated and 2,615 down-regulated genes (**Supplementary Figure 1A**). This response intensified at 96 hours, with a total of 5,788 differentially expressed genes comprising 2,541 up-regulated and 3,247 down-regulated genes (**Supplementary Figure 2A**). Volcano plots displayed clear asymmetry at both time points, characterized by a greater number of down-regulated genes and several highly significant changes (–log_10_(p.adj) > 100 at 48 h and > 300 at 96 h) (**Supplementary Figures 1B, 2B**). Heatmap clustering confirmed distinct separation between mock and infected samples, highlighting two major expression clusters representing strongly induced and repressed genes, respectively (**Supplementary Figures 1C, 2C**).

A comparative analysis in the resistant Varuna_WRR background showed distinct transcriptional dynamics between mock and pathogen-inoculated lines. At 48 hours post-inoculation, 6,391 genes were differentially expressed, followed by 5,177 genes at 96 hours. Notably, the response in Varuna_WRR exhibited a clear bias toward up-regulation, particularly at the early time point (approximately 4,502 DEGs at 48 h; **Supplementary Figure 3A**), with 3,471 up-regulated genes at 96 h (**Supplementary Figure 4A**). Volcano plots illustrated these contrasting patterns, revealing more pronounced up-regulation in the inoculated resistant line compared to its mock control (**Supplementary Figures 3B, 4B**). Heatmap analysis further confirmed distinct expression profiles between mock and infected samples, with Varuna_WRR displaying rapid and extensive induction of genes early after infection (**Supplementary Figures 3C, 4C**). These findings suggest that while both genotypes undergo substantial transcriptional expression upon infection, the resistant Varuna_WRR line mounts an earlier and more up-regulation-oriented defense response, which may contribute to its enhanced resistance.

Under mock conditions, 730 genes were differentially expressed between susceptible Varuna and resistant Varuna_WRR lines. Strikingly, nearly all (728 genes) were up-regulated in Varuna_WRR, with only two down-regulated genes detected (**Supplementary Figure 5A**). The volcano plot highlighted this strong bias, showing a prominent cluster of highly significant up-regulated genes with log_2_ fold change, values exceeding 15 (**Supplementary Figure 5B**). Heatmap clustering confirmed constitutively higher expression of these genes in the resistant line across both time points (**Supplementary Figures 5C, 6C**). Similarly, under infected conditions, 393 genes were differentially expressed between Varuna and Varuna_WRR, of which 289 were up-regulated and 104 down-regulated in the resistant genotype (**Supplementary Figure 6A**). The volcano plot and heatmap analyses consistently demonstrated enhanced expression of numerous genes in Varuna_WRR even during active infection (**Supplementary Figure 6B**). These results indicate that the resistant genotype possesses a constitutively activated transcriptional program and maintains a significantly different, defense-oriented profile compared to the susceptible line both before and during pathogen challenge.

### 3.4 Functional enrichment analysis (GO and KEGG pathways) of DEGs

#### 3.4.1 Functional enrichment in *B. juncea* var. varuna

To gain biological insights into the transcriptional expression during pathogen infection and mock inoculation, Gene Ontology (GO) and KEGG pathway enrichment analyses were performed separately on up- and down-regulated genes in the Varuna variety at 48 and 96 hours. The functional annotations through GO analysis annotated the DEGs regulated against the mock and *A. candida* inoculation into three different classes i.e., molecular function (MF), biological process (BP), and cellular component (CC). Down-regulated genes at both time points were significantly enriched in cellular maintenance and metabolic processes, including signal transduction (27 genes), methylation (19 genes), pectin biosynthetic process (4 genes), phosphatidylinositol-mediated signaling (4 genes), negative regulation of ethylene-activated signaling pathway (3 genes), very long-chain fatty acid catabolic process (2 genes), and long-chain fatty acid import into peroxisome (2 genes). Molecular functions related to transferase activities (43 genes), oxidoreductase activity (6 genes), and carbohydrate-active enzymes were overrepresented, while KEGG pathways highlighted the sulfur relay system, nucleotide metabolism, amino acid biosynthesis, and glycerolipid metabolism (**Supplementary Figures 7A, 8A**). This indicates a broad suppression of primary metabolism, cell wall biosynthesis, and certain signaling cascades during infection.

In contrast, up-regulated genes showed strong enrichment in defense and stress-adaptive processes at both time points. Notable biological processes included regulation of salicylic acid-mediated signaling pathway (4 genes), programmed cell death (3 genes), abscisic acid metabolic process (4 genes), glucose 6-phosphate metabolic process (3 genes), hexose metabolic process (3 genes), protein phosphorylation (46 genes), and chromatin organization (7 genes). Molecular functions were enriched in ATP binding (103 genes), protein kinase activity (42 genes), metal ion binding (61 genes), and various carbohydrate metabolism enzymes. KEGG pathway analysis further highlighted nucleocytoplasmic transport, biosynthesis of nucleotide sugars, amino sugar and nucleotide sugar metabolism, pentose and glucuronate interconversions, and glycolysis/gluconeogenesis (**Supplementary Figures 7B, 8B**). These enrichment patterns demonstrate that pathogen infection induces conserved transcriptional expression across both 48 and 96 hours, characterized by the down-regulation of primary metabolic and cell wall-related pathways and the coordinated activation of defense signaling (especially salicylic acid), programmed cell death, and carbohydrate metabolism, with the response becoming more pronounced at the later time point.

#### 3.4.2 Functional enrichment in *B. juncea* var. Varuna_WRR

The functional enrichment analysis of DEGs at 48 and 96 hours post-inoculation and mock plants revealed dynamic yet interconnected biological processes underlying the transcriptional response in the Varuna_WRR variety. At 48 hours, up-regulated genes were strongly enriched in mRNA destabilization (4 genes), mRNA transport (6 genes), pectin biosynthetic process (5 genes), response to endoplasmic reticulum stress (5 genes), ethylene-activated signaling pathway (12 genes), protein phosphorylation (56 genes), and pyruvate metabolic process (5 genes) (**Supplementary Figure 9A**). By 96 hours, the up-regulated response shifted toward glycophagy (3 genes), glucose 6-phosphate metabolic process (3 genes), starch biosynthetic process (4 genes), programmed cell death (3 genes), abscisic acid metabolic process (4 genes), plant-type hypersensitive response (5 genes), and protein ubiquitination (32 genes). Common molecular functions across both time points included kinase activity, ATP binding, and ubiquitin-related processes, while KEGG pathways highlighted carbohydrate metabolism, glycolysis/gluconeogenesis, and nucleotide sugar biosynthesis (**Supplementary Figure 9B**). Down-regulated genes at 48 hours were enriched in transmembrane transport (18 genes), fatty acid beta-oxidation (2 genes), cellulose catabolic process (4 genes), and various phosphatase activities (**Supplementary Figure 10A**). At 96 hours, down-regulation was prominent in protein import into chloroplast stroma (3 genes), tRNA thio-modification (2 genes), methylation (12 genes), and methyltransferase activities (8 genes). Across both time points, KEGG pathways related to metabolic processes, pyruvate metabolism, and biosynthesis of cofactors were consistently suppressed (**Supplementary Figure 10B**). These temporal patterns demonstrate progressive transcriptional expression during infection. The early phase (48 h) involves activation of ER stress and ethylene signaling with suppression of lipid and cell wall catabolism, while the later phase (96 h) sustains defense activation through hypersensitive response, programmed cell death, and carbohydrate remobilization, accompanied by reduced chloroplast protein import and tRNA modification. This indicates a coordinated shift from basal metabolism toward sustained immunity and stress adaptation in the Varuna_WRR variety.

#### 3.4.3 Functional enrichment in *B. juncea* var. Varuna and Varuna_WRR

The functional enrichment analysis was performed on DEGs between the susceptible Varuna and resistant Varuna_WRR lines under both mock and pathogen-inoculated conditions to elucidate constitutive and infection-induced transcriptional differences. Under mock conditions, genes constitutively upregulated in the resistant Varuna_WRR line showed significant enrichment in several biological processes, including cellulose catabolic process (3 genes) and protein import into the nucleus (3 genes). At the cellular component level, strong enrichment was observed for small ribosomal subunit (4 genes), chloroplast (10 genes), cytosol (20 genes), and endoplasmic reticulum (8 genes). Molecular function analysis highlighted key activities such as ribulose-bisphosphate carboxylase (2 genes), cellulose (3 genes), and sterol binding (3 genes), while KEGG pathway analysis pointed to nucleocytoplasmic transport (5 genes). These findings suggest that the resistant genotype maintains a primed cellular state with enhanced photosynthetic machinery, ribosomal biogenesis, and cell wall remodeling even in the absence of pathogen pressure (**Supplementary Figure 11A**). Under pathogen-inoculated conditions, the transcriptional differences became more defense-oriented. Genes upregulated in Varuna_WRR were significantly enriched in carotene catabolic process (2 genes), response to blue light (2 genes), RNA modification (4 genes), circadian rhythm (2 genes), and proteolysis (6 genes). Important molecular functions included oxidoreductase activity (incorporating molecular oxygen) (3 genes), carotenoid dioxygenase activity (2 genes), and photoreceptor activity (2 genes) (**Supplementary Figure 12A**). Conversely, genes downregulated in the resistant line (i.e., relatively higher in susceptible Varuna) were enriched in activation of GTPase activity (2 genes), protein localization to phagophore assembly site (2 genes), and notably nutrient reservoir activity (3 genes) (**Supplementary Figure 12B**).

Collectively, these comparative results demonstrate that the resistant Varuna_WRR genotype possesses a constitutively active transcriptional program supporting chloroplast function, protein synthesis, and cell wall dynamics under normal conditions. Upon pathogen challenge, this baseline advantage shifts toward activation of photoprotective carotenoid metabolism, light signaling, circadian regulation, and enhanced protein turnover, potentially enabling faster and more efficient defense deployment. In contrast, the susceptible line shows relatively higher expression of storage and autophagy-related components during infection. This dynamic expression likely contributes to the enhanced disease resistance observed in the Varuna_WRR line.

### 3.5 Analysis (GO and Uniprot pathways) for top ten functions from expressed genes in pathogen-inoculated and mock-treated *B. juncea* plants

#### 3.5.1 Functional analysis of mock and inoculated *B. juncea* cv. Varuna

Functional enrichment analysis of differentially expressed genes revealed dynamic temporal expression in the Varuna variety at 48 and 96 hours post-inoculation. At 48 hours, downregulated genes were predominantly associated with core cellular processes, including regulation of DNA-templated transcription (86 genes), proteolysis (68 genes), protein ubiquitination (62 genes), mRNA processing (33 genes), and carbohydrate metabolic process (32 genes). Cellular component analysis showed strong enrichment in nucleus (410 genes), membrane (276 genes), cytoplasm (209 genes), and endoplasmic reticulum membrane (45 genes), while molecular functions were enriched in ATP binding (357 genes), metal ion binding (184 genes), DNA binding (156 genes), and RNA binding (94 genes). Pathway analysis highlighted protein modification (78 genes), protein ubiquitination (57 genes), lipid metabolism (14 genes), and significant suppression of pyrimidine metabolism (10 genes) (**Supplementary Figure 13A**). In contrast, upregulated genes at 48 hours were enriched in defense response (40 genes), protein ubiquitination (57 genes), proteolysis (55 genes), and signal transduction (30 genes). Cellular components such as nucleus (305 genes), membrane (254 genes), cytoplasm (157 genes), and plasma membrane (122 genes) were highly represented, with molecular functions dominated by ATP binding (300 genes), zinc ion binding (193 genes), and metal ion binding (143 genes) (**Supplementary Figure 13B**). These patterns indicate an early shift where the plant suppresses certain metabolic and transcriptional activities while activating defense-related protein modification and signaling pathways.

At 96 hours, the response showed both continuity and progression. Down regulated genes continued to show enrichment in carbohydrate metabolic process, defense response, and intracellular protein transport, with prominent representation in the nucleus and plasma membrane (**Supplementary Figure 14A**). Upregulated genes at this later time point exhibited strong enrichment in RNA modification (26 genes), defense response (56 genes), protein ubiquitination (65 genes), and proteolysis (58 genes). Notably, cellular component terms such as nucleus (431 genes), membrane (259 genes), and cytoplasm (189 genes) remained dominant, while molecular functions were heavily enriched in ATP binding (374 genes), Zinc ion binding (217 genes), metal ion binding (158 genes), and kinase activities (146 genes). Pathway analysis at 96 hours further emphasized protein modifications (82 genes) and ubiquitination (57 genes) (**Supplementary Figure 14B**). Overall, these temporal changes demonstrate a progressive transcriptional expression during infection. The Varuna variety suppresses basal metabolic processes while sustaining activation of defense signaling, protein turnover via ubiquitination, and energy reallocation, with the response becoming more pronounced and defense-oriented at 96 hours post-inoculation.

#### 3.5.2 Functional analysis of mock and inoculated *B. juncea* cv. Varuna_WRR

Functional analysis of the top 10 GO terms and Uniprot pathways highlighted the temporal dynamics of transcriptional expression in the Varuna variety following pathogen infection. At 48 hours post-inoculation, up-regulated genes were strongly associated with regulation of DNA-templated transcription (10 genes), proteolysis (30 genes), protein transport (14 genes) and ubiquitination (14 genes), and defense response (10 genes). Cellular component analysis showed prominent enrichment in nucleus (99 genes), membrane (79 genes), plasma membrane (83 genes), and chloroplast (38 genes). Molecular functions were dominated by ATP binding (122 genes), metal ion binding (71 genes), kinase activity (40 genes), and DNA binding (34 genes). Pathway analysis revealed significant involvement of modification (16 genes), protein ubiquitination (8 genes), and glycolysis (3 genes) (**Supplementary Figure 15A**). In contrast, down-regulated genes at 48 h were enriched in translation (34 genes), carbohydrate metabolic process (28 genes), mRNA processing (20 genes), and signal transduction (19 genes), with strong representation in nucleus (285 genes), cytoplasm (151 genes), and plasma membrane (108 genes), and high enrichment in ATP binding (315 genes) and metal ion binding (158 genes) (**Supplementary Figure 15B**).

At 96 hours, the response showed both continuity and progression. Up-regulated genes continued to exhibit strong enrichment in regulation of DNA-templated transcription (101 genes), proteolysis (87 genes), protein ubiquitination (87 genes), and defense response (77 genes), with an even higher number of genes associated with the nucleus (538 genes) and plasma membrane (434 genes). Molecular functions remained dominated by ATP binding (486 genes), DNA binding (162 genes), and kinase activities (176 genes) (**Supplementary Figure 16A**). Down-regulated genes at this later time point were enriched in mRNA processing (17 genes), cell wall organization (25 genes), and intracellular protein transport (28 genes), showing sustained suppression of certain metabolic and ribosomal processes (**Supplementary Figure 16B**). These results demonstrate a progressive and coordinated transcriptional expression during infection. The Varuna_WRR variety rapidly activates defense-related protein turnover (ubiquitination and proteolysis), signaling, and energy metabolism (glycolysis) while suppressing primary carbohydrate metabolism and certain housekeeping functions. The response becomes more pronounced at 96 hours, indicating sustained activation of immune-related processes and resource reallocation from growth to defense, which collectively contribute to the molecular defense strategy of the resistant Varuna variety against the pathogen.

#### 3.5.3 Functional analysis of mock and inoculated *B. juncea* cv. Varuna and Varuna_WRR

Functional enrichment analysis of the top 10 GO terms and pathways revealed distinct transcriptional profiles between susceptible (Varuna) and resistant (Varuna_WRR) lines under mock and pathogen-inoculated conditions. Under mock conditions, genes upregulated in the resistant Varuna_WRR lines were primarily enriched in regulation of DNA-templated transcription (10 genes), proteolysis (30 genes), protein ubiquitination (14 genes), and carbohydrate metabolic process (12 genes). Cellular components such as nucleus (99 genes), cytoplasm (64 genes), and plasma membrane (43 genes) were prominently represented, while molecular functions were dominated by ATP binding (122 genes), metal ion binding (71 genes), and DNA binding (34 genes) (**Supplementary Figure 17A**). This suggests that the resistant genotype constitutively maintains higher expression of genes involved in transcriptional regulation, protein turnover, and energy metabolism even in the absence of pathogen challenge.

In response to pathogen inoculation, the differences became more pronounced. Up-regulated genes in susceptible Varuna showed moderate expression in regulation of DNA-templated transcription (6 genes), proteolysis (8 genes), protein ubiquitination (7 genes), and RNA modification (5 genes). Cellular components including nucleus (32 genes), cytoplasm (16 genes), and plasma membrane (19 genes) remained highly enriched, with molecular functions highlighting ATP binding (33 genes), kinase activity (18 genes), and metal ion binding (20 genes). Pathway analysis further indicated activation of protein ubiquitination (6 genes) and protein modification (7 genes) (**Supplementary Figure 18A**). In contrast, down-regulated genes in the susceptible line under infected conditions were associated with DNA repair (1 gene), protein transport (4 genes), and amino acid metabolism (3 genes), suggesting suppression of certain primary metabolic pathways (**Supplementary Figure 18B**). Comparative analysis across conditions demonstrated that the resistant Varuna_WRR line exhibits a constitutively active transcriptional program centered on protein modification and transcriptional regulation. Upon pathogen challenge, this baseline advantage is further strengthened through enhanced defense signaling, protein ubiquitination, and energy reallocation, while the susceptible line shows relatively lower expression of genes related to defense responses. These findings highlight the molecular basis of enhanced resistance in Varuna_WRR, characterized by both constitutivepriming and a more robust, defense-oriented transcriptional response during infection.

### 3.6 Metabolite profiling of *B. juncea* cv. Varuna vs. Varuna_WRR

Metabolite profiling by GC-MS analysis of trimethylsilyl (TMS) derivatives revealed extensive production of primary metabolism in *B. juncea* leaves following inoculation with *A. candida*. A total of over 250 metabolites were detected across samples, encompassing amino acids and their derivatives, soluble sugars and sugar alcohols, organic acids, polyamines, and phenolic compounds. Normalization to the internal standard enabled semi-quantitative comparison of metabolite abundances between the resistant (varuna_wrr) and susceptible (varuna) genotypes at 24, 48, 72, and 96 hours post-inoculation (hpi).

In the resistant genotype, a rapid and robust accumulation of stress-responsive amino acids was evident as early as 24 hpi. Notably, proline derivatives reached high normalized abundances (L-Proline 2TMS derivative: 6.62 at 24 hpi), accompanied by substantial levels of serine (Serine 3TMS: 5.56), threonine (L-Threonine 3TMS: 2.96), and glycine derivatives. By 48 hpi, glutamine and glutamic acid family metabolites showed strong induction (L-Glutamine 3TMS derivative: 13.29–16.21; L-Glutamic acid 3TMS: 9.80–11.92), indicating enhanced nitrogen assimilation and transport for defense compound synthesis. These amino acids remained elevated at later time points, consistent with sustained osmoprotection, ROS scavenging, and metabolic homeostasis under biotic stress.

Organic acid metabolism was also markedly activated in the resistant interaction. Malic acid (3TMS derivative) accumulated to normalized areas of 2.53 at 24 hpi and 1.41 at 48 hpi, while 2-ketoglutaric acid showed significant increases (up to 2.77). Citric acid and succinic acid derivatives further supported elevated tricarboxylic acid (TCA) cycle flux, likely supplying energy and carbon skeletons for secondary metabolism and antioxidant systems. Myo-inositol (hexa-TMS derivative) was prominently accumulated in resistant samples (normalized area ~12.98 at 24 hpi and ~14.30 at 48 hpi), along with galactinol, pointing to activation of the inositol phosphate signaling and raffinose family oligosaccharide pathways for osmoprotection and cell wall reinforcement. Carbohydrate profiles in the resistant genotype were characterized by high levels of sucrose (8TMS derivative: 19.99–22.20), trehalose (7TMS: 4.71), and various fructose and glucose methyloxime derivatives. These sugars likely served dual roles as compatible solutes and energy reserves, with controlled partitioning preventing excessive pathogen exploitation. Polyamine metabolism was also induced, with notable putrescine (4TMS) and spermine derivatives detected at elevated levels, supporting membrane stability and signaling.

In contrast, the susceptible genotype exhibited a delayed and attenuated defense-oriented metabolic response. Early time points (24–48 hpi, where available) showed lower relative accumulations of proline, serine, glutamine, and malic acid compared to the resistant counterpart. Instead, susceptible plants displayed progressive shifts toward sugar alcohols (e.g., adonitol, dulcitol, erythritol) and complex oligosaccharides (turanose, maltose, and melibiose derivatives) at 72 and 96 hpi. Sucrose levels were high but showed different temporal dynamics, often accompanied by increased fructose and glucose derivatives that may reflect enhanced sink strength or cell wall degradation favoring pathogen nutrition. Myo-inositol accumulation was comparatively modest, and TCA cycle intermediates were less pronounced.

**Figure 3:**
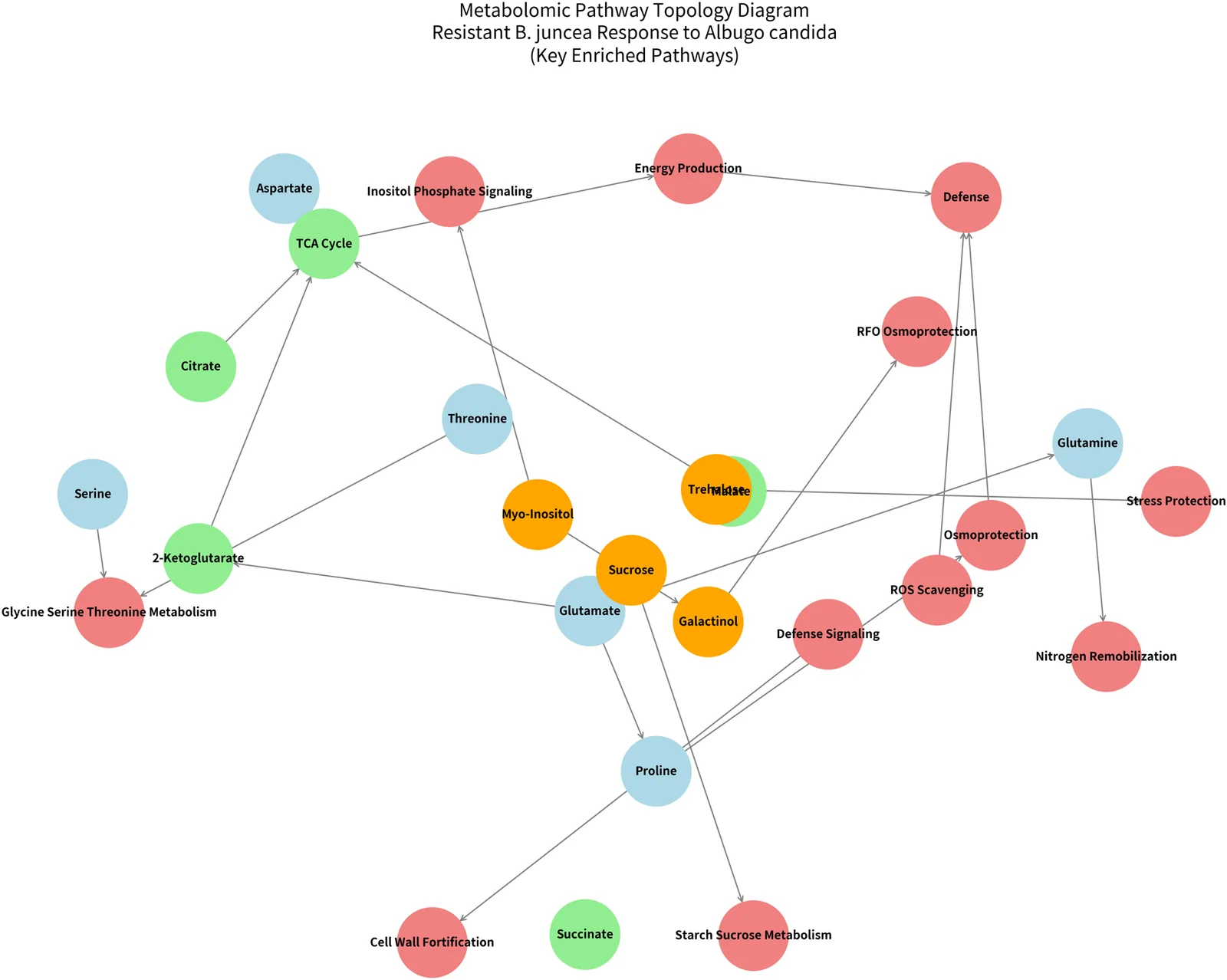
Pathway topology diagram showing major enriched metabolic pathways in resistant *Brassica juncea*cv. Varuna_WRR in response to *A. candida* infection after 24, 48. 72, and 96 hours.Node size and edge thickness are proportional to biological importance based on metabolite abundance and pathway impact. This diagram was constructed based on normalized abundances of key metabolites (e.g., Proline = 6.62, Glutamine = 13.29–16.21, Myo-inositol = 12.98–14.30) that were significantly higher in the resistant genotype at 24–48 hpi.

Multivariate patterns (inferred from abundance matrices) indicated clear separation between genotypes, with resistant samples clustering by rapid induction of amino acid, organic acid, and inositol pathways, while susceptible samples were distinguished by late-stage carbohydrate and polyol accumulation. Key differential metabolites included proline, glutamine, malic acid, myo-inositol, and serine, which consistently showed higher normalized abundances in the resistant genotype during the critical early infection phase (24–48 hpi). These metabolic signatures align with effective activation of defense, antioxidant, and osmoprotective mechanisms that likely restrict *A. candida* colonization in the resistant variety. These findings provide a comprehensive metabolic framework for understanding genotype-specific responses to *A. candida* infection (**Figure 3**).

## 4 Discussion

*Albugo candida* is an obligate biotrophic oomycete that infects more than 63 genera and 241 species within the Brassicaceae family globally, causing white rust and staghead diseases (Saharan and Verma, 1992; Choi et al., 2007; Singh et al., 2021b). Notably, the pathogen can also infect certain non-Brassicaceae hosts, broadening its host range and economic impact (Choi et al., 2008, 2009). In the present study, the resistance locus AcB1-A5.1, originating from the east European gene pool line Donskaja-IV of *B. juncea*, conferred complete resistance against six different Indian isolates of *A. candida*. This locus harbors a single dominant CC-NB-LRR resistance gene, BjuWRR1, which when introduced into the susceptible Indian mustard cultivar Varuna through genetic transformation, successfully conferred resistance to all tested isolates (Arora et al., 2019). These findings underscore the value of utilizing wild and exotic germplasm for strengthening resistance in cultivated Brassica species. Successful infection by *A. candida* in susceptible Brassica cultivars induces profound biochemical and physiological changes that facilitate pathogen establishment and disease progression. In contrast, resistant cultivars, including the Varuna_WRR line examined here, generally maintain higher levels of chlorophyll, sugars, and total phenolic content across growth stages, supporting sustained photosynthetic capacity and antioxidant defense. Susceptible cultivars, however, exhibit increased accumulation of total proteins and free amino acids throughout the growth cycle, potentially reflecting stress-induced proteolysis and metabolic dysregulation (Singh, 2000).

Miyaji et al. (2023) previously demonstrated differential responses in komatsuna (*B. rapa* var. perviridis) cultivars, identifying key time points (48 and 72 hours post-inoculation) for transcriptomic divergence between resistant and susceptible genotypes. Building upon this, our study provides integrated multi-omics insights into the BjuWRR1-mediated resistance in B. juncea. To elucidate the molecular basis of resistance, we performed comparative transcriptomic and metabolomic analyses. At 48 hours post-inoculation, transcriptomic reprogramming was markedly different between the resistant Varuna_WRR and susceptible Varuna lines. While both genotypes mounted an initial response, the resistant line exhibited stronger upregulation of defense-related processes, including cell wall macromolecule biosynthesis, calcium and manganese ion signaling, SCF-dependent ubiquitin-proteasome pathway, SCF ubiquitin ligase complex, protein phosphorylation, and programmed cell death (Alejandro et al., 2020; Han et al., 2024; Lim-Hing et al., 2024; Vijayan and Raji, 2025). Gene ontology enrichment analysis further highlighted activation of defense response to bacteria and hypersensitive response pathways. Metabolomically, the resistant line accumulated higher levels of protective carbohydrates such as sucrose, fructose, glucose, and galactinol, along with several amino acids. These metabolites likely contribute to osmotic adjustment, ROS scavenging, and signaling functions that bolster early defense (Formela-Luboińska et al., 2020). In contrast, the susceptible line displayed a larger number of downregulated genes enriched in core cellular processes such as RNA polymerase II assembly, mRNA processing, translational initiation, histone acetylation, and auxin transport, indicating a broader suppression of growth and housekeeping functions (Bolton, 2009).

A central mechanism underlying this resistance involves the interconnected roles of specific amino acids and organic acids as metabolic hubs. The amino acids proline, serine, and glutamine, together with malic acid, function as key integrators that amplify salicylic acid (SA), ethylene (ET), and jasmonic acid (JA) signaling during biotic stress in Brassica species. Proline accumulation, driven by upregulation of P5CS2 (the Brassica ortholog of AtP5CS2) during incompatible pathogen interactions, serves not only as an osmoprotectant and ROS scavenger but also as a signaling molecule that maintains redox homeostasis and reinforces SA-mediated hypersensitive response (HR) and systemic acquired resistance (SAR). Its biosynthesis from glutamate is tightly linked to glutamine metabolism, which supplies nitrogen for rapid defense protein synthesis and pathogenesis-related (PR) gene expression (Fabro et al., 2004; Funck et al., 2020; Hayat et al., 2012; Novakova et al., 2014).

In *B. napus* and related species, defense against diverse pathogens such as *Sclerotinia sclerotiorum* and *Plasmodiophora brassicae* is orchestrated through finely tuned hormonal signaling networks dominated by SA, ET, and JA pathways. SA, synthesized primarily via the isochorismate synthase (ICS) pathway from chorismate, acts as a master regulator of biotrophic and hemibiotrophic pathogen defense by activating NPR1-dependent expression of PR1, PR2, and PR5 genes. This leads to antimicrobial protein accumulation, cell wall fortification, and establishment of SAR in distal tissues (Wang et al., 2012; Novakova et al., 2014; Galindo-González et al., 2020; Zhou et al., 2020; Rai et al., 2024; Fang et al., 2025). Glutamine accumulation further enhances the activity of the GS/GOGAT cycle, supporting efficient nitrogen assimilation and recycling under stress conditions. This sustains the glutamate pool necessary for proline biosynthesis while providing nitrogen for defense protein and secondary metabolite production (Novakova et al., 2014; Fortunato et al., 2023).

Complementing SA signaling, the ET pathway, initiated from methionine through ACC synthase (ACS) and ACC oxidase (ACO), plays a pivotal role by inactivating the negative regulator CTR1, stabilizing EIN2, and activating EIN3/EIL1 transcription factors that drive ethylene response factor (ERF) gene expression. This cascade induces genes involved in ROS modulation, phytoalexin production, and glucosinolate biosynthesis, processes of particular importance in Brassicaceae (Li et al., 2019; Ali et al., 2025). JA and ET pathways frequently act synergistically against necrotrophic pathogens, while context-dependent crosstalk with SA (typically antagonistic toward biotrophs but permissive against necrotrophs) enables precise fine-tuning of immune responses (Li et al., 2019; Chang-long et al., 2021). Serine contributes to these networks through one-carbon metabolism and photorespiration, serving as a precursor for cysteine and glutathione biosynthesis essential for ROS detoxification, as well as acting as a defense-priming signal via glutamate receptor-like channels and calcium-dependent protein kinases (Jez, 2019; Rosa-Téllez et al., 2024). Furthermore, root-exuded malic acid recruits beneficial rhizobacteria such as *Bacillus subtilis*, triggering induced systemic resistance (ISR) that integrates with JA/ET signaling and indirectly bolsters SA-mediated defenses (Rudrappa et al., 2008; Galindo-González et al., 2020).

At the later stage of 96 hours post-inoculation, the resistant genotype sustained and amplified its defense-oriented response. Transcriptomic data revealed continued upregulation of SA signaling, protein ubiquitination, carbohydrate metabolism, and chromatin organization, with changes of greater magnitude compared to the susceptible line. Metabolite profiles reinforced this advantage, showing sustained elevation of raffinose family oligosaccharides, trehalose, galactinol, proline, and several organic acids. This temporal stability indicates effective long-term metabolic reprogramming and resource reallocation toward immunity, whereas the susceptible variety exhibited signs of metabolic exhaustion, including greater downregulation of primary carbohydrate metabolism and weaker activation of defense metabolites (Loedolff, 2015; Zhou et al., 2025). The coordinated transcriptomic and metabolomic responses observed in Varuna_WRR highlight several key resistance mechanisms. Upregulation of the ubiquitin-proteasome system at both transcriptional and functional levels likely facilitates rapid turnover of regulatory proteins during defense activation. Enhanced carbohydrate metabolism and accumulation of sugar alcohols (galactinol, myo-inositol) provide energy, osmoprotection, and signaling molecules, aligning with findings in clubroot-resistant Brassica lines where raffinose family oligosaccharides play central roles in stress tolerance (Kaur et al., 2025). Constitutive priming, combined with infection-induced reinforcement of calcium signaling, ethylene response, and programmed cell death pathways, further strengthens the resistant phenotype (Zhang et al., 2014, 2026). These results are consistent with integrated omics studies in *B. napus*, in which resistant genotypes against Sclerotinia and blackleg pathogens exhibit early activation of phenylpropanoid and ubiquitin pathways alongside sustained osmoprotectant accumulation (Wu et al., 2016; Amjadi et al., 2024). The metabolic plasticity of Varuna_WRR, characterized by higher baseline levels of protective metabolites and more robust induction upon infection, mirrors mechanisms reported across resistant Brassicaceae members (Chakraborty et al., 2021; Shaw et al., 2021).

In conclusion, the superior resistance of Varuna_WRR against *A. candida* is driven by a sophisticated dual-layer strategy involving constitutive metabolic priming and highly coordinated, sustained transcriptomic-metabolic reprogramming that prioritizes defense over growth. This integrated response enables efficient resource allocation, cellular protection, and long-term defense maintenance during pathogen challenge. The annotated genes and metabolites identified in this study, particularly those involved in galactinol synthesis, proline metabolism, ubiquitin ligase complexes, and hormonal crosstalk, represent promising molecular targets for marker-assisted breeding programs. Ultimately, these insights contribute to the development of high-yielding, white rust-resistant Brassica varieties with enhanced broad-spectrum biotic stress tolerance, addressing a critical challenge in sustainable oilseed production.

## Data Availability

The datasets presented in this study can be found in online repositories. The names of the repository/repositories and accession number(s) can be found below: https://www.ncbi.nlm.nih.gov/, GSM9665563 https://www.ncbi.nlm.nih.gov/, GSM9665562 https://www.ncbi.nlm.nih.gov/, GSM9665560 https://www.ncbi.nlm.nih.gov/, GSM9665564 https://www.ncbi.nlm.nih.gov/, GSM9665558 https://www.ncbi.nlm.nih.gov/, GSM9665565 https://www.ncbi.nlm.nih.gov/, GSM9665559 https://www.ncbi.nlm.nih.gov/, GSM9665561.

## Supplementary material

Supplementary Figures 1–6 Differential gene expression analysis between mock and infected *B. juncea* cv. Varuna and *B. juncea* cv. Varuna_WRR varieties samples at 48 and 96 hours post-inoculation. **(A)** Number of upregulated (red) and downregulated (cyan) differentially expressed genes (DEGs). **(B)** Volcano plot showing log_2_ fold change versus –log_10_(adjusted p-value). Red and blue dots represent significantly up- and downregulated genes, respectively. **(C)** Heatmap showing expression patterns of selected DEGs across mock and infected replicates. Red and blue colors indicate up- and downregulation, respectively.

Supplementary Figures 7–12 Gene Ontology (GO) and KEGG pathway enrichment analysis of differentially expressed genes in response to pathogen infection in *B. juncea* cv. Varuna and *B. juncea* cv. Varuna_WRR varieties samples at 48 and 96 hours post-inoculation. **(A)** Biological Process **(B)** Cellular Component **(C)** Molecular Function and **(D)** KEGG Pathways. Dot size represents the number of genes, color intensity indicates FDR value, and the x-axis shows fold enrichment.

Supplementary Figures 13–18 Top 10 enriched GO terms and Uniprot pathways of up- and downregulated genes between Varuna and Varuna_WRR lines under mock and pathogen-inoculated conditions after 48 and 96 hours. Bar graphs represent the number of genes associated with each **(A)** Biological process, **(B)** Cellular component, **(C)** molecular function, and **(D)** Uniprot pathway.
