# Supplementary material for: Multi-omics analysis to uncover constitutive priming and dynamic metabolic reprogramming conferring white rust resistance in Brassica juncea
Source: Front Plant Sci. 2026 Aug 19;17:1918745. doi: 10.3389/fpls.2026.1918745 (PMC13534074; doi:10.3389/fpls.2026.1918745)
Supplement: Supplementary file 1 [file DataSheet1.pdf]

Figure. S1a

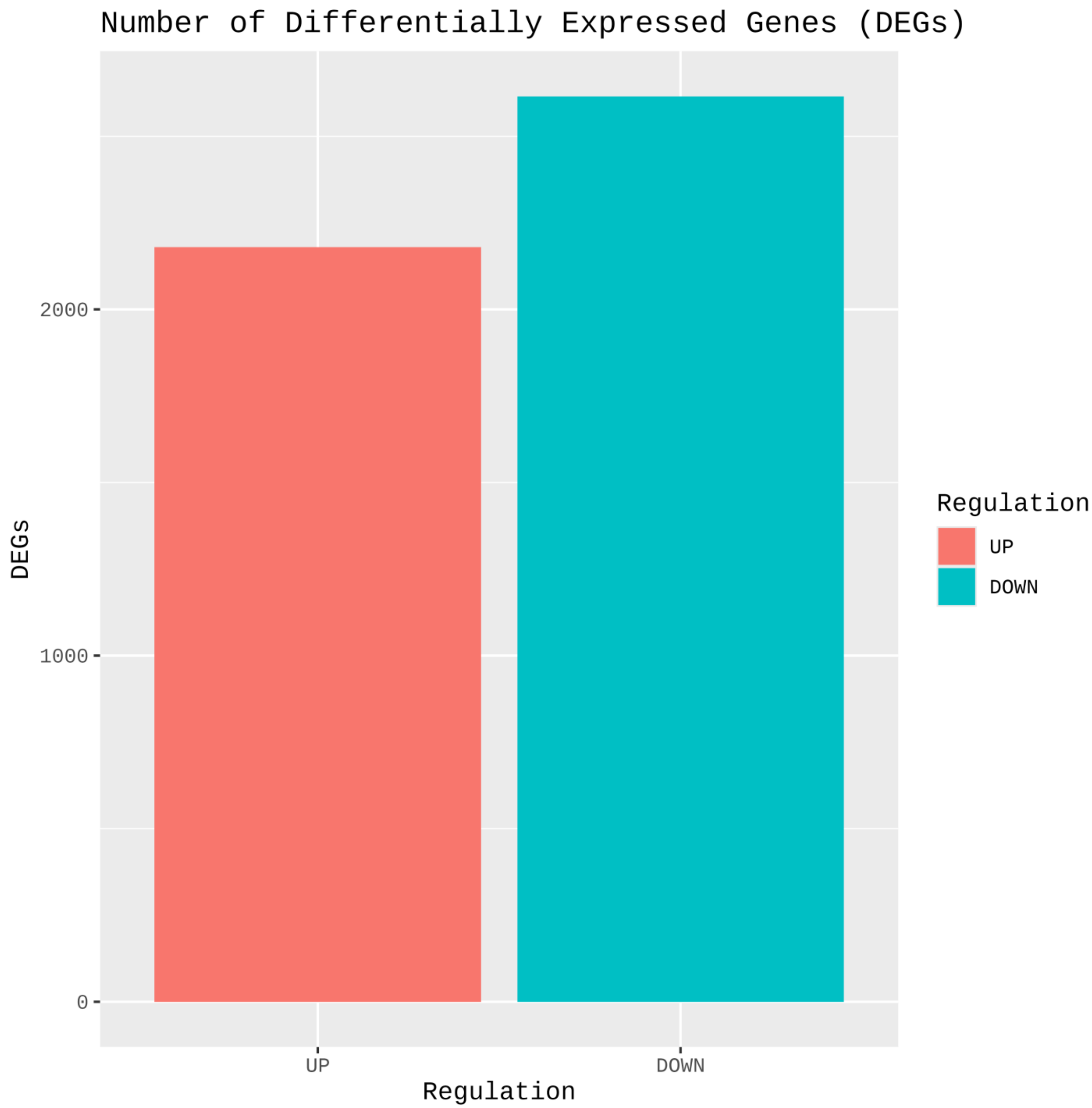
Number of Differentially Expressed Genes (DEGs)
DEGs
2000
1000
0
UP
DOWN
Regulation
Regulation
UP
DOWN

Figure. S1b

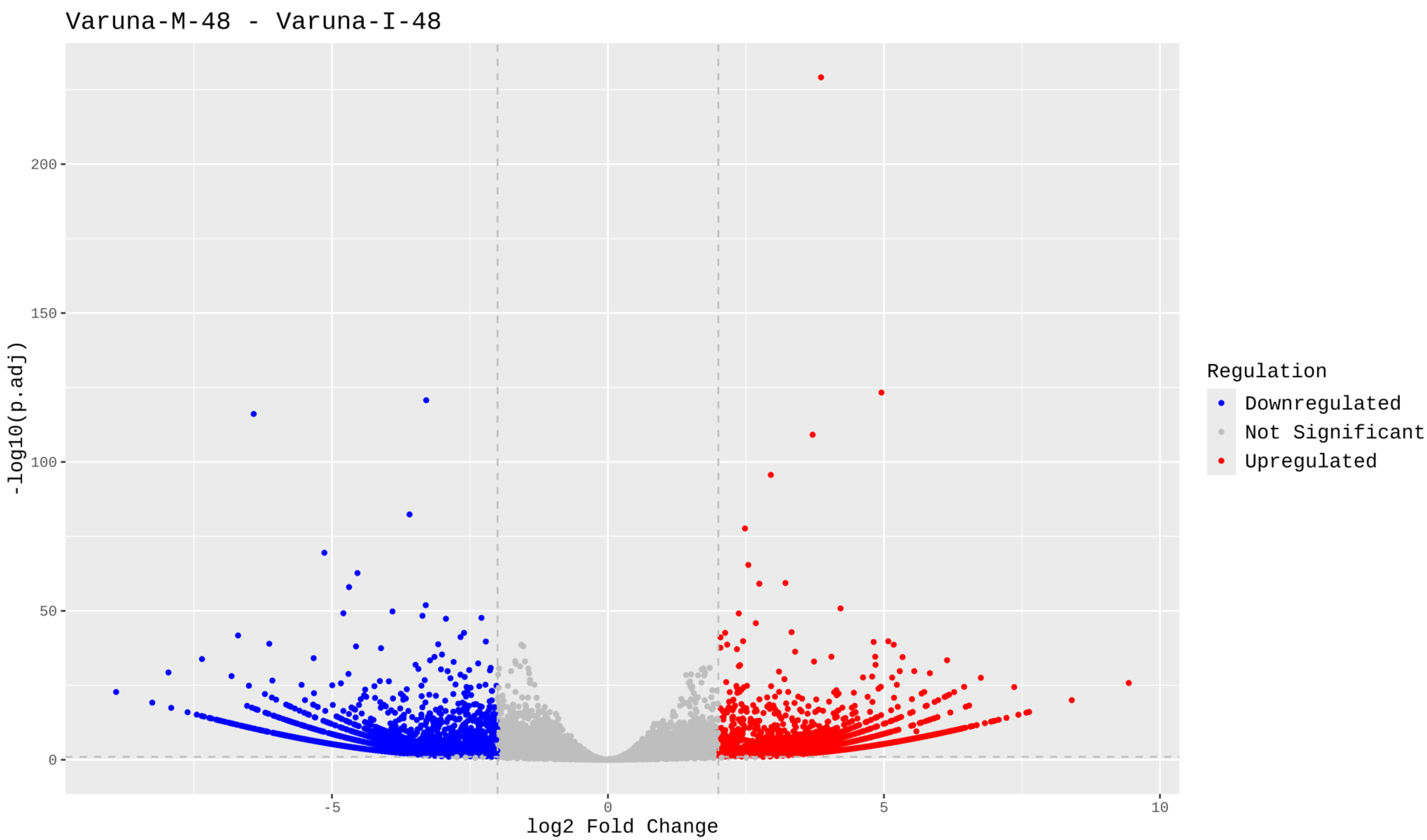
Varuna-M-48 - Varuna-I-48
-log10(p.adj)
200
150
100
50
0
-5
0
5
10
log2 Fold Change
Regulation
Downregulated
Not Significant
Upregulated

Figure. S1c

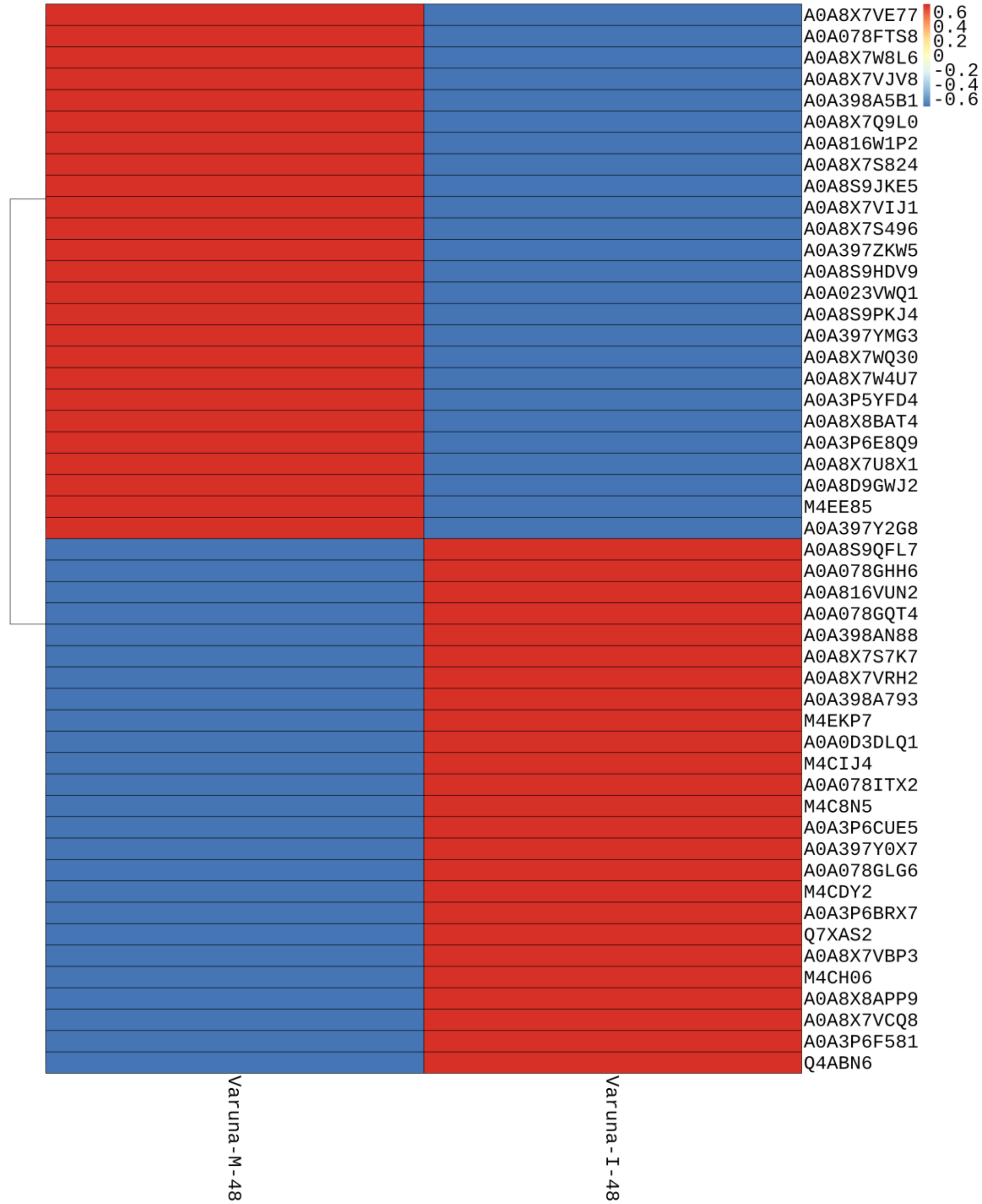
0.6
0.4
0.2
0
-0.2
-0.4
-0.6
A0A8X7VE77
A0A078FTS8
A0A8X7W8L6
A0A8X7VJV8
A0A398A5B1
A0A8X7Q9L0
A0A816W1P2
A0A8X7S824
A0A8S9JKE5
A0A8X7VIJ1
A0A8X7S496
A0A397ZKW5
A0A8S9HDV9
A0A023VWQ1
A0A8S9PKJ4
A0A397YMG3
A0A8X7WQ30
A0A8X7W4U7
A0A3P5YFD4
A0A8X8BAT4
A0A3P6E8Q9
A0A8X7U8X1
A0A8D9GWJ2
M4EE85
A0A397Y2G8
A0A8S9QFL7
A0A078GHH6
A0A816VUN2
A0A078GQT4
A0A398AN88
A0A8X7S7K7
A0A8X7VRH2
A0A398A793
M4EKP7
A0A0D3DLQ1
M4CIJ4
A0A078ITX2
M4C8N5
A0A3P6CUE5
A0A397Y0X7
A0A078GLG6
M4CDY2
A0A3P6BRX7
Q7XAS2
A0A8X7VBP3
M4CH06
A0A8X8APP9
A0A8X7VCQ8
A0A3P6F581
Q4ABN6
Varuna-M-48
Varuna-I-48

Figure. S2a

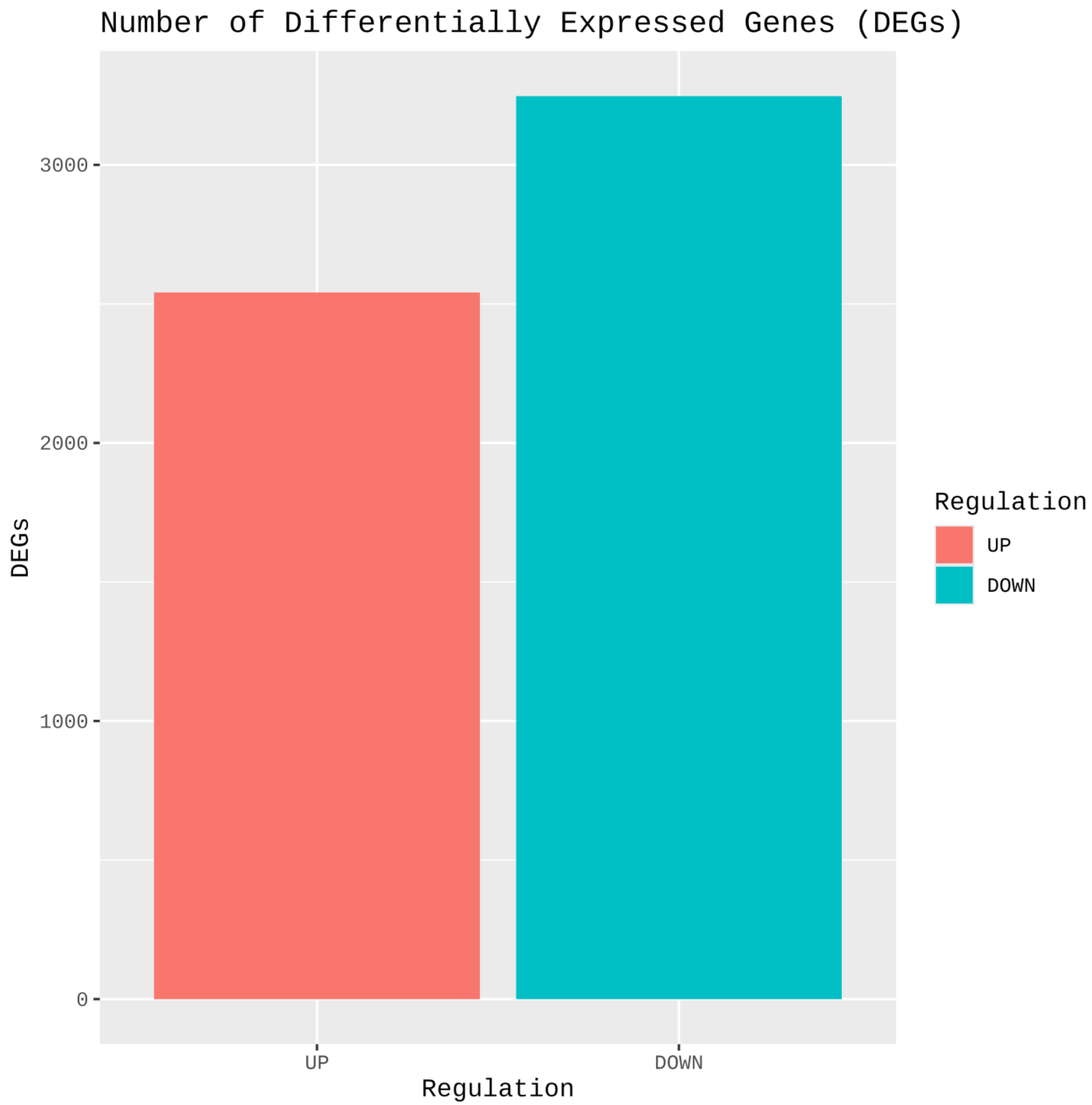

Number of Differentially Expressed Genes (DEGs)
DEGs
3000
2000
1000
0
UP
DOWN
Regulation
Regulation
UP
DOWN

Figure. S2b

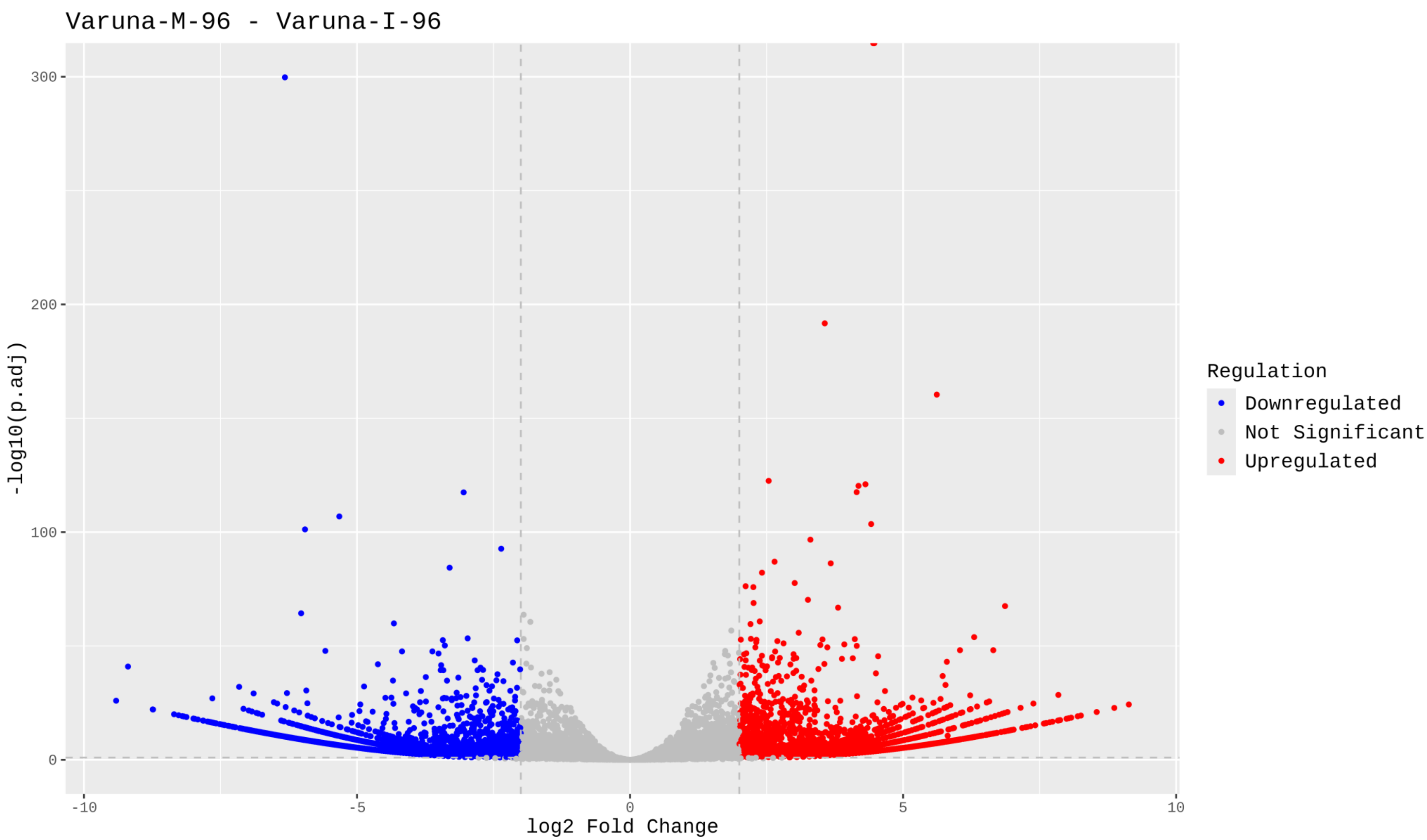

Varuna-M-96 - Varuna-I-96
300
200
100
0
-log10(p.adj)
-10
-5
0
5
10
log2 Fold Change
Regulation
Downregulated
Not Significant
Upregulated

Figure. S2c

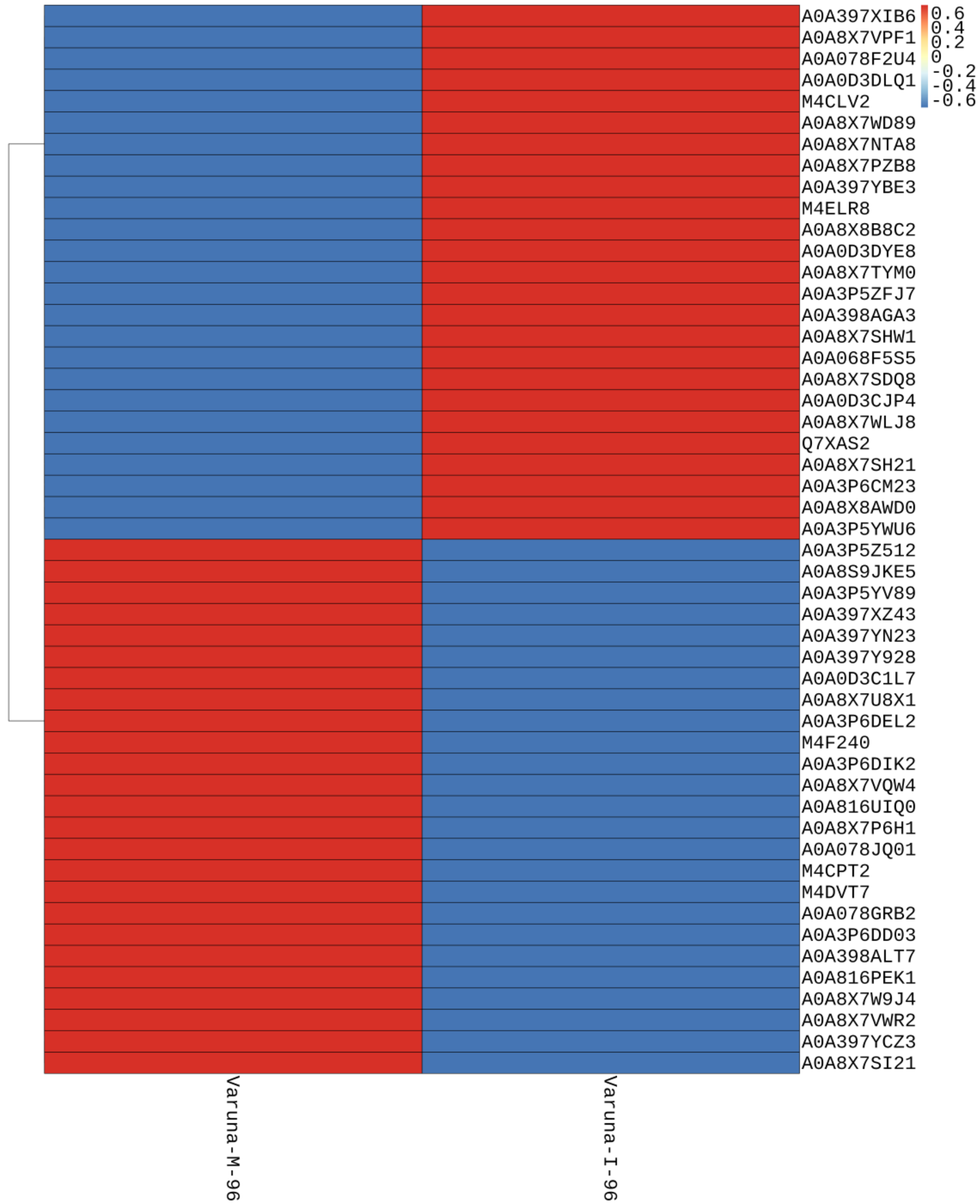
A0A397XIB6
A0A8X7VPF1
A0A078F2U4
A0A0D3DLQ1
M4CLV2
A0A8X7WD89
A0A8X7NTA8
A0A8X7PZB8
A0A397YBE3
M4ELR8
A0A8X8B8C2
A0A0D3DYE8
A0A8X7TYM0
A0A3P5ZFJ7
A0A398AGA3
A0A8X7SHW1
A0A068F5S5
A0A8X7SDQ8
A0A0D3CJP4
A0A8X7WLJ8
Q7XAS2
A0A8X7SH21
A0A3P6CM23
A0A8X8AWD0
A0A3P5YWU6
A0A3P5Z512
A0A8S9JKE5
A0A3P5YV89
A0A397XZ43
A0A397YN23
A0A397Y928
A0A0D3C1L7
A0A8X7U8X1
A0A3P6DEL2
M4F240
A0A3P6DIK2
A0A8X7VQW4
A0A816UIQ0
A0A8X7P6H1
A0A078JQ01
M4CPT2
M4DVT7
A0A078GRB2
A0A3P6DD03
A0A398ALT7
A0A816PEK1
A0A8X7W9J4
A0A8X7VWR2
A0A397YCZ3
A0A8X7SI21
0.6
0.4
0.2
0
-0.2
-0.4
-0.6
Varuna-M-96
Varuna-I-96

Figure. S3a

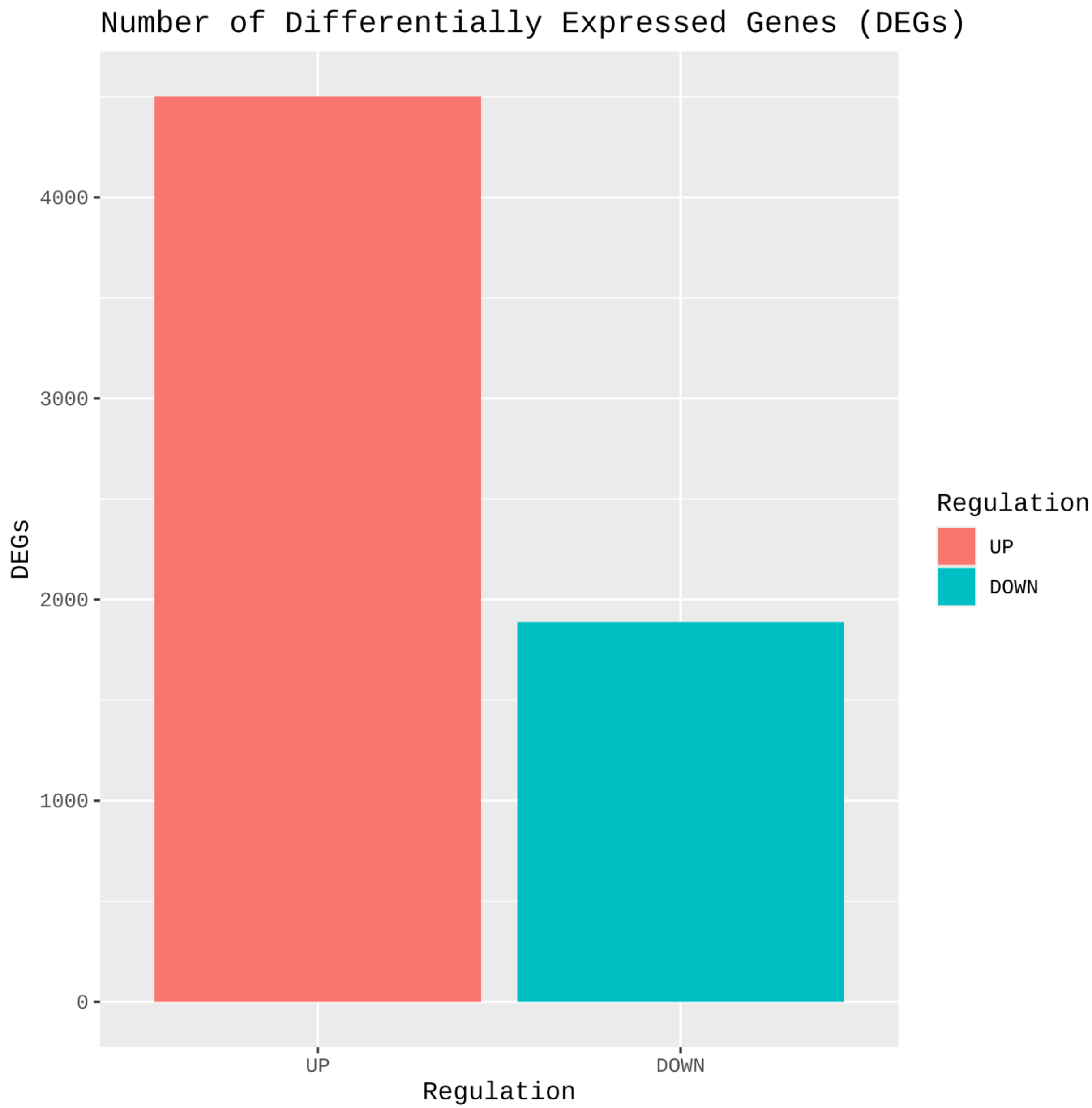

Number of Differentially Expressed Genes (DEGs)
4000
3000
2000
1000
0
DEGs
UP
DOWN
Regulation
Regulation
UP
DOWN

Figure. S3b

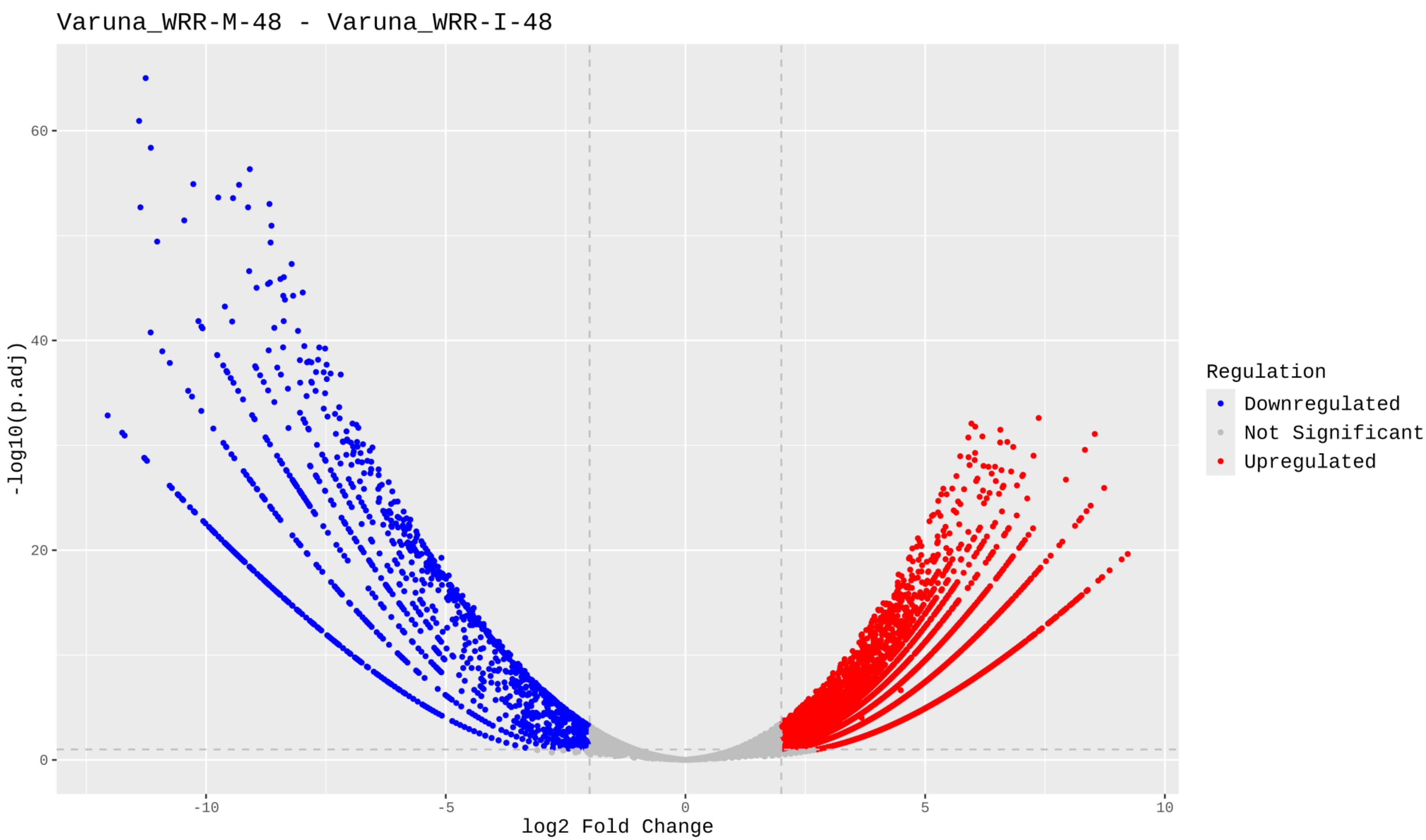

Varuna_WRR-M-48 - Varuna_WRR-I-48
-log10(p.adj)
60
40
20
0
-10
-5
0
5
10
log2 Fold Change
Regulation
Downregulated
Not Significant
Upregulated

Figure. S3c

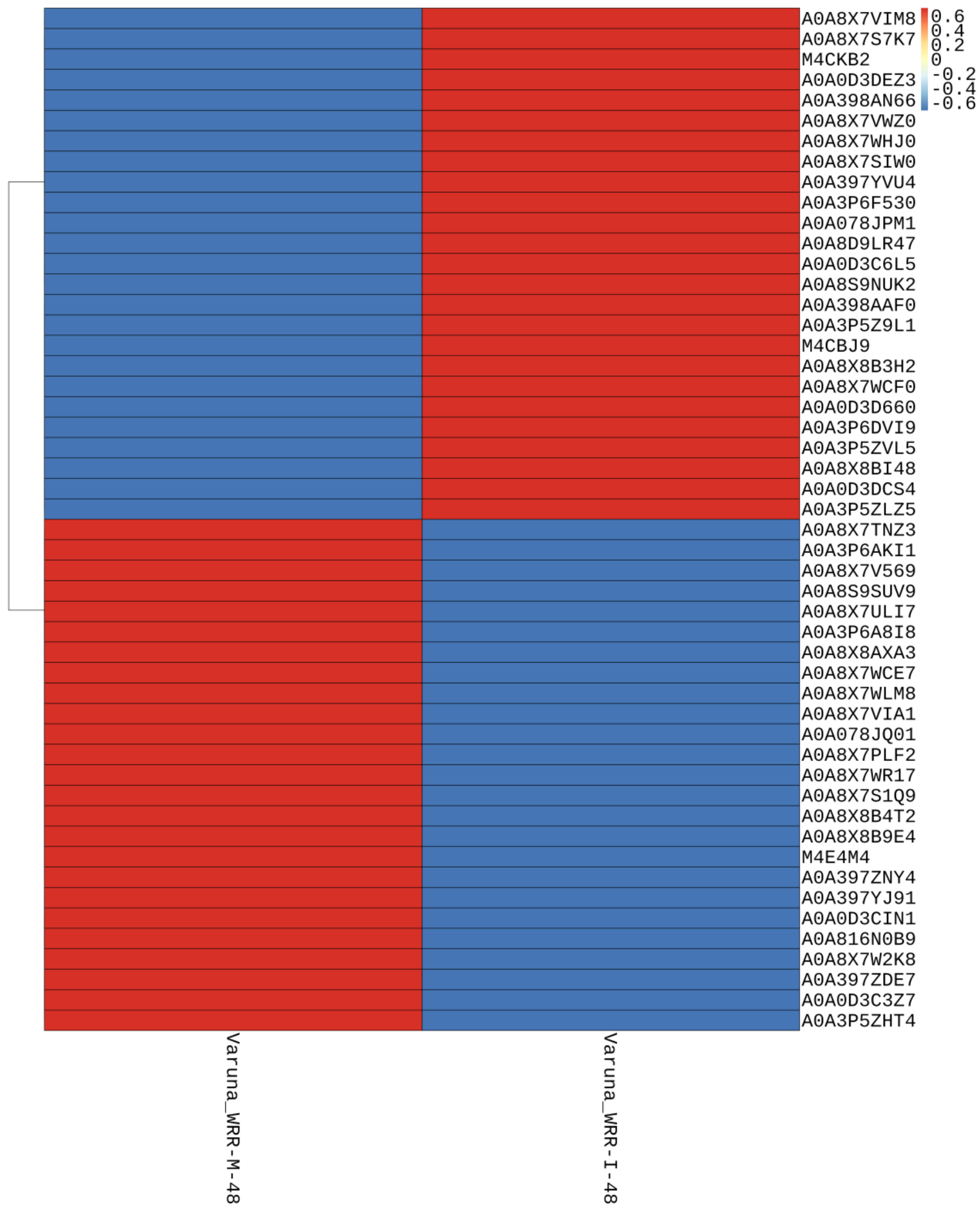

A0A8X7VIM8
A0A8X7S7K7
M4CKB2
A0A0D3DEZ3
A0A398AN66
A0A8X7VWZ0
A0A8X7WHJ0
A0A8X7SIW0
A0A397YVU4
A0A3P6F530
A0A078JPM1
A0A8D9LR47
A0A0D3C6L5
A0A8S9NUK2
A0A398AAF0
A0A3P5Z9L1
M4CBJ9
A0A8X8B3H2
A0A8X7WCF0
A0A0D3D660
A0A3P6DVI9
A0A3P5ZVL5
A0A8X8BI48
A0A0D3DCS4
A0A3P5ZLZ5
A0A8X7TNZ3
A0A3P6AKI1
A0A8X7V569
A0A8S9SUV9
A0A8X7ULI7
A0A3P6A8I8
A0A8X8AXA3
A0A8X7WCE7
A0A8X7WLM8
A0A8X7VIA1
A0A078JQ01
A0A8X7PLF2
A0A8X7WR17
A0A8X7S1Q9
A0A8X8B4T2
A0A8X8B9E4
M4E4M4
A0A397ZNY4
A0A397YJ91
A0A0D3CIN1
A0A816N0B9
A0A8X7W2K8
A0A397ZDE7
A0A0D3C3Z7
A0A3P5ZHT4
0.6
0.4
0.2
0
-0.2
-0.4
-0.6
Varuna_WRR-M-48
Varuna_WRR-I-48

Figure. S4a

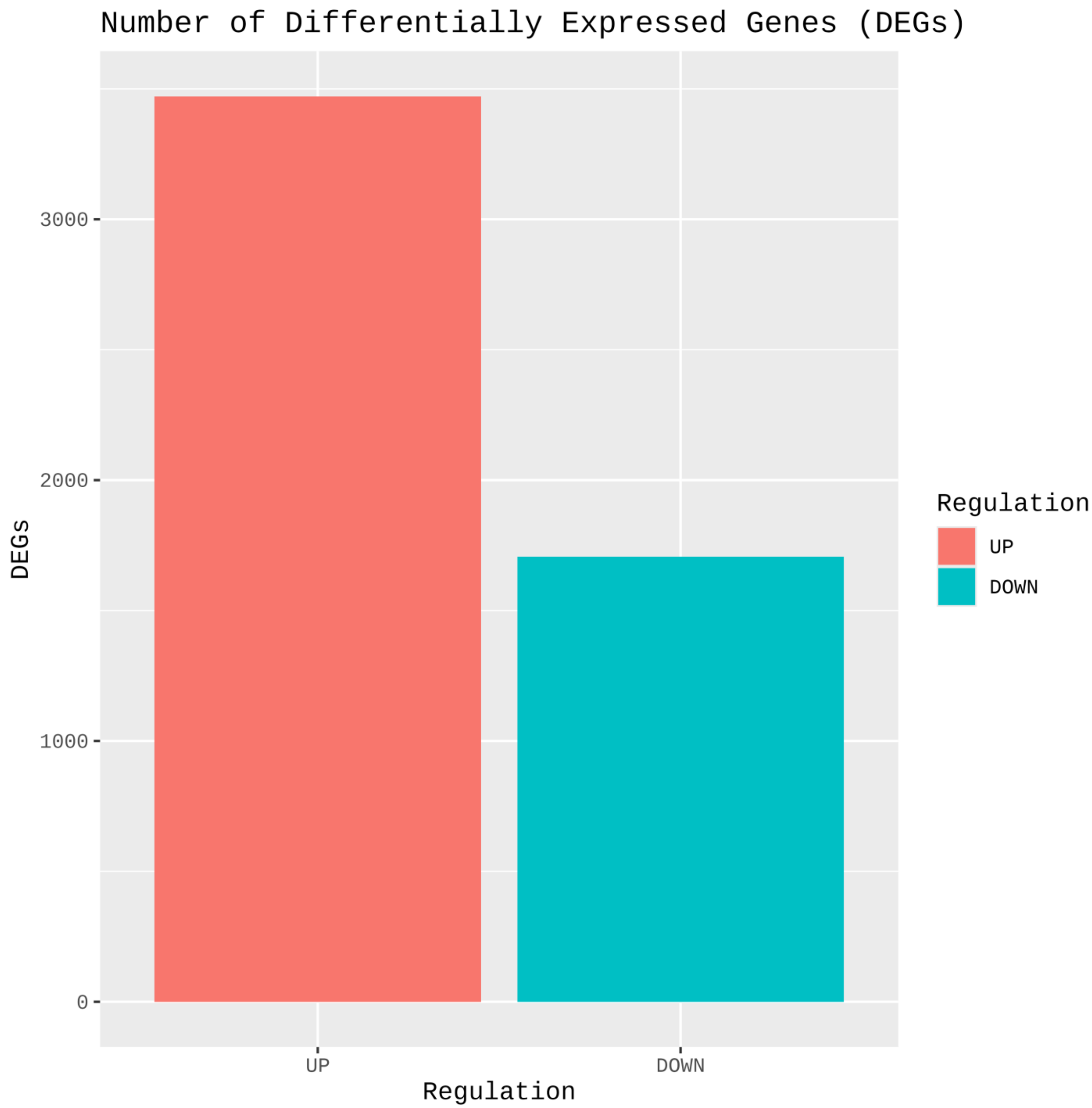
Number of Differentially Expressed Genes (DEGs)
DEGs
3000
2000
1000
0
UP
DOWN
Regulation
Regulation
UP
DOWN

Figure. S4b

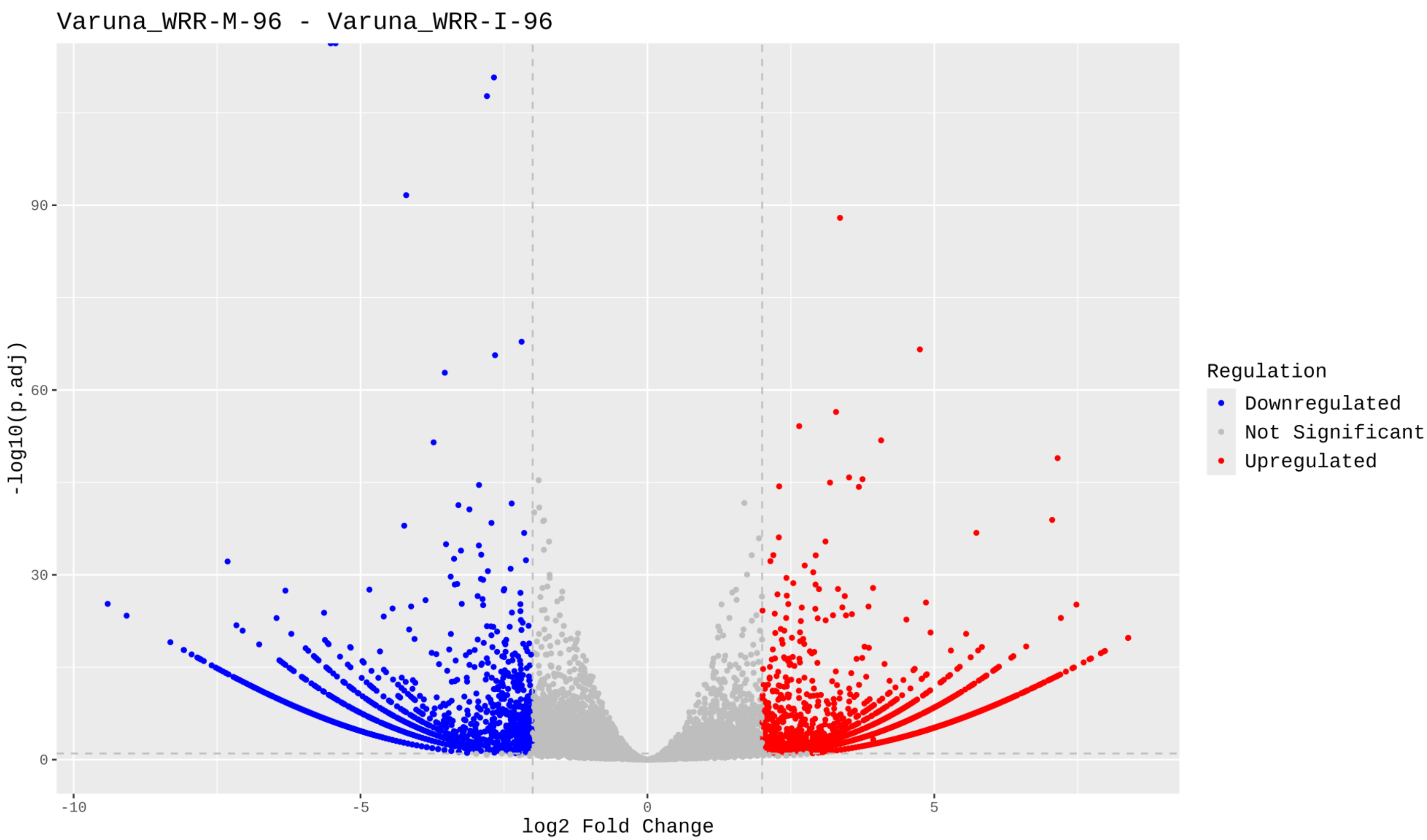
Varuna_WRR-M-96 - Varuna_WRR-I-96
-log10(p.adj)
90
60
30
0
-10
-5
0
5
log2 Fold Change
Regulation
Downregulated
Not Significant
Upregulated

Figure. S4c

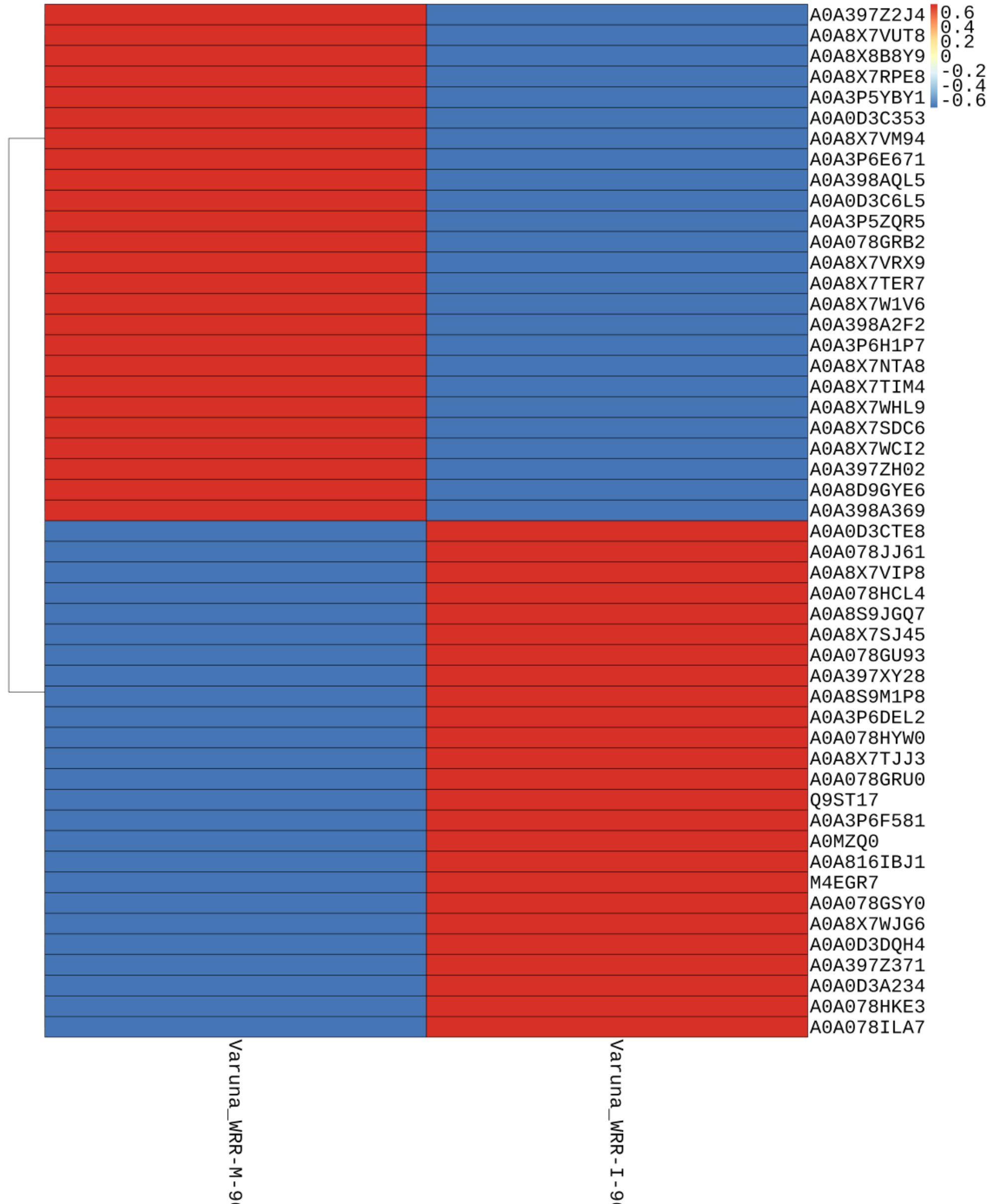
0.6
0.4
0.2
0
-0.2
-0.4
-0.6
A0A397Z2J4
A0A8X7VUT8
A0A8X8B8Y9
A0A8X7RPE8
A0A3P5YBY1
A0A0D3C353
A0A8X7VM94
A0A3P6E671
A0A398AQL5
A0A0D3C6L5
A0A3P5ZQR5
A0A078GRB2
A0A8X7VRX9
A0A8X7TER7
A0A8X7W1V6
A0A398A2F2
A0A3P6H1P7
A0A8X7NTA8
A0A8X7TIM4
A0A8X7WHL9
A0A8X7SDC6
A0A8X7WCI2
A0A397ZH02
A0A8D9GYE6
A0A398A369
A0A0D3CTE8
A0A078JJ61
A0A8X7VIP8
A0A078HCL4
A0A8S9JGQ7
A0A8X7SJ45
A0A078GU93
A0A397XY28
A0A8S9M1P8
A0A3P6DEL2
A0A078HYW0
A0A8X7TJJ3
A0A078GRU0
Q9ST17
A0A3P6F581
A0MZQ0
A0A816IBJ1
M4EGR7
A0A078GSY0
A0A8X7WJG6
A0A0D3DQH4
A0A397Z371
A0A0D3A234
A0A078HKE3
A0A078ILA7
Varuna_WRR-M-96
Varuna_WRR-I-96

Figure. S5a

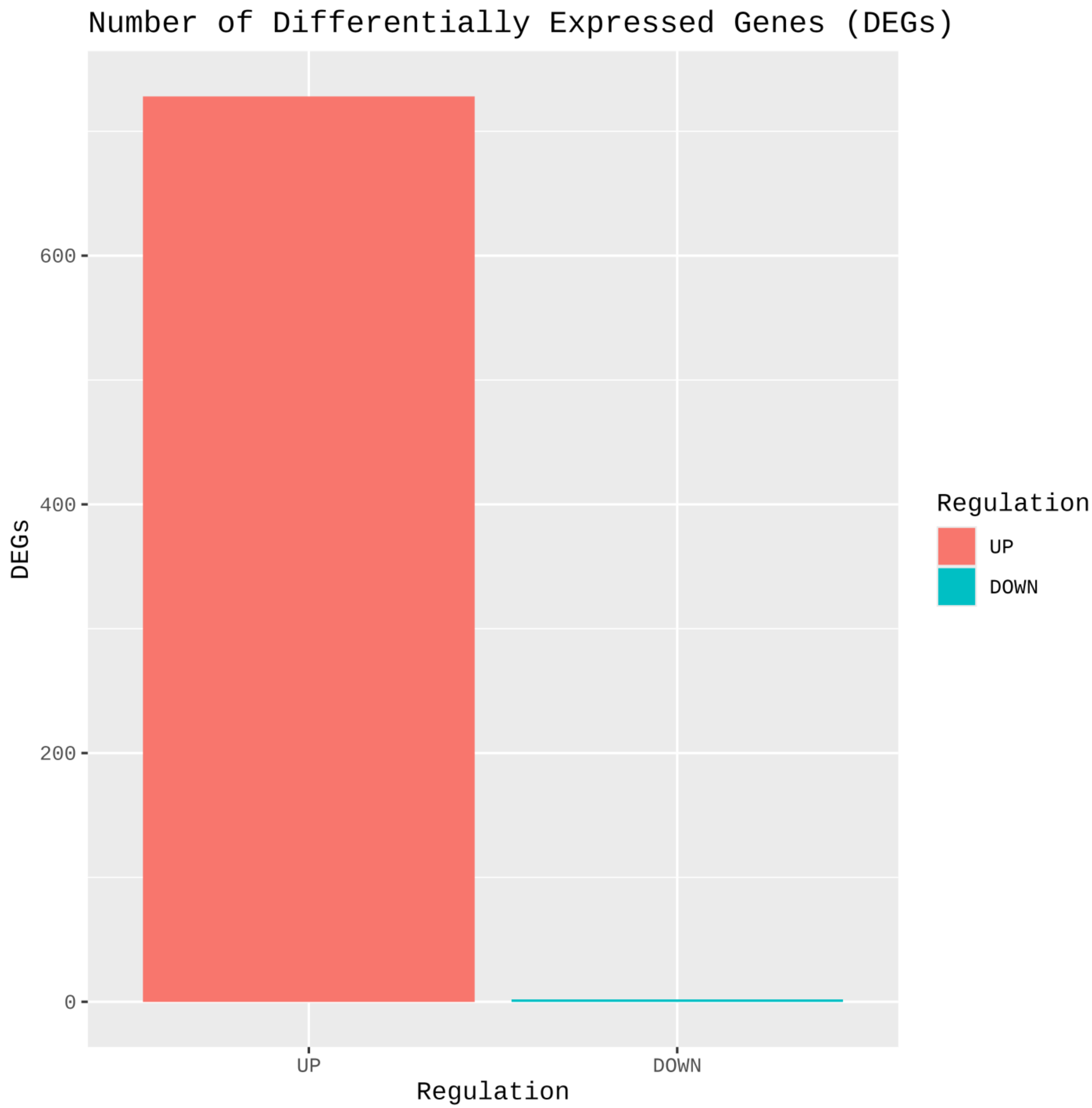
Number of Differentially Expressed Genes (DEGs)
DEGs
600
400
200
0
UP
DOWN
Regulation
Regulation
UP
DOWN

Figure. S5b

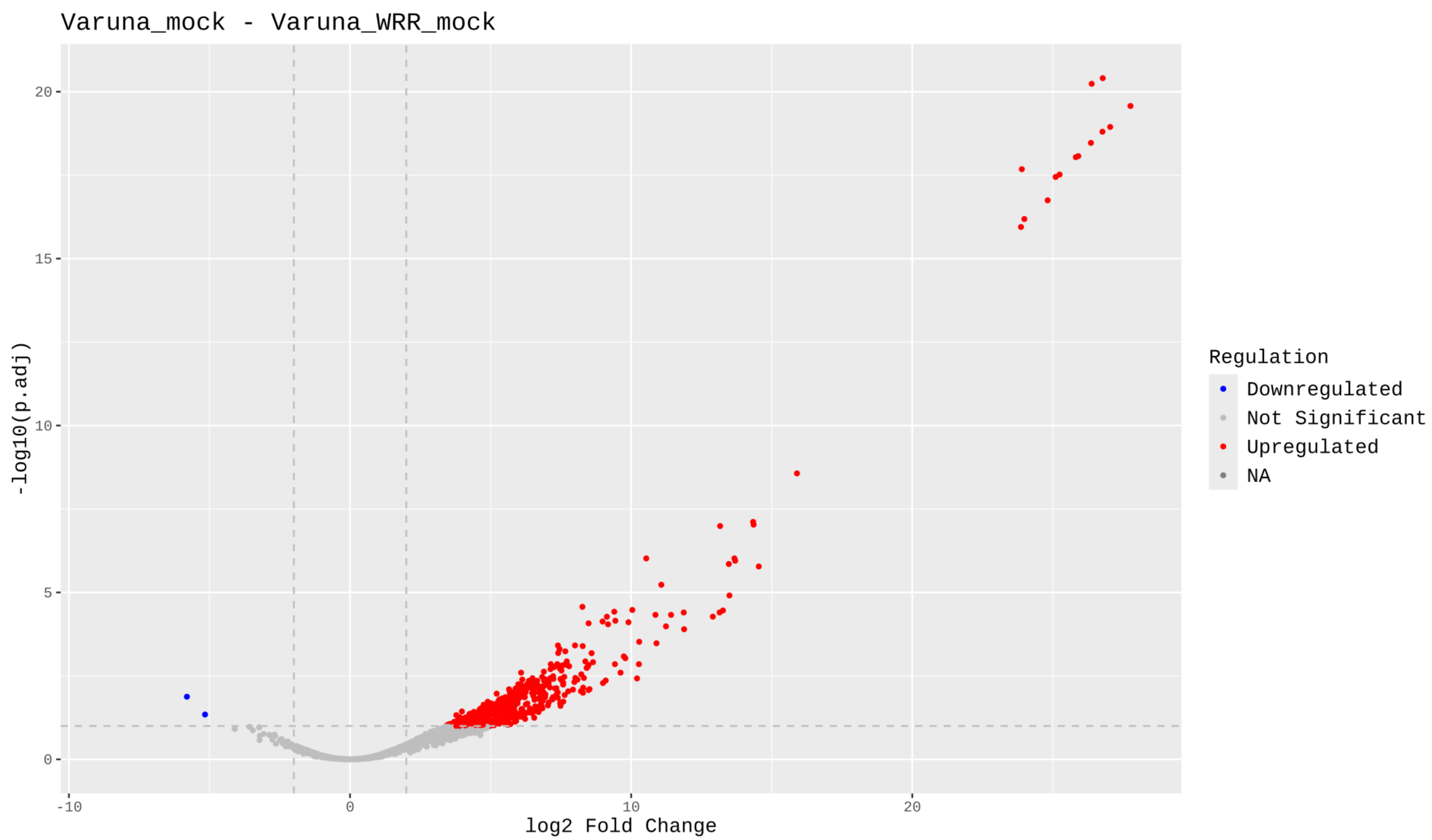

Varuna_mock - Varuna_WRR_mock
-log10(p.adj)
20
15
10
5
0
-10
0
10
20
log2 Fold Change
Regulation
Downregulated
Not Significant
Upregulated
NA

Figure. S5c

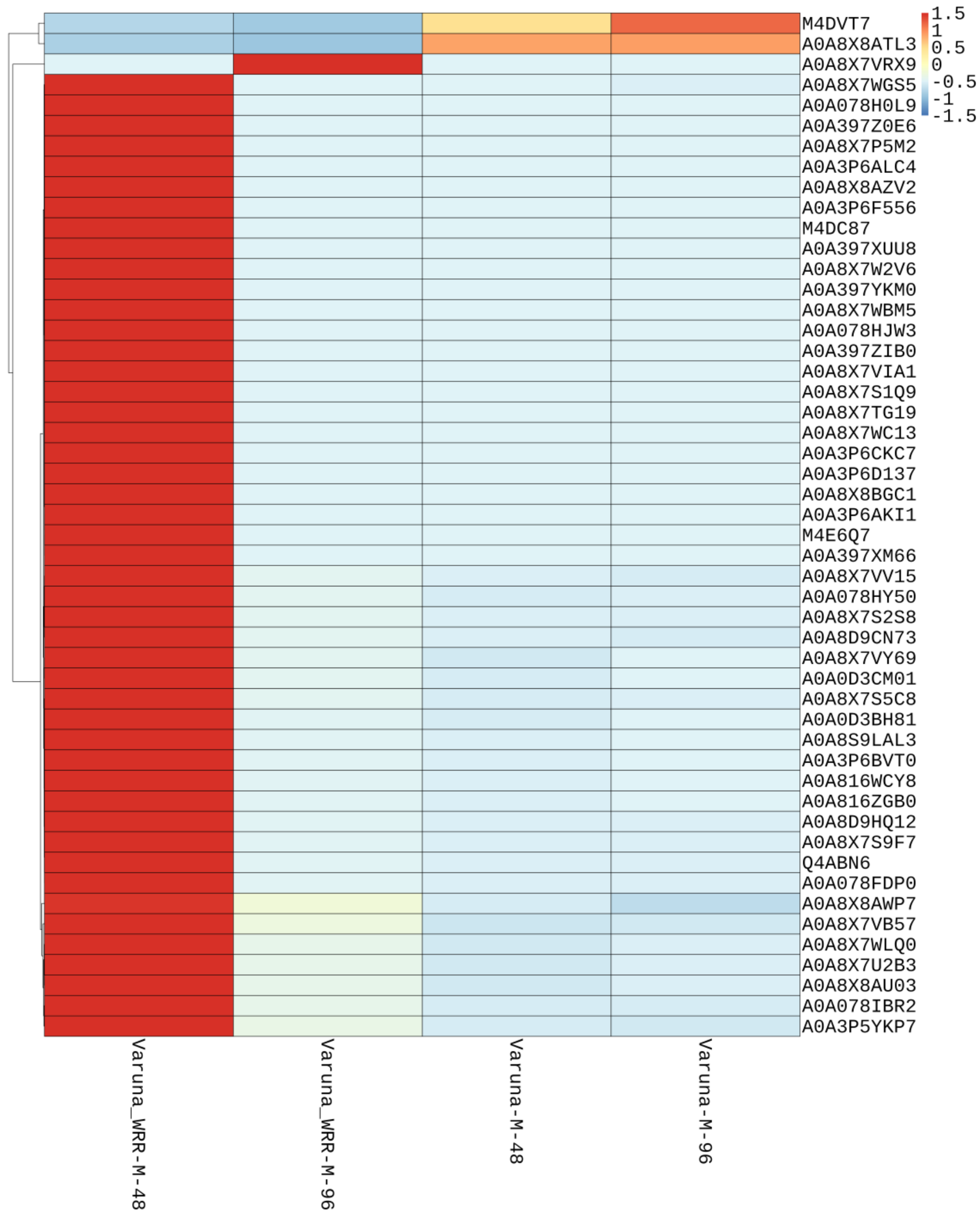
M4DVT7
A0A8X8ATL3
A0A8X7VRX9
A0A8X7WGS5
A0A078H0L9
A0A397Z0E6
A0A8X7P5M2
A0A3P6ALC4
A0A8X8AZV2
A0A3P6F556
M4DC87
A0A397XUU8
A0A8X7W2V6
A0A397YKM0
A0A8X7WBM5
A0A078HJW3
A0A397ZIB0
A0A8X7VIA1
A0A8X7S1Q9
A0A8X7TG19
A0A8X7WC13
A0A3P6CKC7
A0A3P6D137
A0A8X8BGC1
A0A3P6AKI1
M4E6Q7
A0A397XM66
A0A8X7VV15
A0A078HY50
A0A8X7S2S8
A0A8D9CN73
A0A8X7VY69
A0A0D3CM01
A0A8X7S5C8
A0A0D3BH81
A0A8S9LAL3
A0A3P6BVT0
A0A816WCY8
A0A816ZGB0
A0A8D9HQ12
A0A8X7S9F7
Q4ABN6
A0A078FDP0
A0A8X8AWP7
A0A8X7VB57
A0A8X7WLQ0
A0A8X7U2B3
A0A8X8AU03
A0A078IBR2
A0A3P5YKP7
1.5
1
0.5
0
-0.5
-1
-1.5
Varuna_WRR-M-48
Varuna_WRR-M-96
Varuna-M-48
Varuna-M-96

Figure. S6a

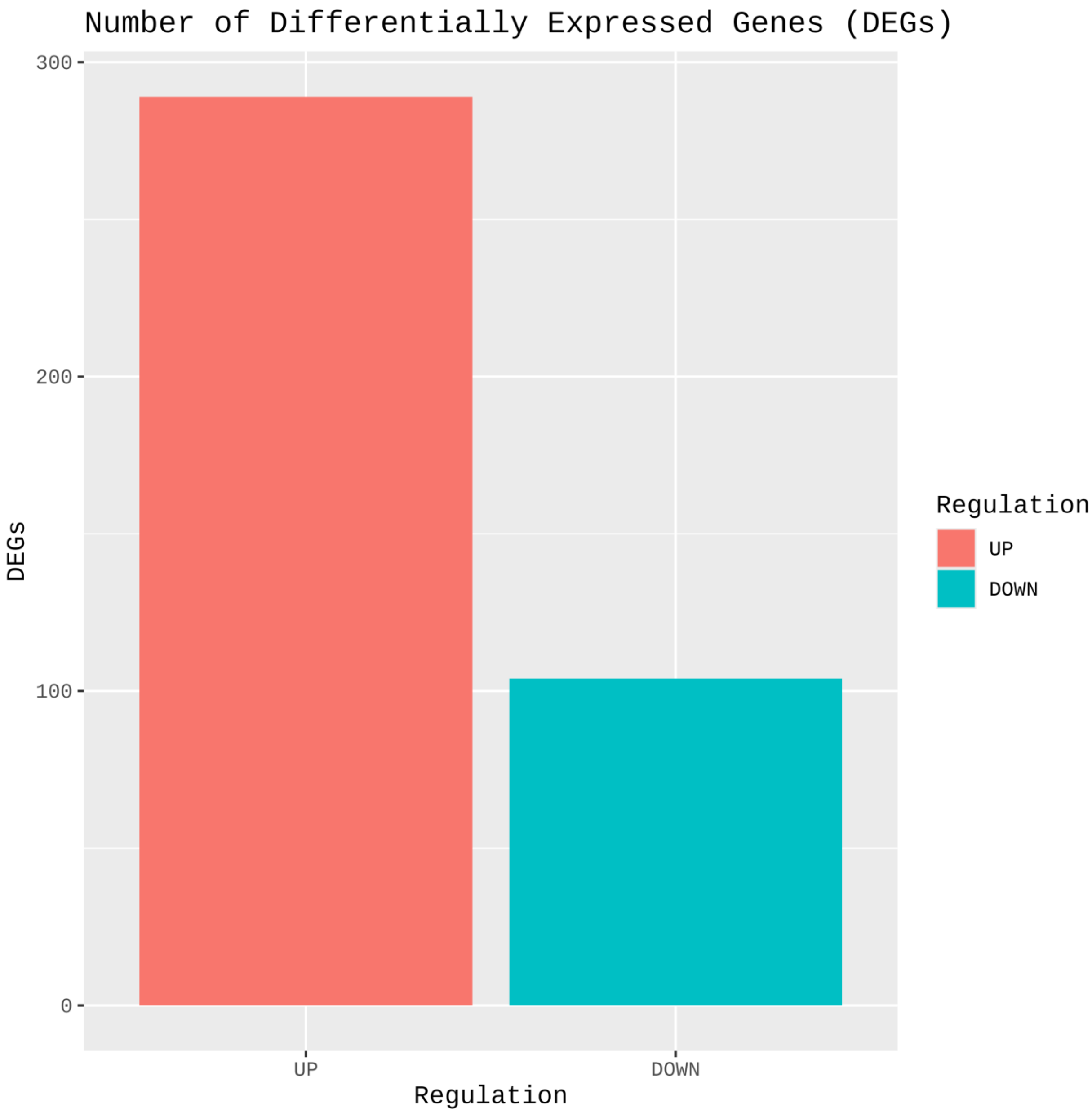

Number of Differentially Expressed Genes (DEGs)
300
200
100
0
DEGs
UP
DOWN
Regulation
Regulation
UP
DOWN

Figure. S6b

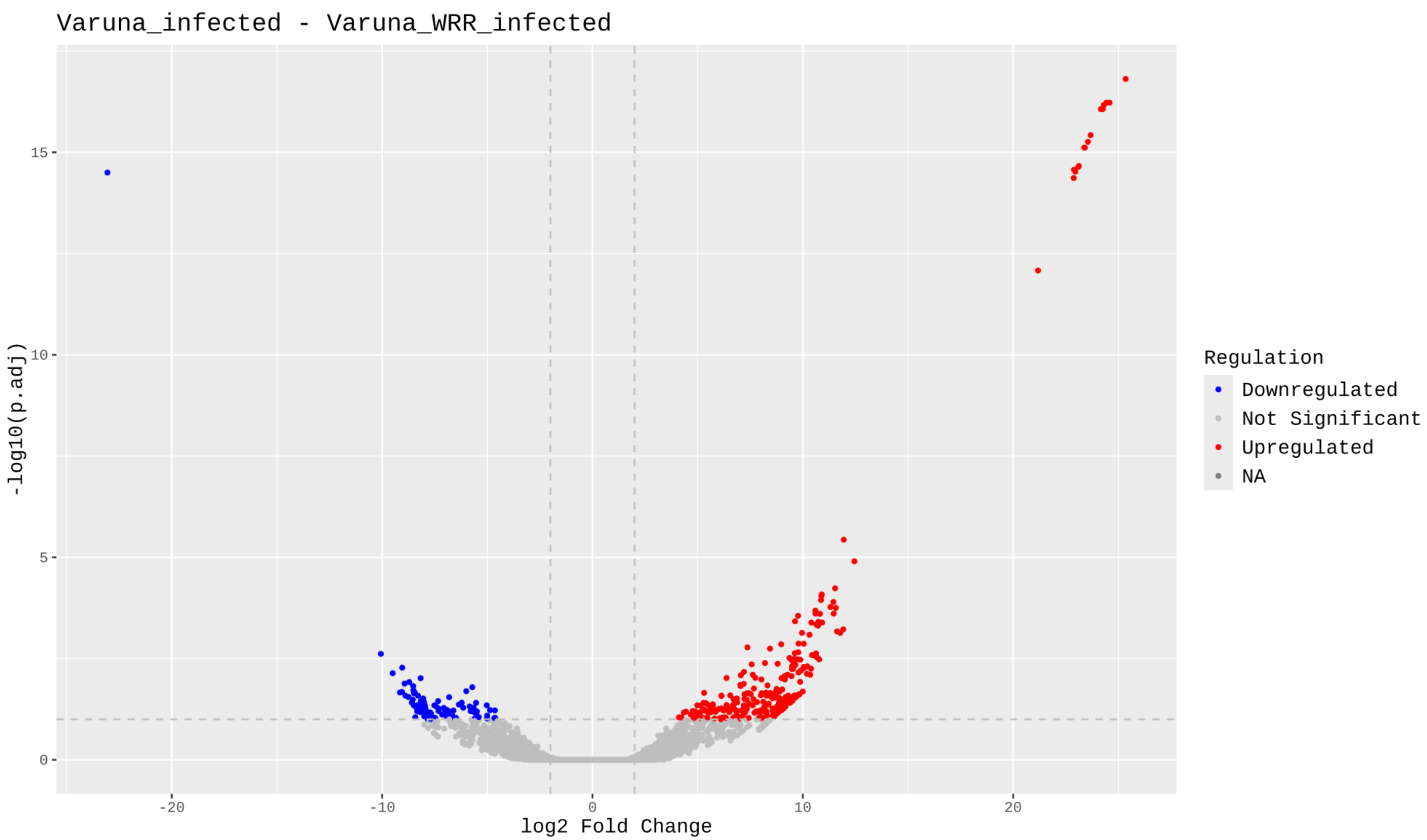

Varuna_infected - Varuna_WRR_infected
-log10(p.adj)
15
10
5
0
-20
-10
0
10
20
log2 Fold Change
Regulation
Downregulated
Not Significant
Upregulated
NA

Figure. S6c

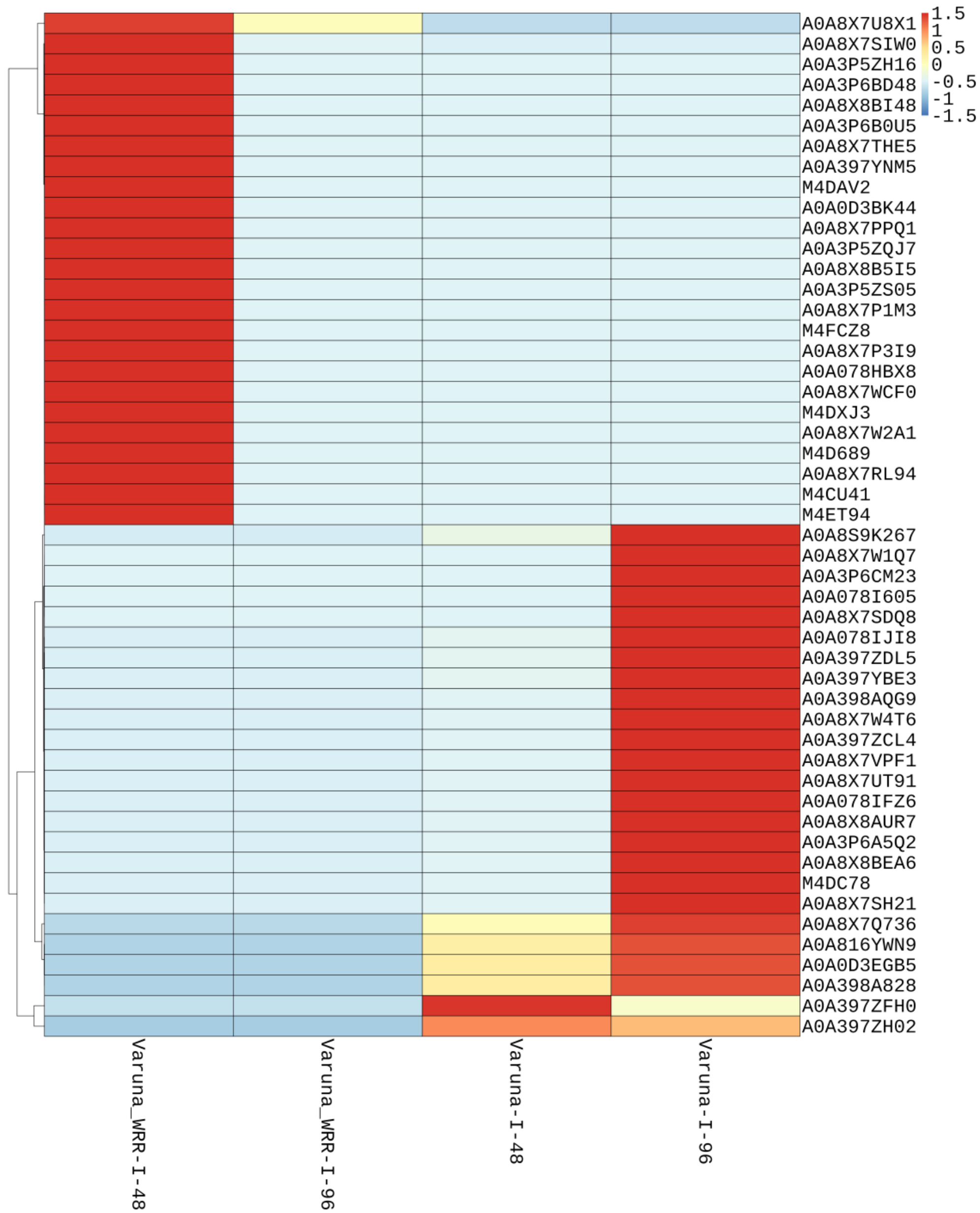
1.5
1
0.5
0
-0.5
-1
-1.5
A0A8X7U8X1
A0A8X7SIW0
A0A3P5ZH16
A0A3P6BD48
A0A8X8BI48
A0A3P6B0U5
A0A8X7THE5
A0A397YNM5
M4DAV2
A0A0D3BK44
A0A8X7PPQ1
A0A3P5ZQJ7
A0A8X8B5I5
A0A3P5ZS05
A0A8X7P1M3
M4FCZ8
A0A8X7P3I9
A0A078HBX8
A0A8X7WCF0
M4DXJ3
A0A8X7W2A1
M4D689
A0A8X7RL94
M4CU41
M4ET94
A0A8S9K267
A0A8X7W1Q7
A0A3P6CM23
A0A078I605
A0A8X7SDQ8
A0A078IJI8
A0A397ZDL5
A0A397YBE3
A0A398AQG9
A0A8X7W4T6
A0A397ZCL4
A0A8X7VPF1
A0A8X7UT91
A0A078IFZ6
A0A8X8AUR7
A0A3P6A5Q2
A0A8X8BEA6
M4DC78
A0A8X7SH21
A0A8X7Q736
A0A816YWN9
A0A0D3EGB5
A0A398A828
A0A397ZFH0
A0A397ZH02
Varuna_WRR-I-48
Varuna_WRR-I-96
Varuna-I-48
Varuna-I-96

Figure. S7a

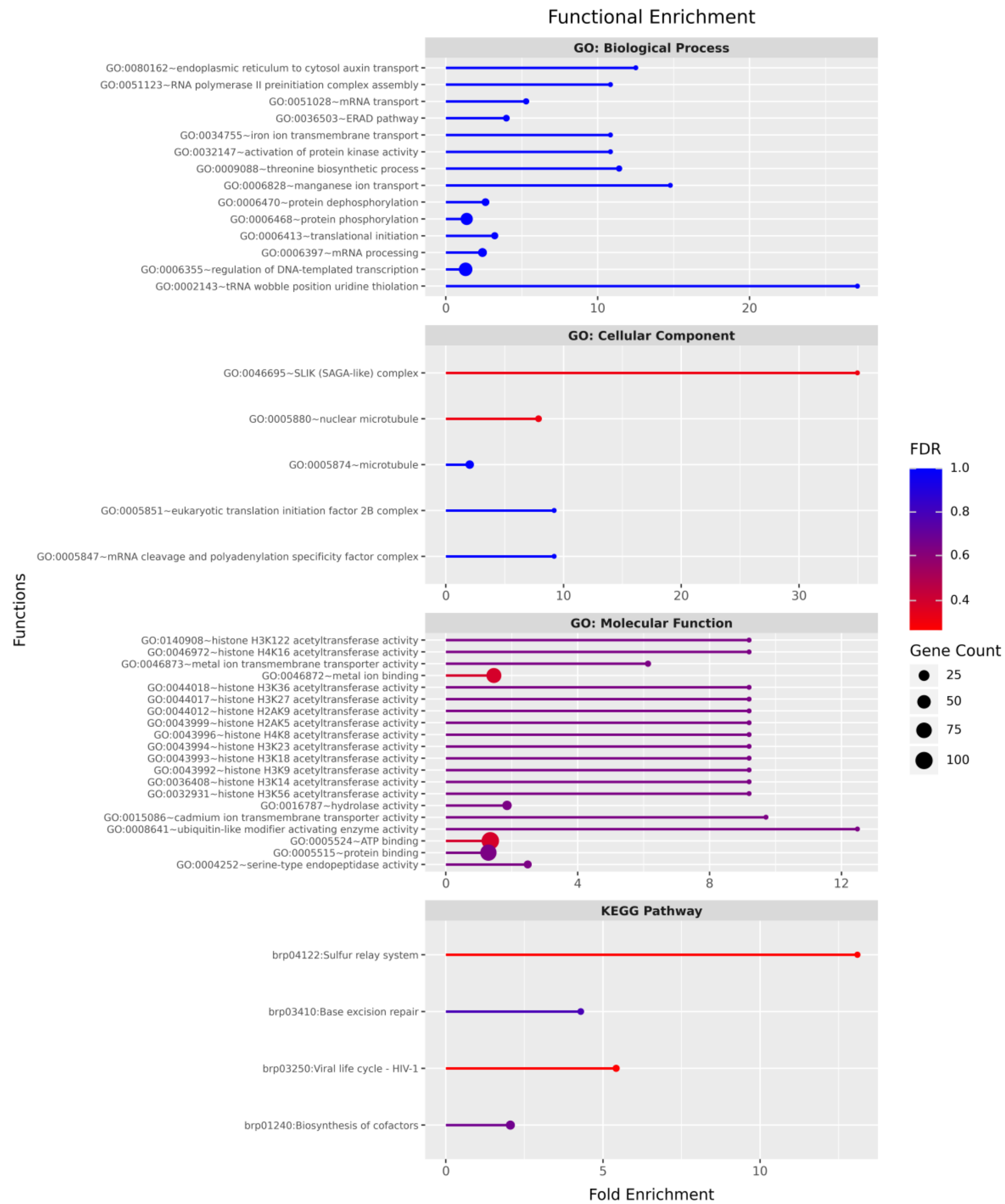
Functional Enrichment
GO: Biological Process
GO:0080162~endoplasmic reticulum to cytosol auxin transport
GO:0051123~RNA polymerase II preinitiation complex assembly
GO:0051028~mRNA transport
GO:0036503~ERAD pathway
GO:0034755~iron ion transmembrane transport
GO:0032147~activation of protein kinase activity
GO:0009088~threonine biosynthetic process
GO:0006828~manganese ion transport
GO:0006470~protein dephosphorylation
GO:0006468~protein phosphorylation
GO:0006413~translational initiation
GO:0006397~mRNA processing
GO:0006355~regulation of DNA-templated transcription
GO:0002143~tRNA wobble position uridine thiolation
0
10
20
GO: Cellular Component
GO:0046695~SLIK (SAGA-like) complex
GO:0005880~nuclear microtubule
GO:0005874~microtubule
GO:0005851~eukaryotic translation initiation factor 2B complex
GO:0005847~mRNA cleavage and polyadenylation specificity factor complex
0
10
20
30
GO: Molecular Function
GO:0140908~histone H3K122 acetyltransferase activity
GO:0046972~histone H4K16 acetyltransferase activity
GO:0046873~metal ion transmembrane transporter activity
GO:0046872~metal ion binding
GO:0044018~histone H3K36 acetyltransferase activity
GO:0044017~histone H3K27 acetyltransferase activity
GO:0044012~histone H2AK9 acetyltransferase activity
GO:0043999~histone H2AK5 acetyltransferase activity
GO:0043996~histone H4K8 acetyltransferase activity
GO:0043994~histone H3K23 acetyltransferase activity
GO:0043993~histone H3K18 acetyltransferase activity
GO:0043992~histone H3K9 acetyltransferase activity
GO:0036408~histone H3K14 acetyltransferase activity
GO:0032931~histone H3K56 acetyltransferase activity
GO:0016787~hydrolase activity
GO:0015086~cadmium ion transmembrane transporter activity
GO:0008641~ubiquitin-like modifier activating enzyme activity
GO:0005524~ATP binding
GO:0005515~protein binding
GO:0004252~serine-type endopeptidase activity
0
4
8
12
KEGG Pathway
brp04122:Sulfur relay system
brp03410:Base excision repair
brp03250:Viral life cycle - HIV-1
brp01240:Biosynthesis of cofactors
0
5
10
Fold Enrichment
Functions
FDR
1.0
0.8
0.6
0.4
Gene Count
25
50
75
100

Figure. S7b

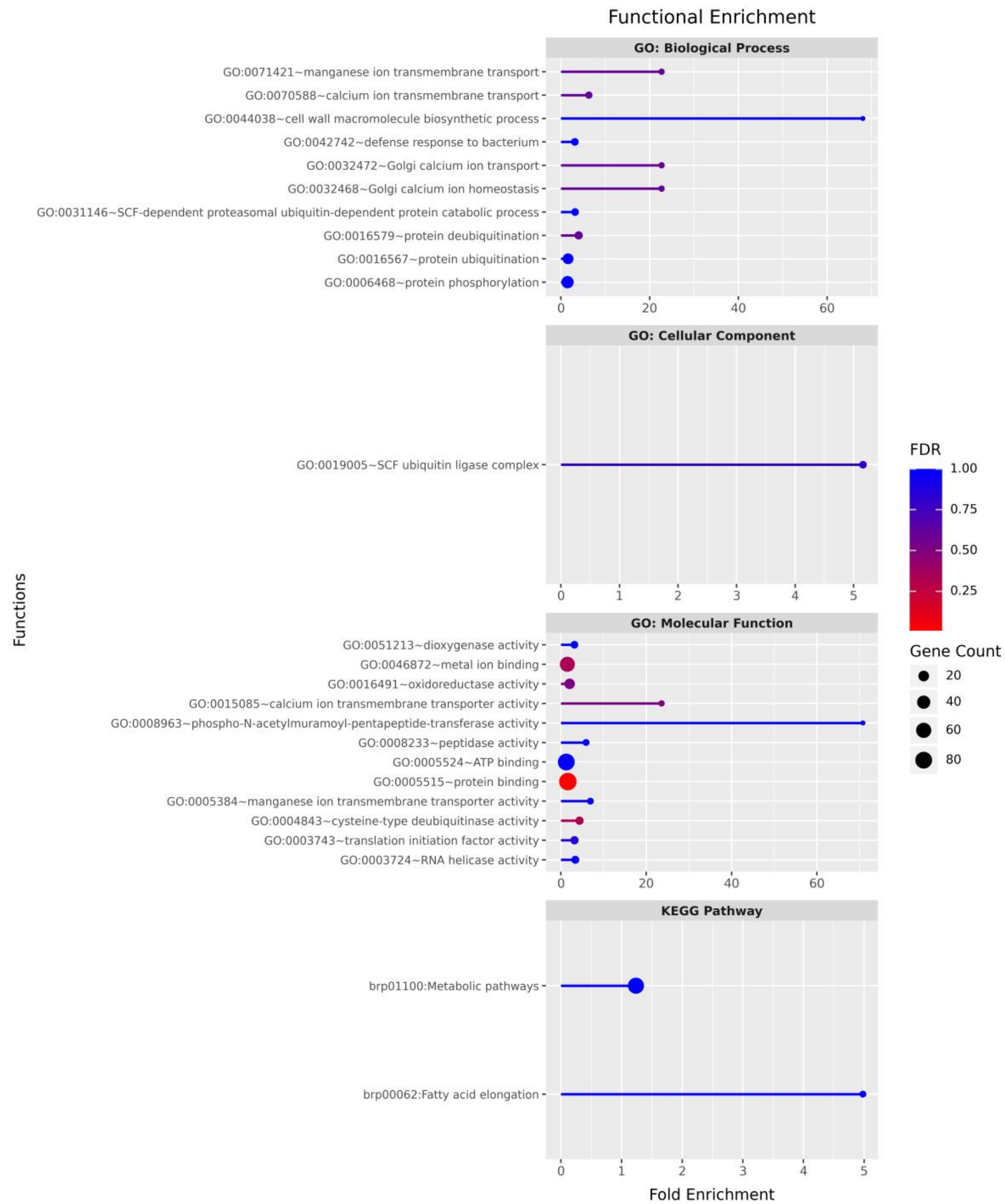

Functional Enrichment
GO: Biological Process
GO:0071421~manganese ion transmembrane transport
GO:0070588~calcium ion transmembrane transport
GO:0044038~cell wall macromolecule biosynthetic process
GO:0042742~defense response to bacterium
GO:0032472~Golgi calcium ion transport
GO:0032468~Golgi calcium ion homeostasis
GO:0031146~SCF-dependent proteasomal ubiquitin-dependent protein catabolic process
GO:0016579~protein deubiquitination
GO:0016567~protein ubiquitination
GO:0006468~protein phosphorylation
0
20
40
60
GO: Cellular Component
GO:0019005~SCF ubiquitin ligase complex
0
1
2
3
4
5
GO: Molecular Function
GO:0051213~dioxygenase activity
GO:0046872~metal ion binding
GO:0016491~oxidoreductase activity
GO:0015085~calcium ion transmembrane transporter activity
GO:0008963~phospho-N-acetylmuramoyl-pentapeptide-transferase activity
GO:0008233~peptidase activity
GO:0005524~ATP binding
GO:0005515~protein binding
GO:0005384~manganese ion transmembrane transporter activity
GO:0004843~cysteine-type deubiquitinase activity
GO:0003743~translation initiation factor activity
GO:0003724~RNA helicase activity
0
20
40
60
KEGG Pathway
brp01100:Metabolic pathways
brp00062:Fatty acid elongation
0
1
2
3
4
5
Fold Enrichment
Functions
FDR
1.00
0.75
0.50
0.25
Gene Count
20
40
60
80

Figure. S8a

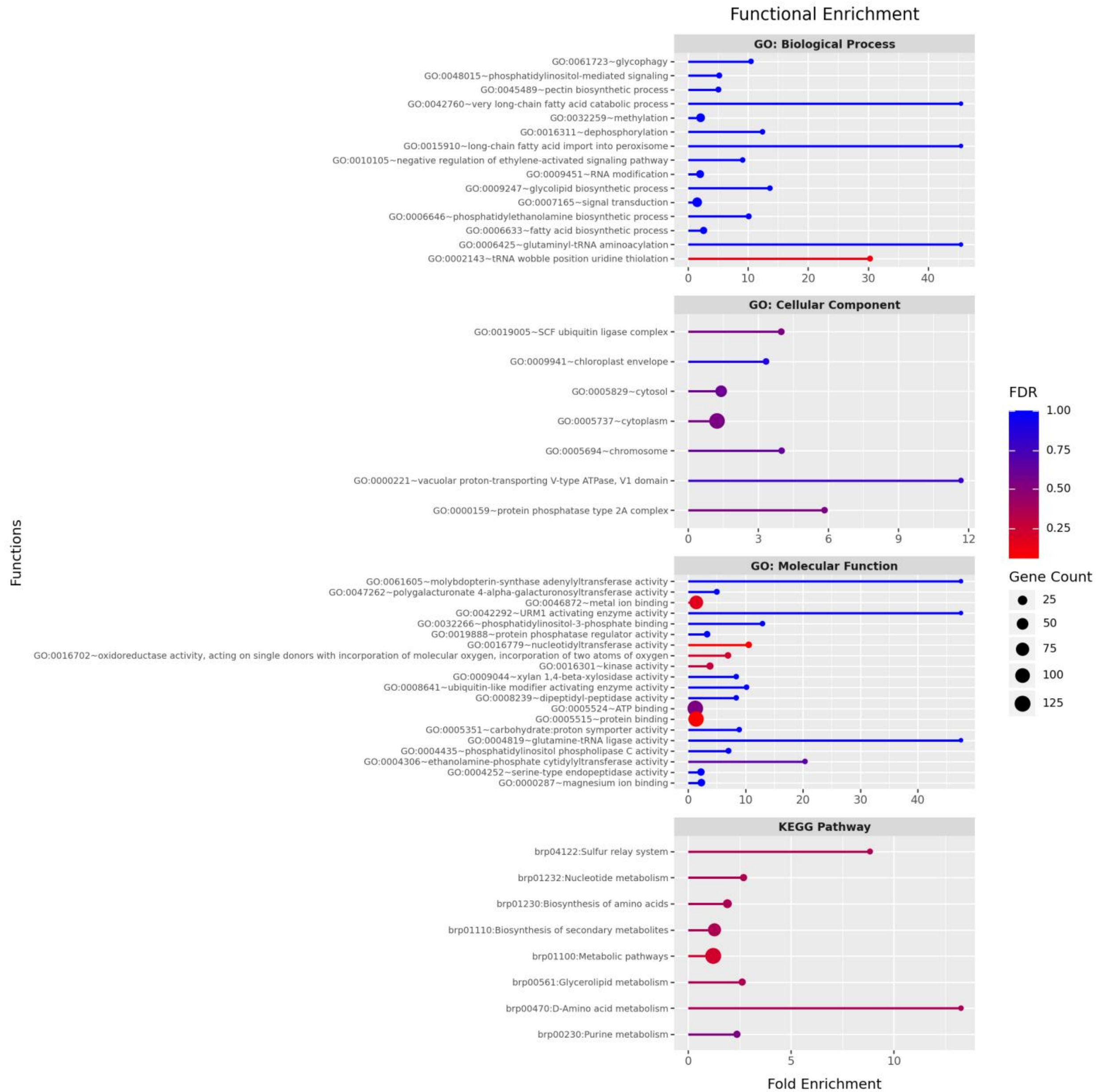
Functional Enrichment
GO: Biological Process
GO:0061723~glycophagy
GO:0048015~phosphatidylinositol-mediated signaling
GO:0045489~pectin biosynthetic process
GO:0042760~very long-chain fatty acid catabolic process
GO:0032259~methylation
GO:0016311~dephosphorylation
GO:0015910~long-chain fatty acid import into peroxisome
GO:0010105~negative regulation of ethylene-activated signaling pathway
GO:0009451~RNA modification
GO:0009247~glycolipid biosynthetic process
GO:0007165~signal transduction
GO:0006646~phosphatidylethanolamine biosynthetic process
GO:0006633~fatty acid biosynthetic process
GO:0006425~glutaminyl-tRNA aminoacylation
GO:0002143~tRNA wobble position uridine thiolation
0 10 20 30 40
GO: Cellular Component
GO:0019005~SCF ubiquitin ligase complex
GO:0009941~chloroplast envelope
GO:0005829~cytosol
GO:0005737~cytoplasm
GO:0005694~chromosome
GO:0000221~vacuolar proton-transporting V-type ATPase, V1 domain
GO:0000159~protein phosphatase type 2A complex
0 3 6 9 12
GO: Molecular Function
GO:0061605~molybdopterin-synthase adenylyltransferase activity
GO:0047262~polygalacturonate 4-alpha-galacturonosyltransferase activity
GO:0046872~metal ion binding
GO:0042292~URM1 activating enzyme activity
GO:0032266~phosphatidylinositol-3-phosphate binding
GO:0019888~protein phosphatase regulator activity
GO:0016779~nucleotidyltransferase activity
GO:0016702~oxidoreductase activity, acting on single donors with incorporation of molecular oxygen, incorporation of two atoms of oxygen
GO:0016301~kinase activity
GO:0009044~xylan 1,4-beta-xylosidase activity
GO:0008641~ubiquitin-like modifier activating enzyme activity
GO:0008239~dipeptidyl-peptidase activity
GO:0005524~ATP binding
GO:0005515~protein binding
GO:0005351~carbohydrate:proton symporter activity
GO:0004819~glutamine-tRNA ligase activity
GO:0004435~phosphatidylinositol phospholipase C activity
GO:0004306~ethanolamine-phosphate cytidylyltransferase activity
GO:0004252~serine-type endopeptidase activity
GO:0000287~magnesium ion binding
0 10 20 30 40
KEGG Pathway
brp04122:Sulfur relay system
brp01232:Nucleotide metabolism
brp01230:Biosynthesis of amino acids
brp01110:Biosynthesis of secondary metabolites
brp01100:Metabolic pathways
brp00561:Glycerolipid metabolism
brp00470:D-Amino acid metabolism
brp00230:Purine metabolism
0 5 10
Fold Enrichment
Functions
FDR
1.00
0.75
0.50
0.25
Gene Count
25
50
75
100
125

Figure. S8b

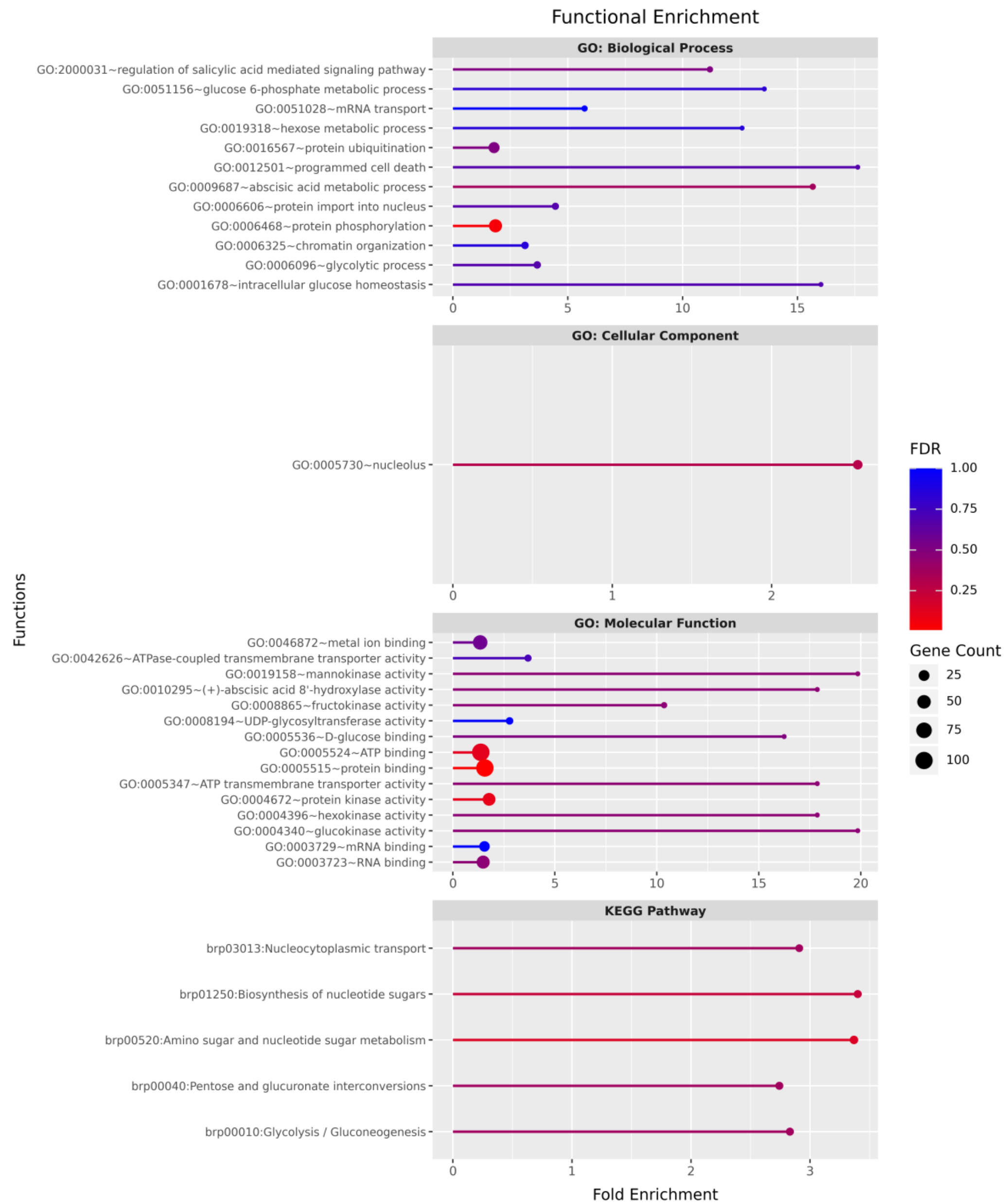
Functional Enrichment
GO: Biological Process
GO:2000031~regulation of salicylic acid mediated signaling pathway
GO:0051156~glucose 6-phosphate metabolic process
GO:0051028~mRNA transport
GO:0019318~hexose metabolic process
GO:0016567~protein ubiquitination
GO:0012501~programmed cell death
GO:0009687~abscisic acid metabolic process
GO:0006606~protein import into nucleus
GO:0006468~protein phosphorylation
GO:0006325~chromatin organization
GO:0006096~glycolytic process
GO:0001678~intracellular glucose homeostasis
0
5
10
15
GO: Cellular Component
GO:0005730~nucleolus
0
1
2
GO: Molecular Function
GO:0046872~metal ion binding
GO:0042626~ATPase-coupled transmembrane transporter activity
GO:0019158~mannokinase activity
GO:0010295~(+)-abscisic acid 8'-hydroxylase activity
GO:0008865~fructokinase activity
GO:0008194~UDP-glycosyltransferase activity
GO:0005536~D-glucose binding
GO:0005524~ATP binding
GO:0005515~protein binding
GO:0005347~ATP transmembrane transporter activity
GO:0004672~protein kinase activity
GO:0004396~hexokinase activity
GO:0004340~glucokinase activity
GO:0003729~mRNA binding
GO:0003723~RNA binding
0
5
10
15
20
KEGG Pathway
brp03013:Nucleocytoplasmic transport
brp01250:Biosynthesis of nucleotide sugars
brp00520:Amino sugar and nucleotide sugar metabolism
brp00040:Pentose and glucuronate interconversions
brp00010:Glycolysis / Gluconeogenesis
0
1
2
3
Fold Enrichment
Functions
FDR
1.00
0.75
0.50
0.25
Gene Count
25
50
75
100

Figure. S9a

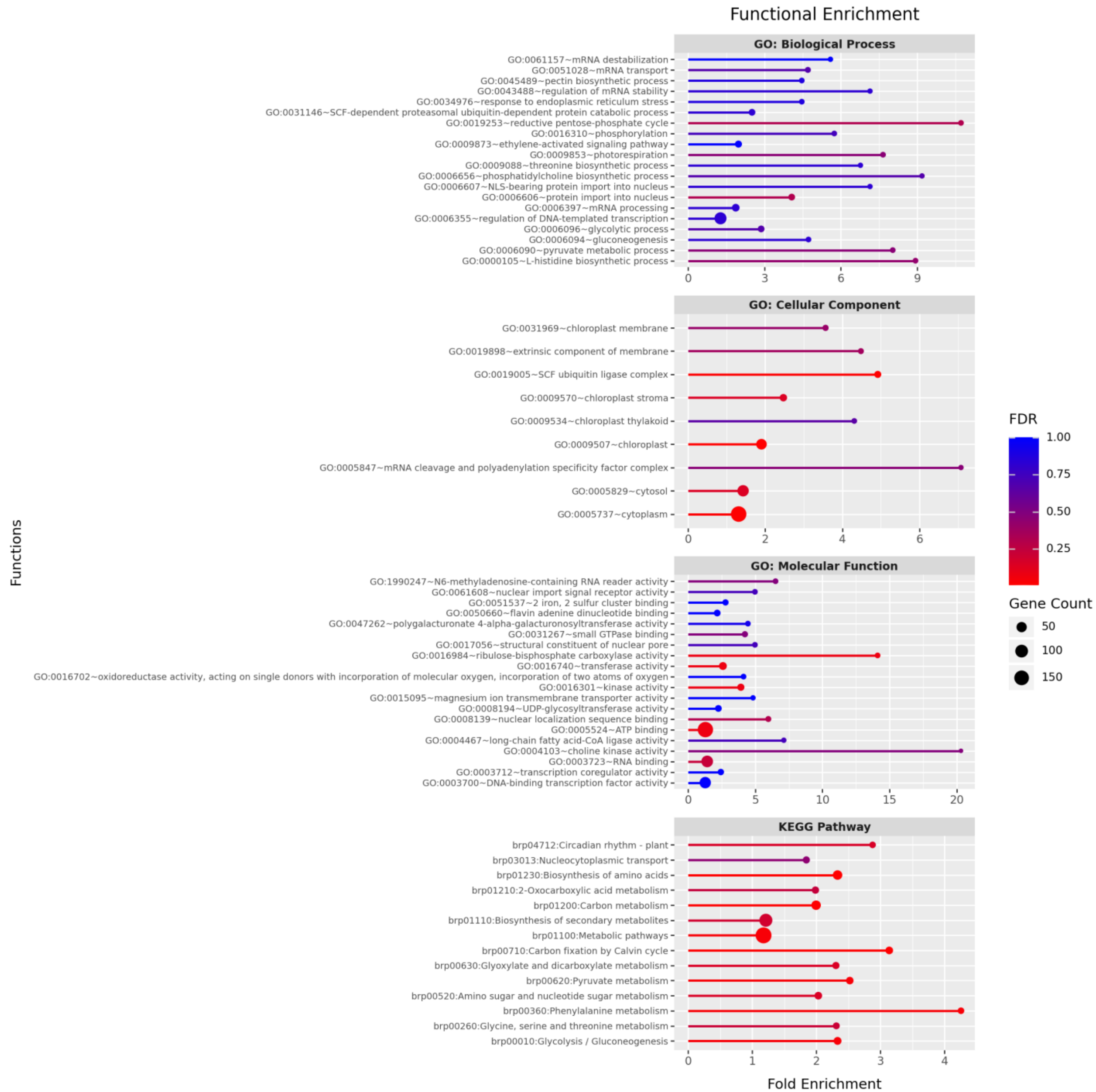
Functional Enrichment
GO: Biological Process
GO:0061157~mRNA destabilization
GO:0051028~mRNA transport
GO:0045489~pectin biosynthetic process
GO:0043488~regulation of mRNA stability
GO:0034976~response to endoplasmic reticulum stress
GO:0031146~SCF-dependent proteasomal ubiquitin-dependent protein catabolic process
GO:0019253~reductive pentose-phosphate cycle
GO:0016310~phosphorylation
GO:0009873~ethylene-activated signaling pathway
GO:0009853~photorespiration
GO:0009088~threonine biosynthetic process
GO:0006656~phosphatidylcholine biosynthetic process
GO:0006607~NLS-bearing protein import into nucleus
GO:0006606~protein import into nucleus
GO:0006397~mRNA processing
GO:0006355~regulation of DNA-templated transcription
GO:0006096~glycolytic process
GO:0006094~gluconeogenesis
GO:0006090~pyruvate metabolic process
GO:0000105~L-histidine biosynthetic process
0
3
6
9
GO: Cellular Component
GO:0031969~chloroplast membrane
GO:0019898~extrinsic component of membrane
GO:0019005~SCF ubiquitin ligase complex
GO:0009570~chloroplast stroma
GO:0009534~chloroplast thylakoid
GO:0009507~chloroplast
GO:0005847~mRNA cleavage and polyadenylation specificity factor complex
GO:0005829~cytosol
GO:0005737~cytoplasm
0
2
4
6
GO: Molecular Function
GO:1990247~N6-methyladenosine-containing RNA reader activity
GO:0061608~nuclear import signal receptor activity
GO:0051537~2 iron, 2 sulfur cluster binding
GO:0050660~flavin adenine dinucleotide binding
GO:0047262~polygalacturonate 4-alpha-galacturonosyltransferase activity
GO:0031267~small GTPase binding
GO:0017056~structural constituent of nuclear pore
GO:0016984~ribulose-bisphosphate carboxylase activity
GO:0016740~transferase activity
GO:0016702~oxidoreductase activity, acting on single donors with incorporation of molecular oxygen, incorporation of two atoms of oxygen
GO:0016301~kinase activity
GO:0015095~magnesium ion transmembrane transporter activity
GO:0008194~UDP-glycosyltransferase activity
GO:0008139~nuclear localization sequence binding
GO:0005524~ATP binding
GO:0004467~long-chain fatty acid-CoA ligase activity
GO:0004103~choline kinase activity
GO:0003723~RNA binding
GO:0003712~transcription coregulator activity
GO:0003700~DNA-binding transcription factor activity
0
5
10
15
20
KEGG Pathway
brp04712:Circadian rhythm - plant
brp03013:Nucleocytoplasmic transport
brp01230:Biosynthesis of amino acids
brp01210:2-Oxocarboxylic acid metabolism
brp01200:Carbon metabolism
brp01110:Biosynthesis of secondary metabolites
brp01100:Metabolic pathways
brp00710:Carbon fixation by Calvin cycle
brp00630:Glyoxylate and dicarboxylate metabolism
brp00620:Pyruvate metabolism
brp00520:Amino sugar and nucleotide sugar metabolism
brp00360:Phenylalanine metabolism
brp00260:Glycine, serine and threonine metabolism
brp00010:Glycolysis / Gluconeogenesis
0
1
2
3
4
Fold Enrichment
Functions
FDR
1.00
0.75
0.50
0.25
Gene Count
50
100
150

Figure. S9b

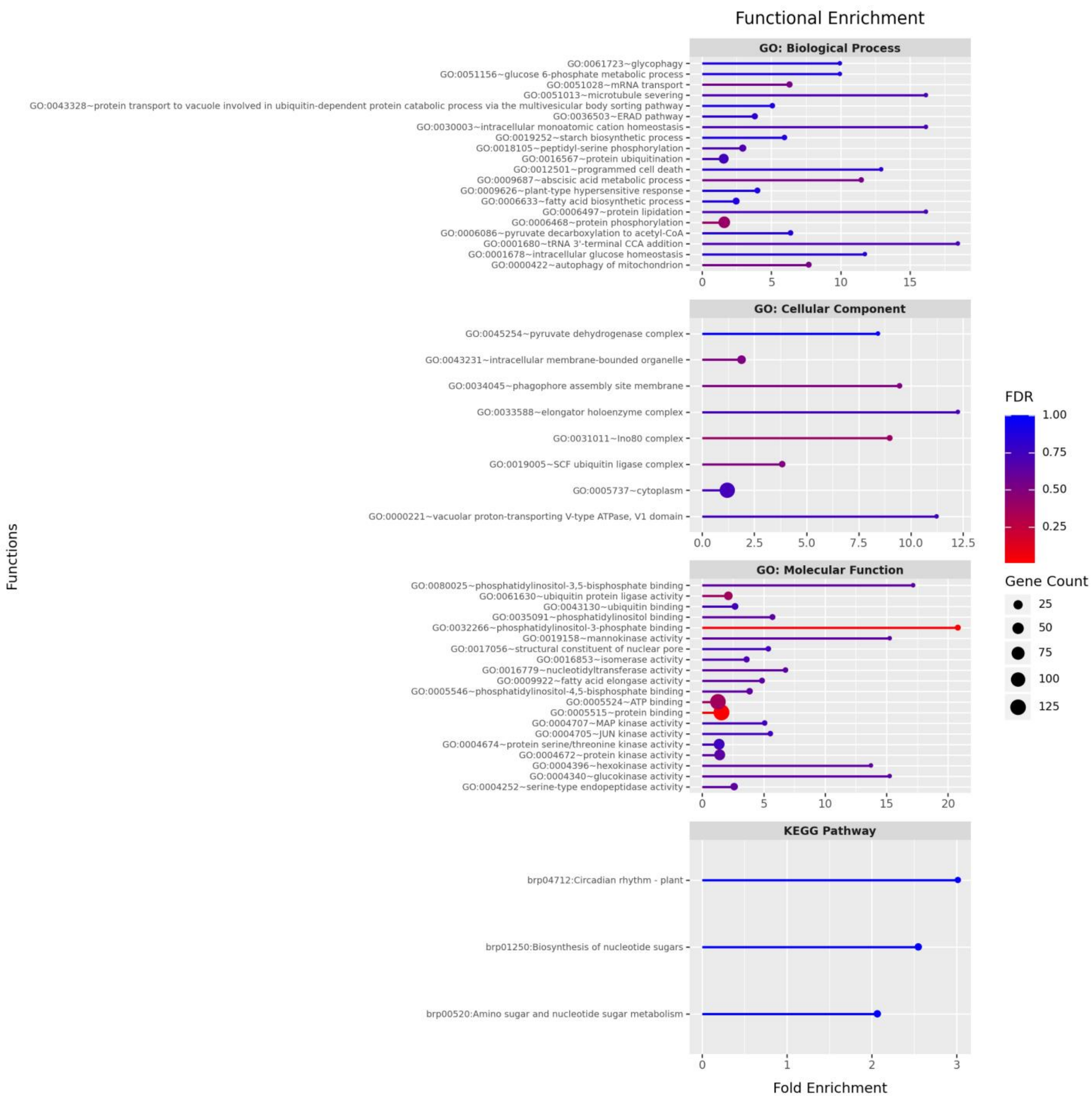
Functional Enrichment
GO: Biological Process
GO:0061723~glycophagy
GO:0051156~glucose 6-phosphate metabolic process
GO:0051028~mRNA transport
GO:0051013~microtubule severing
GO:0043328~protein transport to vacuole involved in ubiquitin-dependent protein catabolic process via the multivesicular body sorting pathway
GO:0036503~ERAD pathway
GO:0030003~intracellular monoatomic cation homeostasis
GO:0019252~starch biosynthetic process
GO:0018105~peptidyl-serine phosphorylation
GO:0016567~protein ubiquitination
GO:0012501~programmed cell death
GO:0009687~abscisic acid metabolic process
GO:0009626~plant-type hypersensitive response
GO:0006633~fatty acid biosynthetic process
GO:0006497~protein lipidation
GO:0006468~protein phosphorylation
GO:0006086~pyruvate decarboxylation to acetyl-CoA
GO:0001680~tRNA 3'-terminal CCA addition
GO:0001678~intracellular glucose homeostasis
GO:0000422~autophagy of mitochondrion
0 5 10 15
GO: Cellular Component
GO:0045254~pyruvate dehydrogenase complex
GO:0043231~intracellular membrane-bounded organelle
GO:0034045~phagophore assembly site membrane
GO:0033588~elongator holoenzyme complex
GO:0031011~Ino80 complex
GO:0019005~SCF ubiquitin ligase complex
GO:0005737~cytoplasm
GO:0000221~vacuolar proton-transporting V-type ATPase, V1 domain
0.0 2.5 5.0 7.5 10.0 12.5
GO: Molecular Function
GO:0080025~phosphatidylinositol-3,5-bisphosphate binding
GO:0061630~ubiquitin protein ligase activity
GO:0043130~ubiquitin binding
GO:0035091~phosphatidylinositol binding
GO:0032266~phosphatidylinositol-3-phosphate binding
GO:0019158~mannokinase activity
GO:0017056~structural constituent of nuclear pore
GO:0016853~isomerase activity
GO:0016779~nucleotidyltransferase activity
GO:0009922~fatty acid elongase activity
GO:0005546~phosphatidylinositol-4,5-bisphosphate binding
GO:0005524~ATP binding
GO:0005515~protein binding
GO:0004707~MAP kinase activity
GO:0004705~JUN kinase activity
GO:0004674~protein serine/threonine kinase activity
GO:0004672~protein kinase activity
GO:0004396~hexokinase activity
GO:0004340~glucokinase activity
GO:0004252~serine-type endopeptidase activity
0 5 10 15 20
KEGG Pathway
brp04712:Circadian rhythm - plant
brp01250:Biosynthesis of nucleotide sugars
brp00520:Amino sugar and nucleotide sugar metabolism
0 1 2 3
Fold Enrichment
Functions
FDR
1.00
0.75
0.50
0.25
Gene Count
25
50
75
100
125

Figure. S10a

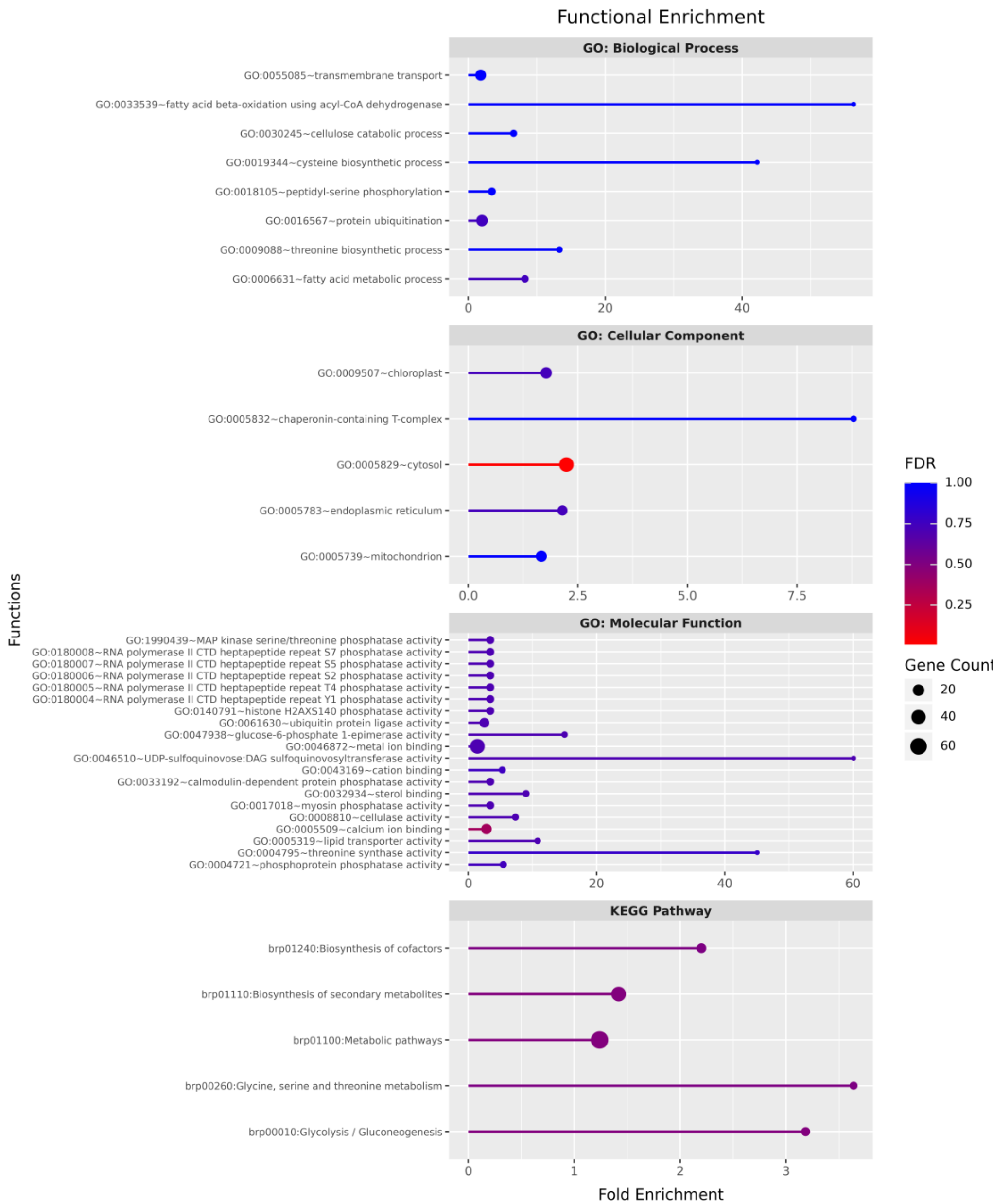

Functional Enrichment
GO: Biological Process
GO:0055085~transmembrane transport
GO:0033539~fatty acid beta-oxidation using acyl-CoA dehydrogenase
GO:0030245~cellulose catabolic process
GO:0019344~cysteine biosynthetic process
GO:0018105~peptidyl-serine phosphorylation
GO:0016567~protein ubiquitination
GO:0009088~threonine biosynthetic process
GO:0006631~fatty acid metabolic process
0
20
40
GO: Cellular Component
GO:0009507~chloroplast
GO:0005832~chaperonin-containing T-complex
GO:0005829~cytosol
GO:0005783~endoplasmic reticulum
GO:0005739~mitochondrion
0.0
2.5
5.0
7.5
GO: Molecular Function
GO:1990439~MAP kinase serine/threonine phosphatase activity
GO:0180008~RNA polymerase II CTD heptapeptide repeat S7 phosphatase activity
GO:0180007~RNA polymerase II CTD heptapeptide repeat S5 phosphatase activity
GO:0180006~RNA polymerase II CTD heptapeptide repeat S2 phosphatase activity
GO:0180005~RNA polymerase II CTD heptapeptide repeat T4 phosphatase activity
GO:0180004~RNA polymerase II CTD heptapeptide repeat Y1 phosphatase activity
GO:0140791~histone H2AXS140 phosphatase activity
GO:0061630~ubiquitin protein ligase activity
GO:0047938~glucose-6-phosphate 1-epimerase activity
GO:0046872~metal ion binding
GO:0046510~UDP-sulfoquinovose:DAG sulfoquinovosyltransferase activity
GO:0043169~cation binding
GO:0033192~calmodulin-dependent protein phosphatase activity
GO:0032934~sterol binding
GO:0017018~myosin phosphatase activity
GO:0008810~cellulase activity
GO:0005509~calcium ion binding
GO:0005319~lipid transporter activity
GO:0004795~threonine synthase activity
GO:0004721~phosphoprotein phosphatase activity
0
20
40
60
KEGG Pathway
brp01240:Biosynthesis of cofactors
brp01110:Biosynthesis of secondary metabolites
brp01100:Metabolic pathways
brp00260:Glycine, serine and threonine metabolism
brp00010:Glycolysis / Gluconeogenesis
0
1
2
3
Fold Enrichment
Functions
FDR
1.00
0.75
0.50
0.25
Gene Count
20
40
60

Figure. S10b

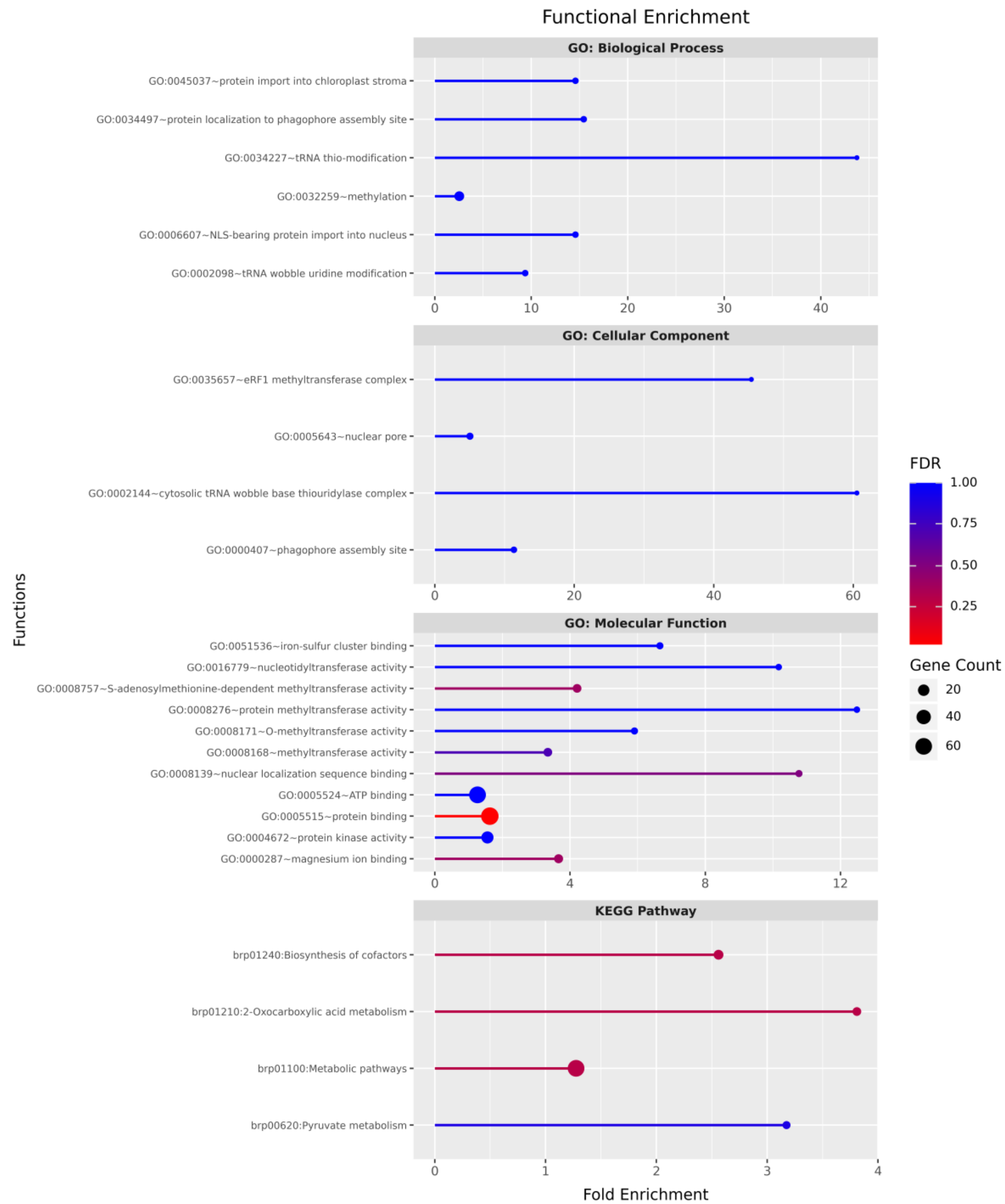

Functional Enrichment
GO: Biological Process
GO:0045037~protein import into chloroplast stroma
GO:0034497~protein localization to phagophore assembly site
GO:0034227~tRNA thio-modification
GO:0032259~methylation
GO:0006607~NLS-bearing protein import into nucleus
GO:0002098~tRNA wobble uridine modification
0 10 20 30 40
GO: Cellular Component
GO:0035657~eRF1 methyltransferase complex
GO:0005643~nuclear pore
GO:0002144~cytosolic tRNA wobble base thiouridylase complex
GO:0000407~phagophore assembly site
0 20 40 60
GO: Molecular Function
GO:0051536~iron-sulfur cluster binding
GO:0016779~nucleotidyltransferase activity
GO:0008757~S-adenosylmethionine-dependent methyltransferase activity
GO:0008276~protein methyltransferase activity
GO:0008171~O-methyltransferase activity
GO:0008168~methyltransferase activity
GO:0008139~nuclear localization sequence binding
GO:0005524~ATP binding
GO:0005515~protein binding
GO:0004672~protein kinase activity
GO:0000287~magnesium ion binding
0 4 8 12
KEGG Pathway
brp01240:Biosynthesis of cofactors
brp01210:2-Oxocarboxylic acid metabolism
brp01100:Metabolic pathways
brp00620:Pyruvate metabolism
0 1 2 3 4
Functions
Fold Enrichment
FDR
1.00
0.75
0.50
0.25
Gene Count
20
40
60

Figure. S11a

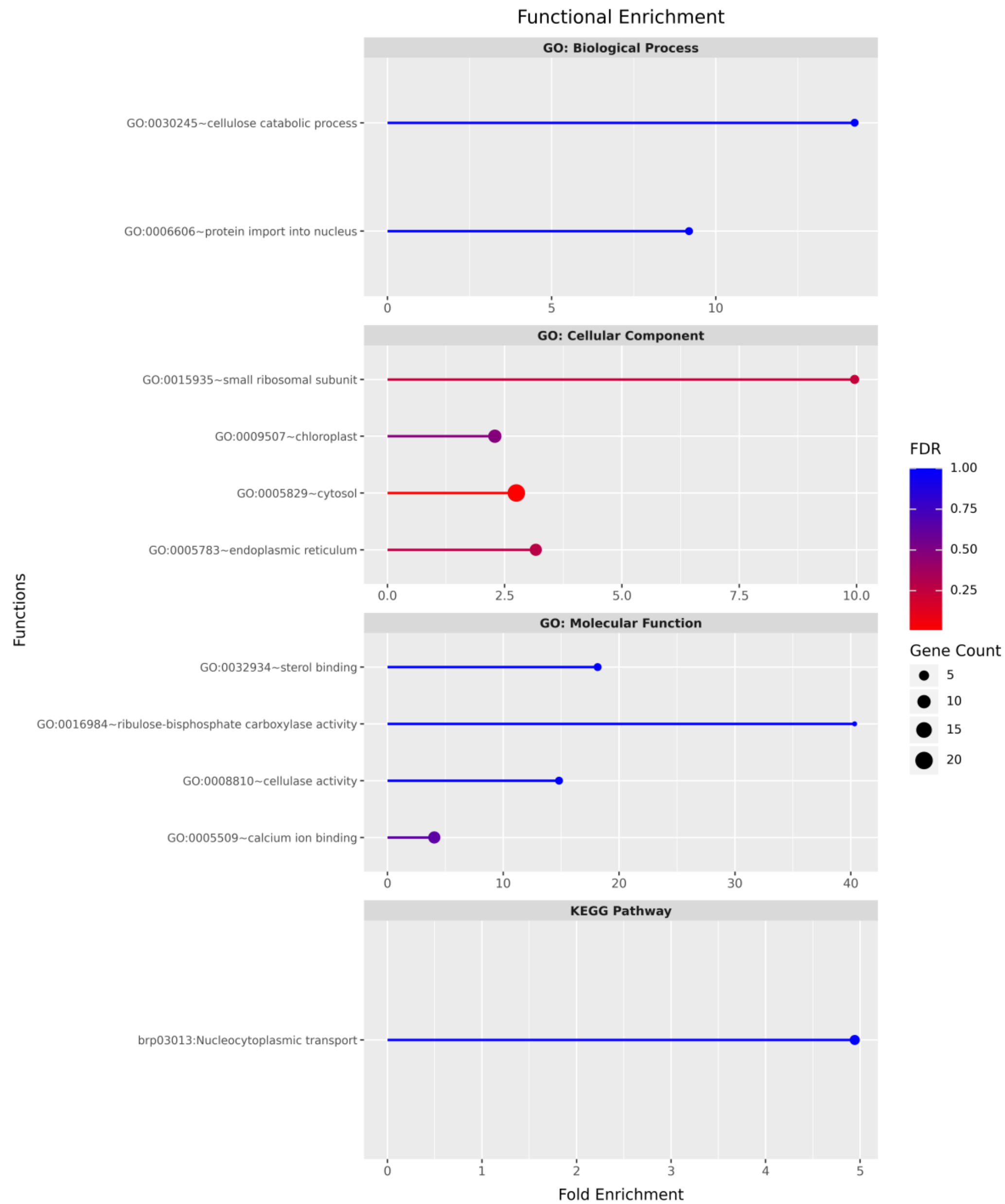

Functional Enrichment
GO: Biological Process
GO:0030245~cellulose catabolic process
GO:0006606~protein import into nucleus
0
5
10
GO: Cellular Component
GO:0015935~small ribosomal subunit
GO:0009507~chloroplast
GO:0005829~cytosol
GO:0005783~endoplasmic reticulum
0.0
2.5
5.0
7.5
10.0
GO: Molecular Function
GO:0032934~sterol binding
GO:0016984~ribulose-bisphosphate carboxylase activity
GO:0008810~cellulase activity
GO:0005509~calcium ion binding
0
10
20
30
40
KEGG Pathway
brp03013:Nucleocytoplasmic transport
0
1
2
3
4
5
Fold Enrichment
Functions
FDR
1.00
0.75
0.50
0.25
Gene Count
5
10
15
20

Figure. S12a

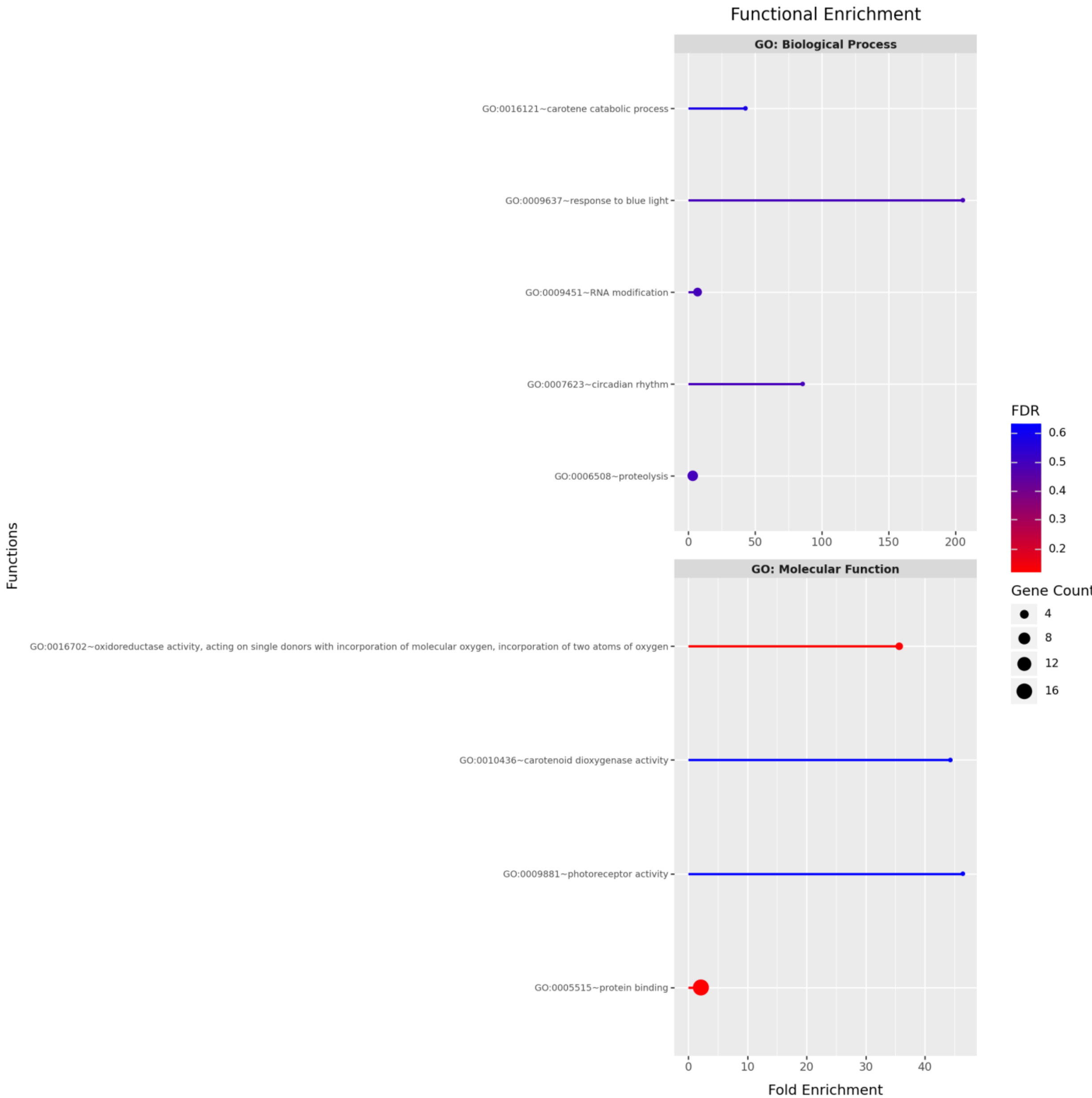

Functional Enrichment
GO: Biological Process
GO:0016121~carotene catabolic process
GO:0009637~response to blue light
GO:0009451~RNA modification
GO:0007623~circadian rhythm
GO:0006508~proteolysis
0
50
100
150
200
GO: Molecular Function
GO:0016702~oxidoreductase activity, acting on single donors with incorporation of molecular oxygen, incorporation of two atoms of oxygen
GO:0010436~carotenoid dioxygenase activity
GO:0009881~photoreceptor activity
GO:0005515~protein binding
0
10
20
30
40
Fold Enrichment
Functions
FDR
0.6
0.5
0.4
0.3
0.2
Gene Count
4
8
12
16

Figure. S12b

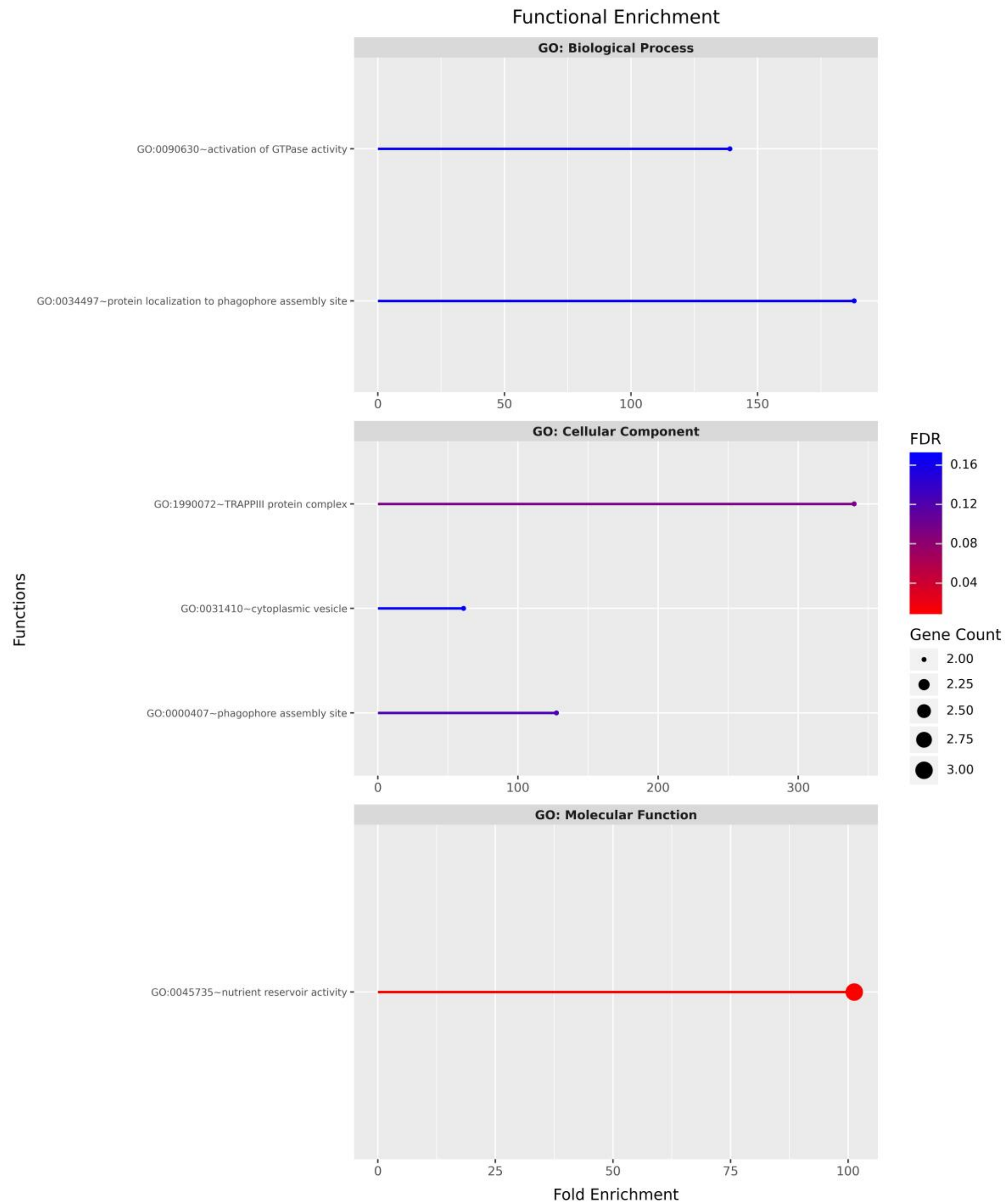

Functional Enrichment
GO: Biological Process
GO:0090630~activation of GTPase activity
GO:0034497~protein localization to phagophore assembly site
0
50
100
150
GO: Cellular Component
GO:1990072~TRAPPIII protein complex
GO:0031410~cytoplasmic vesicle
GO:0000407~phagophore assembly site
0
100
200
300
GO: Molecular Function
GO:0045735~nutrient reservoir activity
0
25
50
75
100
Fold Enrichment
Functions
FDR
0.16
0.12
0.08
0.04
Gene Count
2.00
2.25
2.50
2.75
3.00

Figure. S13a

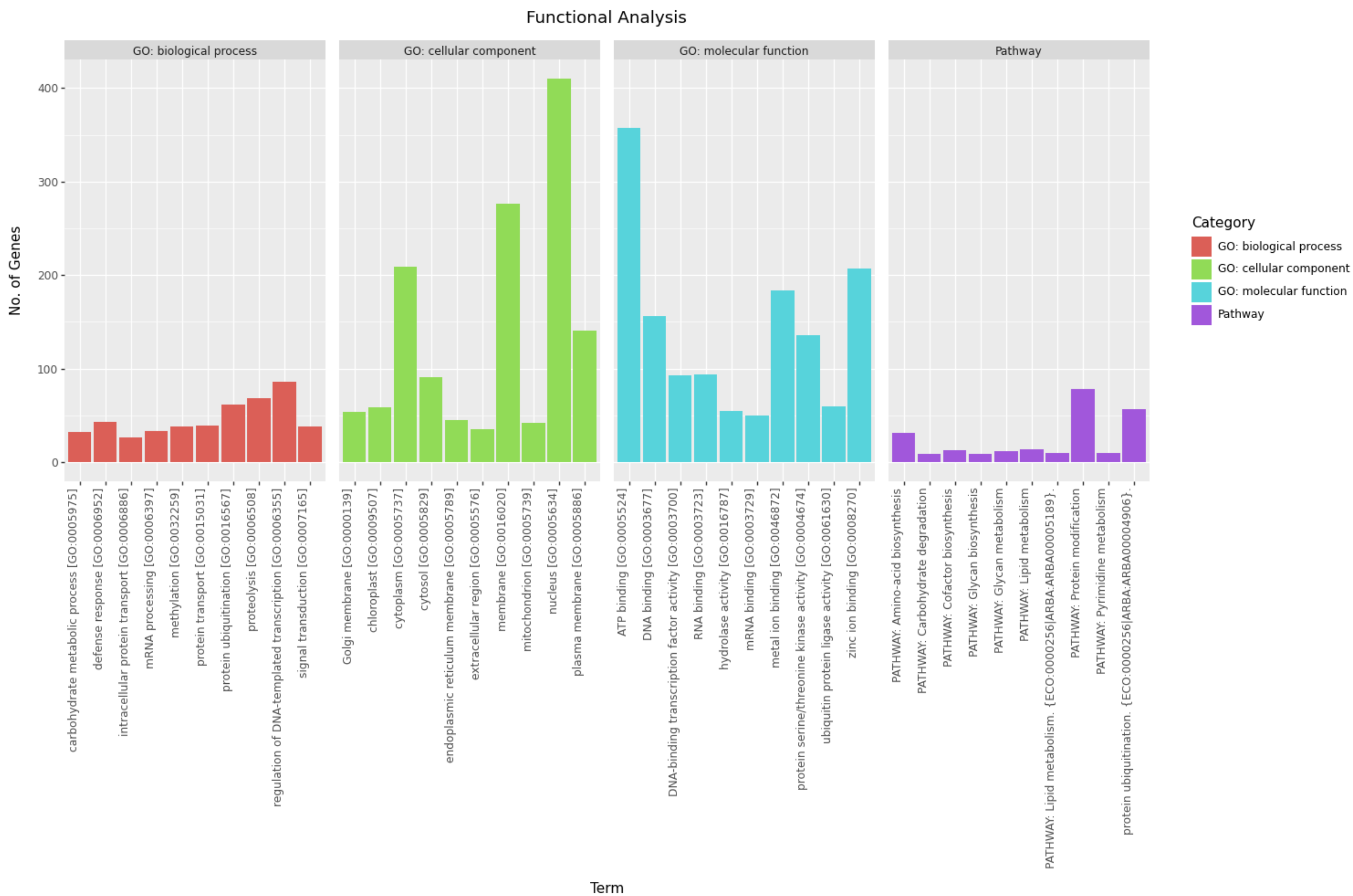
Functional Analysis
GO: biological process
GO: cellular component
GO: molecular function
Pathway
No. of Genes
0
100
200
300
400
carbohydrate metabolic process [GO:0005975]
defense response [GO:0006952]
intracellular protein transport [GO:0006886]
mRNA processing [GO:0006397]
methylation [GO:0032259]
protein transport [GO:0015031]
protein ubiquitination [GO:0016567]
proteolysis [GO:0006508]
regulation of DNA-templated transcription [GO:0006355]
signal transduction [GO:0007165]
Golgi membrane [GO:0000139]
chloroplast [GO:0009507]
cytoplasm [GO:0005737]
cytosol [GO:0005829]
endoplasmic reticulum membrane [GO:0005789]
extracellular region [GO:0005576]
membrane [GO:0016020]
mitochondrion [GO:0005739]
nucleus [GO:0005634]
plasma membrane [GO:0005886]
ATP binding [GO:0005524]
DNA binding [GO:0003677]
DNA-binding transcription factor activity [GO:0003700]
RNA binding [GO:0003723]
hydrolase activity [GO:0016787]
mRNA binding [GO:0003729]
metal ion binding [GO:0046872]
protein serine/threonine kinase activity [GO:0004674]
ubiquitin protein ligase activity [GO:0061630]
zinc ion binding [GO:0008270]
PATHWAY: Amino-acid biosynthesis
PATHWAY: Carbohydrate degradation
PATHWAY: Cofactor biosynthesis
PATHWAY: Glycan biosynthesis
PATHWAY: Glycan metabolism
PATHWAY: Lipid metabolism
PATHWAY: Lipid metabolism. {ECO:0000256|ARBA:ARBA00005189}.
PATHWAY: Protein modification
PATHWAY: Pyrimidine metabolism
protein ubiquitination. {ECO:0000256|ARBA:ARBA00004906}.
Term
Category
GO: biological process
GO: cellular component
GO: molecular function
Pathway

Figure. S13b

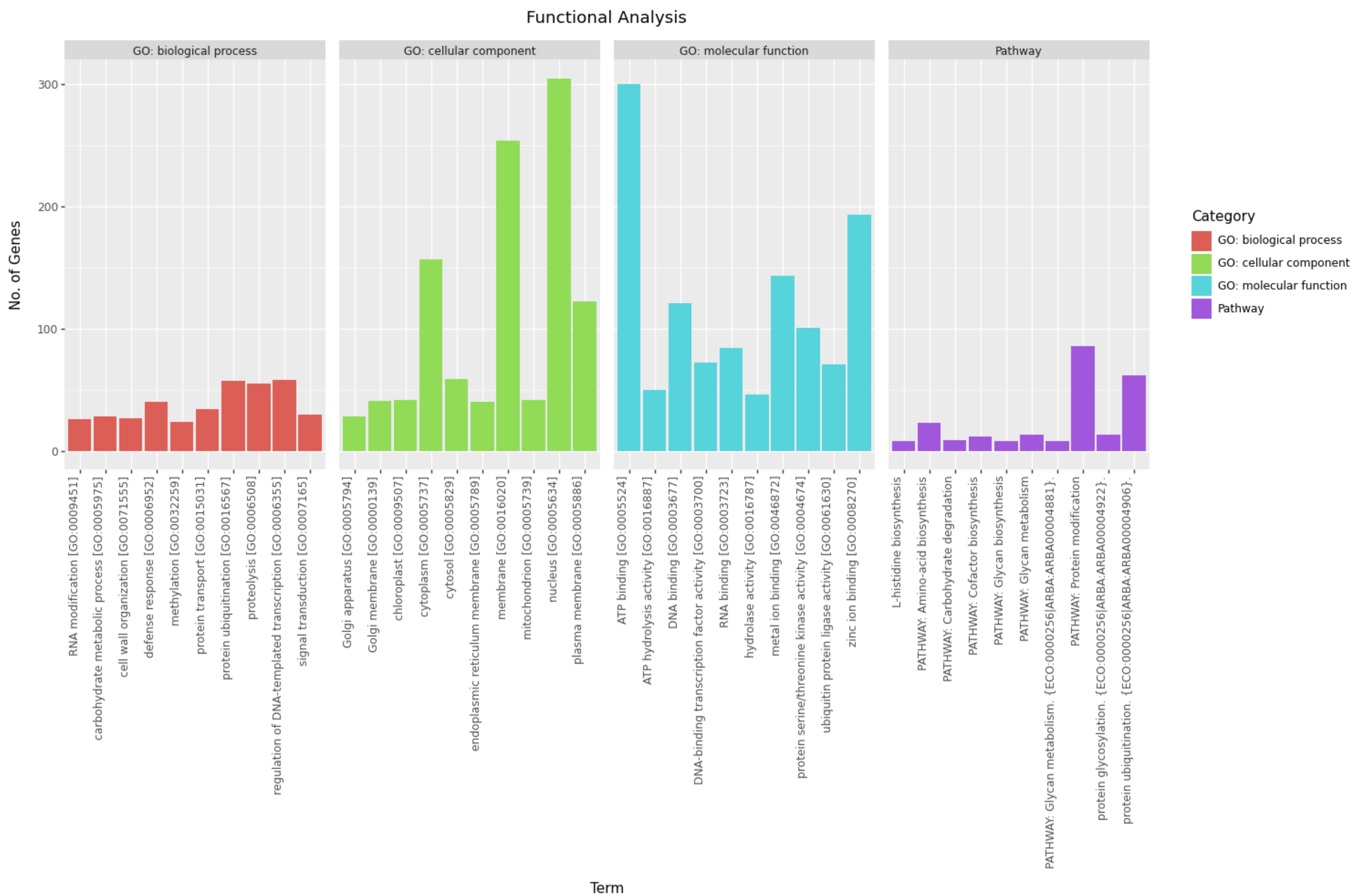
Functional Analysis
GO: biological process
GO: cellular component
GO: molecular function
Pathway
No. of Genes
0
100
200
300
RNA modification [GO:0009451]
carbohydrate metabolic process [GO:0005975]
cell wall organization [GO:0071555]
defense response [GO:0006952]
methylation [GO:0032259]
protein transport [GO:0015031]
protein ubiquitination [GO:0016567]
proteolysis [GO:0006508]
regulation of DNA-templated transcription [GO:0006355]
signal transduction [GO:0007165]
Golgi apparatus [GO:0005794]
Golgi membrane [GO:0000139]
chloroplast [GO:0009507]
cytoplasm [GO:0005737]
cytosol [GO:0005829]
endoplasmic reticulum membrane [GO:0005789]
membrane [GO:0016020]
mitochondrion [GO:0005739]
nucleus [GO:0005634]
plasma membrane [GO:0005886]
ATP binding [GO:0005524]
ATP hydrolysis activity [GO:0016887]
DNA binding [GO:0003677]
DNA-binding transcription factor activity [GO:0003700]
RNA binding [GO:0003723]
hydrolase activity [GO:0016787]
metal ion binding [GO:0046872]
protein serine/threonine kinase activity [GO:0004674]
ubiquitin protein ligase activity [GO:0061630]
zinc ion binding [GO:0008270]
L-histidine biosynthesis
PATHWAY: Amino-acid biosynthesis
PATHWAY: Carbohydrate degradation
PATHWAY: Cofactor biosynthesis
PATHWAY: Glycan biosynthesis
PATHWAY: Glycan metabolism
PATHWAY: Glycan metabolism. {ECO:0000256|ARBA:ARBA00004881}.
PATHWAY: Protein modification
protein glycosylation. {ECO:0000256|ARBA:ARBA00004922}.
protein ubiquitination. {ECO:0000256|ARBA:ARBA00004906}.
Term
Category
GO: biological process
GO: cellular component
GO: molecular function
Pathway

Figure. S14a

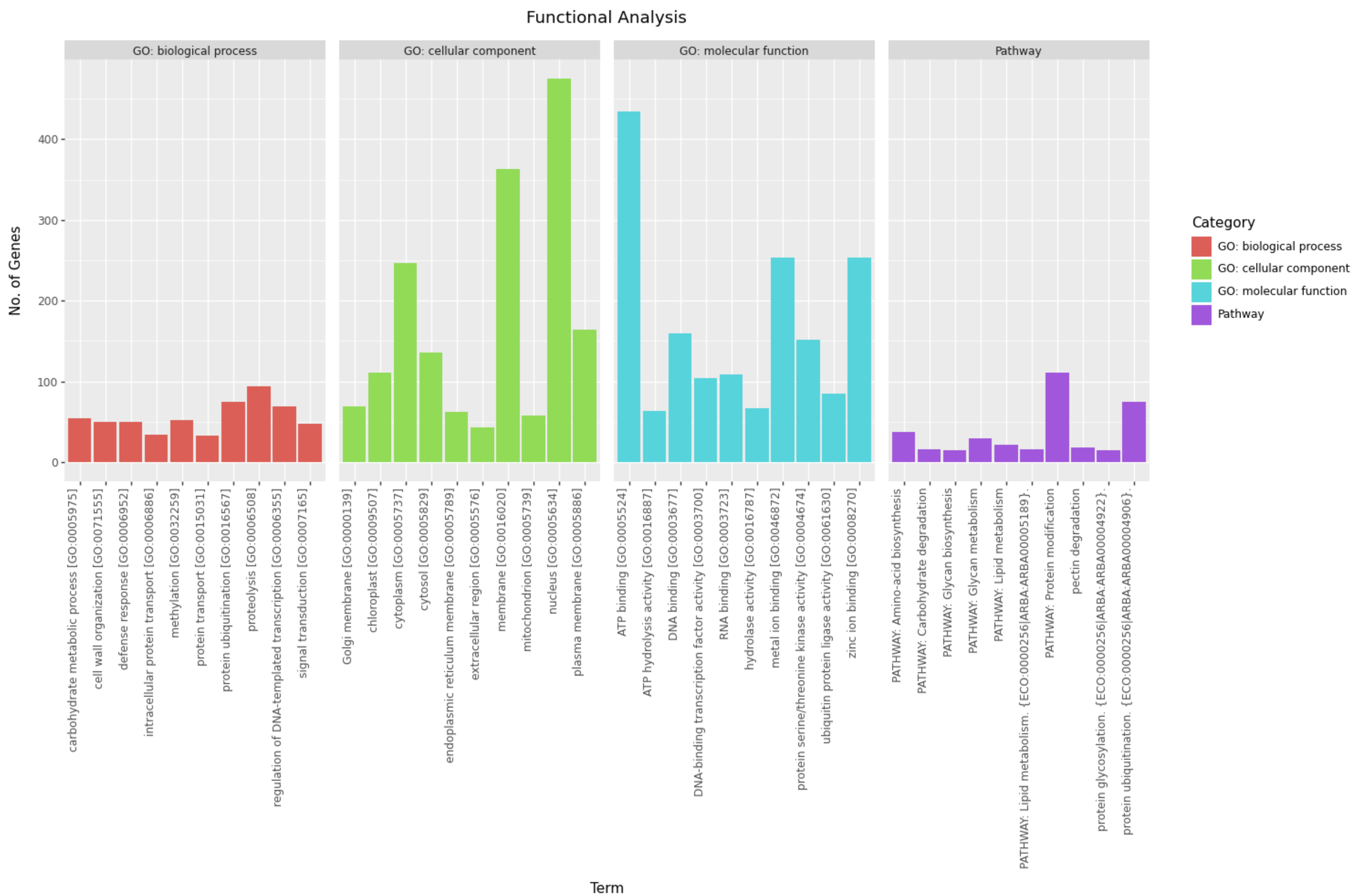
Functional Analysis
GO: biological process
GO: cellular component
GO: molecular function
Pathway
No. of Genes
0
100
200
300
400
carbohydrate metabolic process [GO:0005975]
cell wall organization [GO:0071555]
defense response [GO:0006952]
intracellular protein transport [GO:0006886]
methylation [GO:0032259]
protein transport [GO:0015031]
protein ubiquitination [GO:0016567]
proteolysis [GO:0006508]
regulation of DNA-templated transcription [GO:0006355]
signal transduction [GO:0007165]
Golgi membrane [GO:0000139]
chloroplast [GO:0009507]
cytoplasm [GO:0005737]
cytosol [GO:0005829]
endoplasmic reticulum membrane [GO:0005789]
extracellular region [GO:0005576]
membrane [GO:0016020]
mitochondrion [GO:0005739]
nucleus [GO:0005634]
plasma membrane [GO:0005886]
ATP binding [GO:0005524]
ATP hydrolysis activity [GO:0016887]
DNA binding [GO:0003677]
DNA-binding transcription factor activity [GO:0003700]
RNA binding [GO:0003723]
hydrolase activity [GO:0016787]
metal ion binding [GO:0046872]
protein serine/threonine kinase activity [GO:0004674]
ubiquitin protein ligase activity [GO:0061630]
zinc ion binding [GO:0008270]
PATHWAY: Amino-acid biosynthesis
PATHWAY: Carbohydrate degradation
PATHWAY: Glycan biosynthesis
PATHWAY: Glycan metabolism
PATHWAY: Lipid metabolism
PATHWAY: Lipid metabolism. {ECO:0000256|ARBA:ARBA00005189}.
PATHWAY: Protein modification
pectin degradation
protein glycosylation. {ECO:0000256|ARBA:ARBA00004922}.
protein ubiquitination. {ECO:0000256|ARBA:ARBA00004906}.
Term
Category
GO: biological process
GO: cellular component
GO: molecular function
Pathway

Figure. S14b

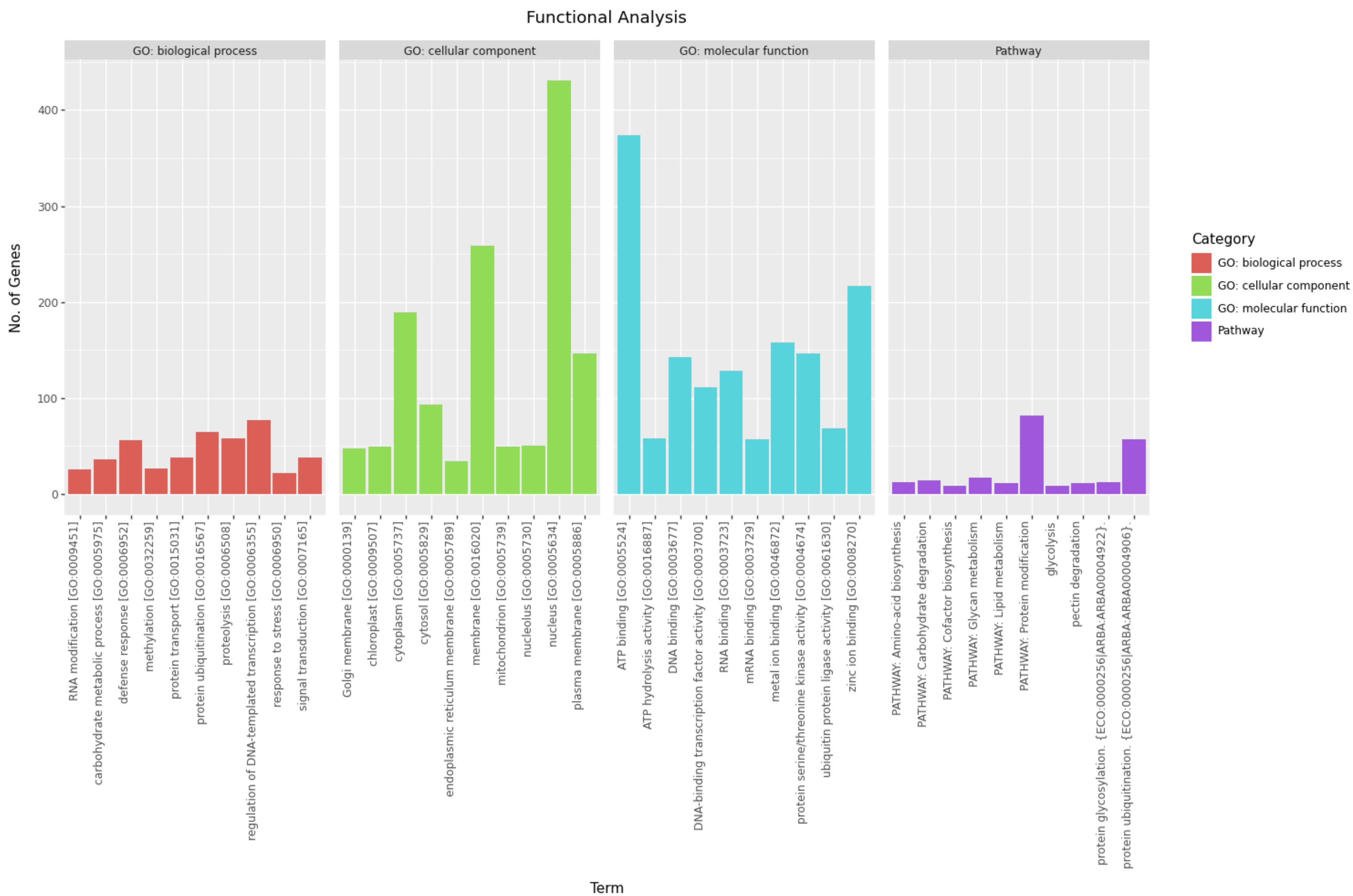
Functional Analysis
GO: biological process
GO: cellular component
GO: molecular function
Pathway
No. of Genes
0
100
200
300
400
RNA modification [GO:0009451]
carbohydrate metabolic process [GO:0005975]
defense response [GO:0006952]
methylation [GO:0032259]
protein transport [GO:0015031]
protein ubiquitination [GO:0016567]
proteolysis [GO:0006508]
regulation of DNA-templated transcription [GO:0006355]
response to stress [GO:0006950]
signal transduction [GO:0007165]
Golgi membrane [GO:0000139]
chloroplast [GO:0009507]
cytoplasm [GO:0005737]
cytosol [GO:0005829]
endoplasmic reticulum membrane [GO:0005789]
membrane [GO:0016020]
mitochondrion [GO:0005739]
nucleolus [GO:0005730]
nucleus [GO:0005634]
plasma membrane [GO:0005886]
ATP binding [GO:0005524]
ATP hydrolysis activity [GO:0016887]
DNA binding [GO:0003677]
DNA-binding transcription factor activity [GO:0003700]
RNA binding [GO:0003723]
mRNA binding [GO:0003729]
metal ion binding [GO:0046872]
protein serine/threonine kinase activity [GO:0004674]
ubiquitin protein ligase activity [GO:0061630]
zinc ion binding [GO:0008270]
PATHWAY: Amino-acid biosynthesis
PATHWAY: Carbohydrate degradation
PATHWAY: Cofactor biosynthesis
PATHWAY: Glycan metabolism
PATHWAY: Lipid metabolism
PATHWAY: Protein modification
glycolysis
pectin degradation
protein glycosylation. {ECO:0000256|ARBA:ARBA00004922}.
protein ubiquitination. {ECO:0000256|ARBA:ARBA00004906}.
Term
Category
GO: biological process
GO: cellular component
GO: molecular function
Pathway

Figure. S15a

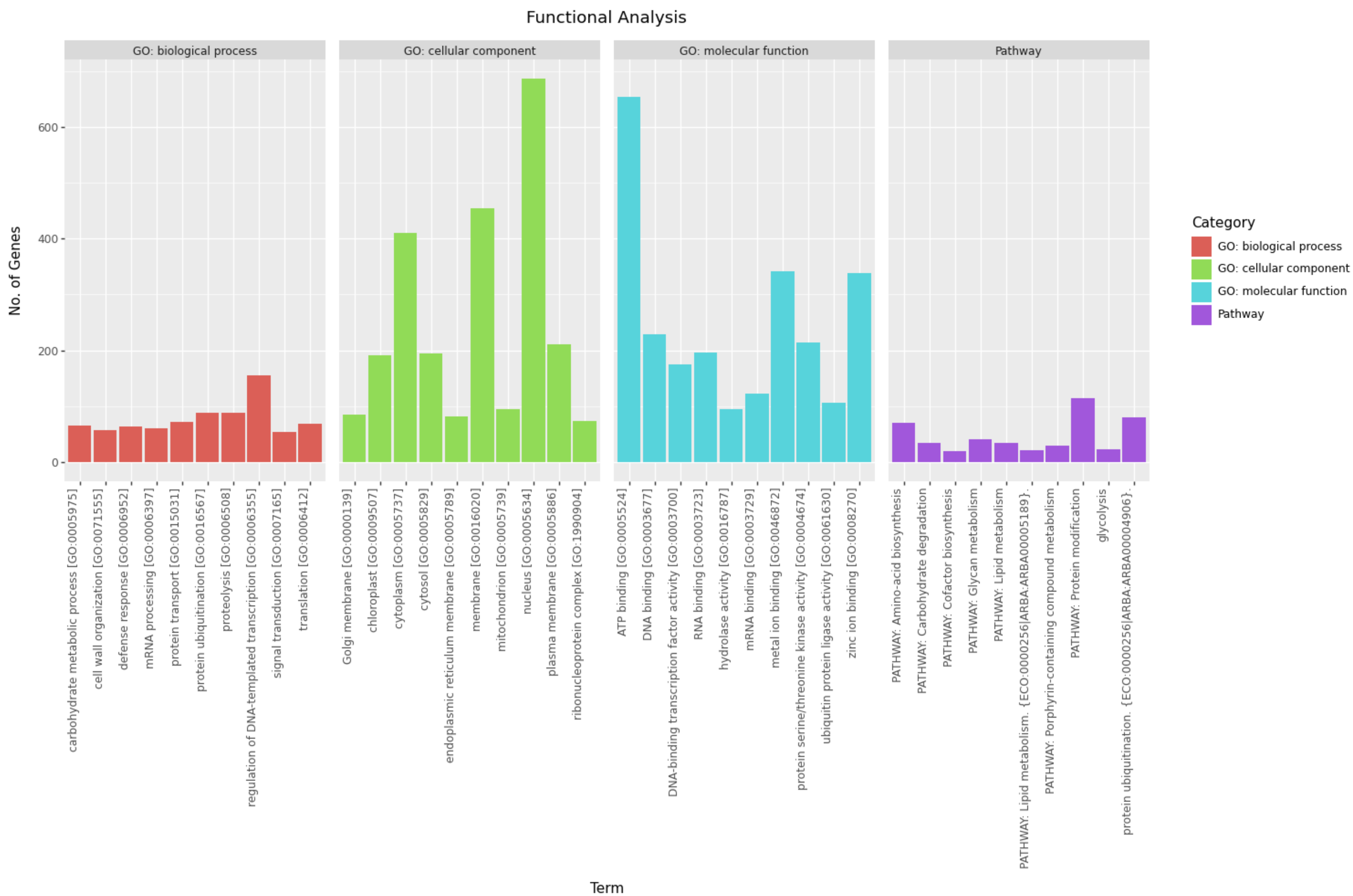

Functional Analysis
GO: biological process
GO: cellular component
GO: molecular function
Pathway
No. of Genes
600
400
200
0
carbohydrate metabolic process [GO:0005975]
cell wall organization [GO:0071555]
defense response [GO:0006952]
mRNA processing [GO:0006397]
protein transport [GO:0015031]
protein ubiquitination [GO:0016567]
proteolysis [GO:0006508]
regulation of DNA-templated transcription [GO:0006355]
signal transduction [GO:0007165]
translation [GO:0006412]
Golgi membrane [GO:0000139]
chloroplast [GO:0009507]
cytoplasm [GO:0005737]
cytosol [GO:0005829]
endoplasmic reticulum membrane [GO:0005789]
membrane [GO:0016020]
mitochondrion [GO:0005739]
nucleus [GO:0005634]
plasma membrane [GO:0005886]
ribonucleoprotein complex [GO:1990904]
ATP binding [GO:0005524]
DNA binding [GO:0003677]
DNA-binding transcription factor activity [GO:0003700]
RNA binding [GO:0003723]
hydrolase activity [GO:0016787]
mRNA binding [GO:0003729]
metal ion binding [GO:0046872]
protein serine/threonine kinase activity [GO:0004674]
ubiquitin protein ligase activity [GO:0061630]
zinc ion binding [GO:0008270]
PATHWAY: Amino-acid biosynthesis
PATHWAY: Carbohydrate degradation
PATHWAY: Cofactor biosynthesis
PATHWAY: Glycan metabolism
PATHWAY: Lipid metabolism
PATHWAY: Lipid metabolism. {ECO:0000256|ARBA:ARBA00005189}.
PATHWAY: Porphyrin-containing compound metabolism
PATHWAY: Protein modification
glycolysis
protein ubiquitination. {ECO:0000256|ARBA:ARBA00004906}.
Term
Category
GO: biological process
GO: cellular component
GO: molecular function
Pathway

Figure. S15b

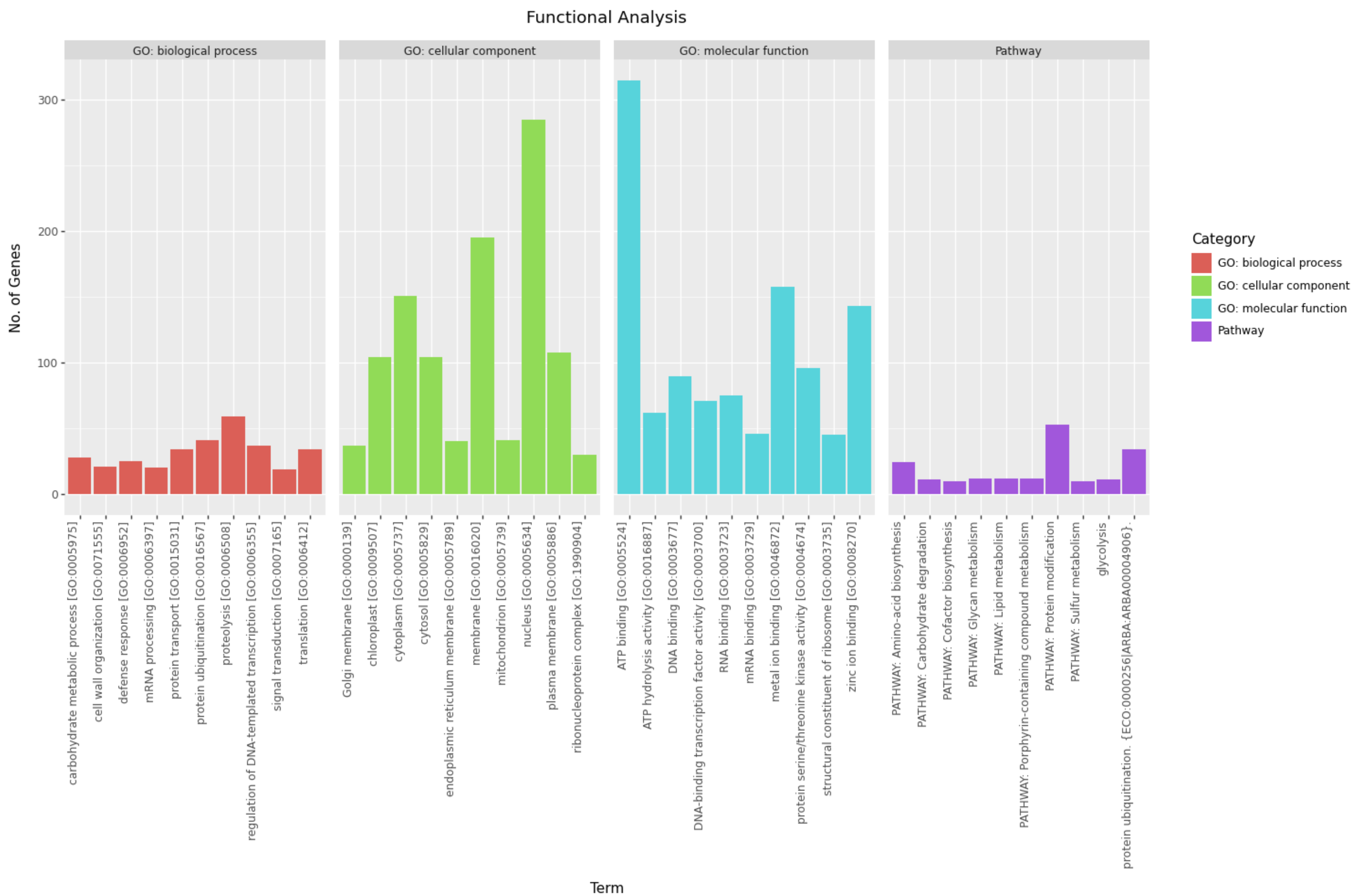
Functional Analysis
GO: biological process
GO: cellular component
GO: molecular function
Pathway
No. of Genes
0
100
200
300
carbohydrate metabolic process [GO:0005975]
cell wall organization [GO:0071555]
defense response [GO:0006952]
mRNA processing [GO:0006397]
protein transport [GO:0015031]
protein ubiquitination [GO:0016567]
proteolysis [GO:0006508]
regulation of DNA-templated transcription [GO:0006355]
signal transduction [GO:0007165]
translation [GO:0006412]
Golgi membrane [GO:0000139]
chloroplast [GO:0009507]
cytoplasm [GO:0005737]
cytosol [GO:0005829]
endoplasmic reticulum membrane [GO:0005789]
membrane [GO:0016020]
mitochondrion [GO:0005739]
nucleus [GO:0005634]
plasma membrane [GO:0005886]
ribonucleoprotein complex [GO:1990904]
ATP binding [GO:0005524]
ATP hydrolysis activity [GO:0016887]
DNA binding [GO:0003677]
DNA-binding transcription factor activity [GO:0003700]
RNA binding [GO:0003723]
mRNA binding [GO:0003729]
metal ion binding [GO:0046872]
protein serine/threonine kinase activity [GO:0004674]
structural constituent of ribosome [GO:0003735]
zinc ion binding [GO:0008270]
PATHWAY: Amino-acid biosynthesis
PATHWAY: Carbohydrate degradation
PATHWAY: Cofactor biosynthesis
PATHWAY: Glycan metabolism
PATHWAY: Lipid metabolism
PATHWAY: Porphyrin-containing compound metabolism
PATHWAY: Protein modification
PATHWAY: Sulfur metabolism
glycolysis
protein ubiquitination. {ECO:0000256|ARBA:ARBA00004906}.
Term
Category
GO: biological process
GO: cellular component
GO: molecular function
Pathway

Figure. S16a

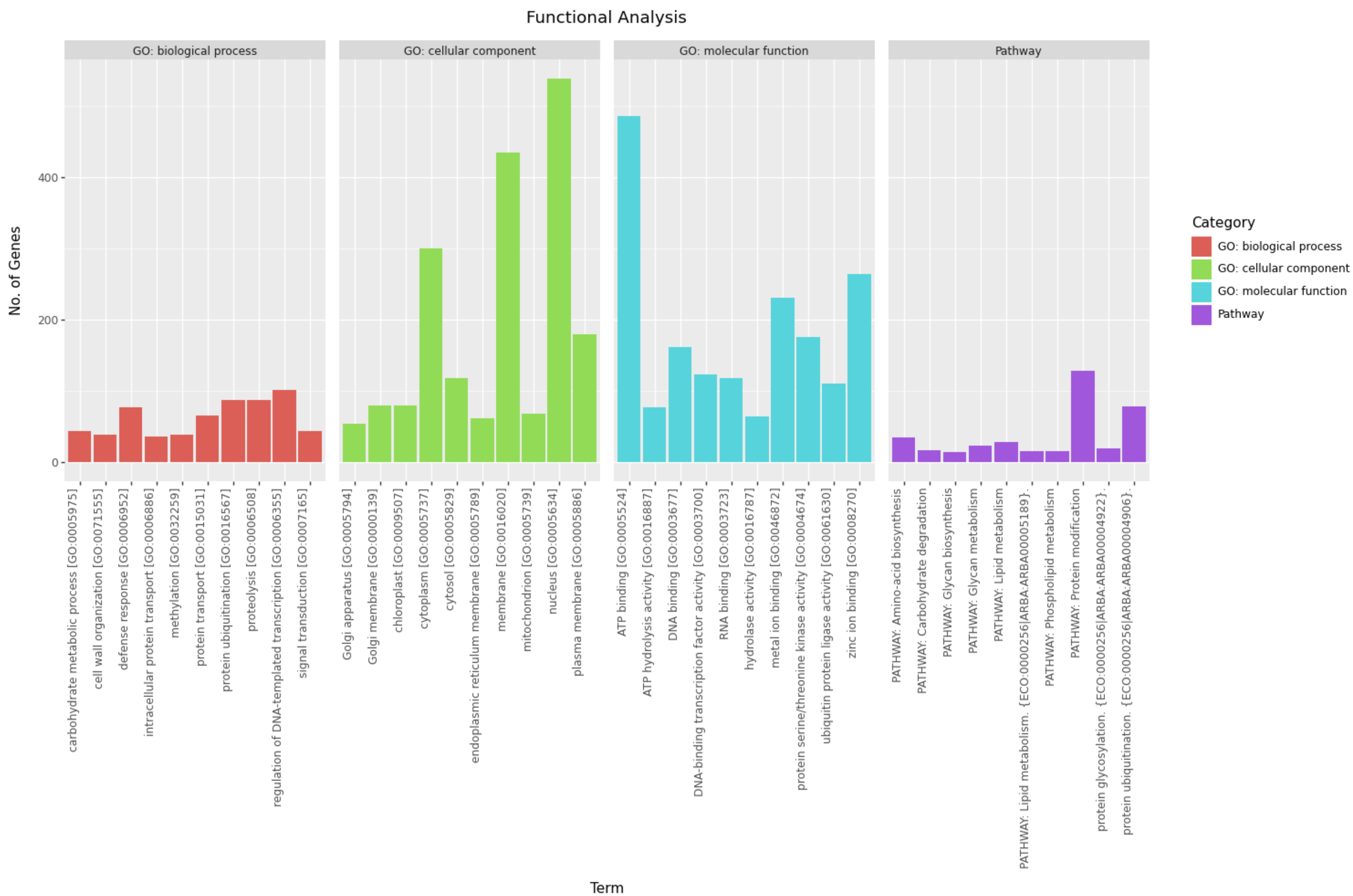
Functional Analysis
GO: biological process
GO: cellular component
GO: molecular function
Pathway
No. of Genes
0
200
400
carbohydrate metabolic process [GO:0005975]
cell wall organization [GO:0071555]
defense response [GO:0006952]
intracellular protein transport [GO:0006886]
methylation [GO:0032259]
protein transport [GO:0015031]
protein ubiquitination [GO:0016567]
proteolysis [GO:0006508]
regulation of DNA-templated transcription [GO:0006355]
signal transduction [GO:0007165]
Golgi apparatus [GO:0005794]
Golgi membrane [GO:0000139]
chloroplast [GO:0009507]
cytoplasm [GO:0005737]
cytosol [GO:0005829]
endoplasmic reticulum membrane [GO:0005789]
membrane [GO:0016020]
mitochondrion [GO:0005739]
nucleus [GO:0005634]
plasma membrane [GO:0005886]
ATP binding [GO:0005524]
ATP hydrolysis activity [GO:0016887]
DNA binding [GO:0003677]
DNA-binding transcription factor activity [GO:0003700]
RNA binding [GO:0003723]
hydrolase activity [GO:0016787]
metal ion binding [GO:0046872]
protein serine/threonine kinase activity [GO:0004674]
ubiquitin protein ligase activity [GO:0061630]
zinc ion binding [GO:0008270]
PATHWAY: Amino-acid biosynthesis
PATHWAY: Carbohydrate degradation
PATHWAY: Glycan biosynthesis
PATHWAY: Glycan metabolism
PATHWAY: Lipid metabolism
PATHWAY: Lipid metabolism. {ECO:0000256|ARBA:ARBA00005189}.
PATHWAY: Phospholipid metabolism
PATHWAY: Protein modification
protein glycosylation. {ECO:0000256|ARBA:ARBA00004922}.
protein ubiquitination. {ECO:0000256|ARBA:ARBA00004906}.
Term
Category
GO: biological process
GO: cellular component
GO: molecular function
Pathway

Figure. S16b

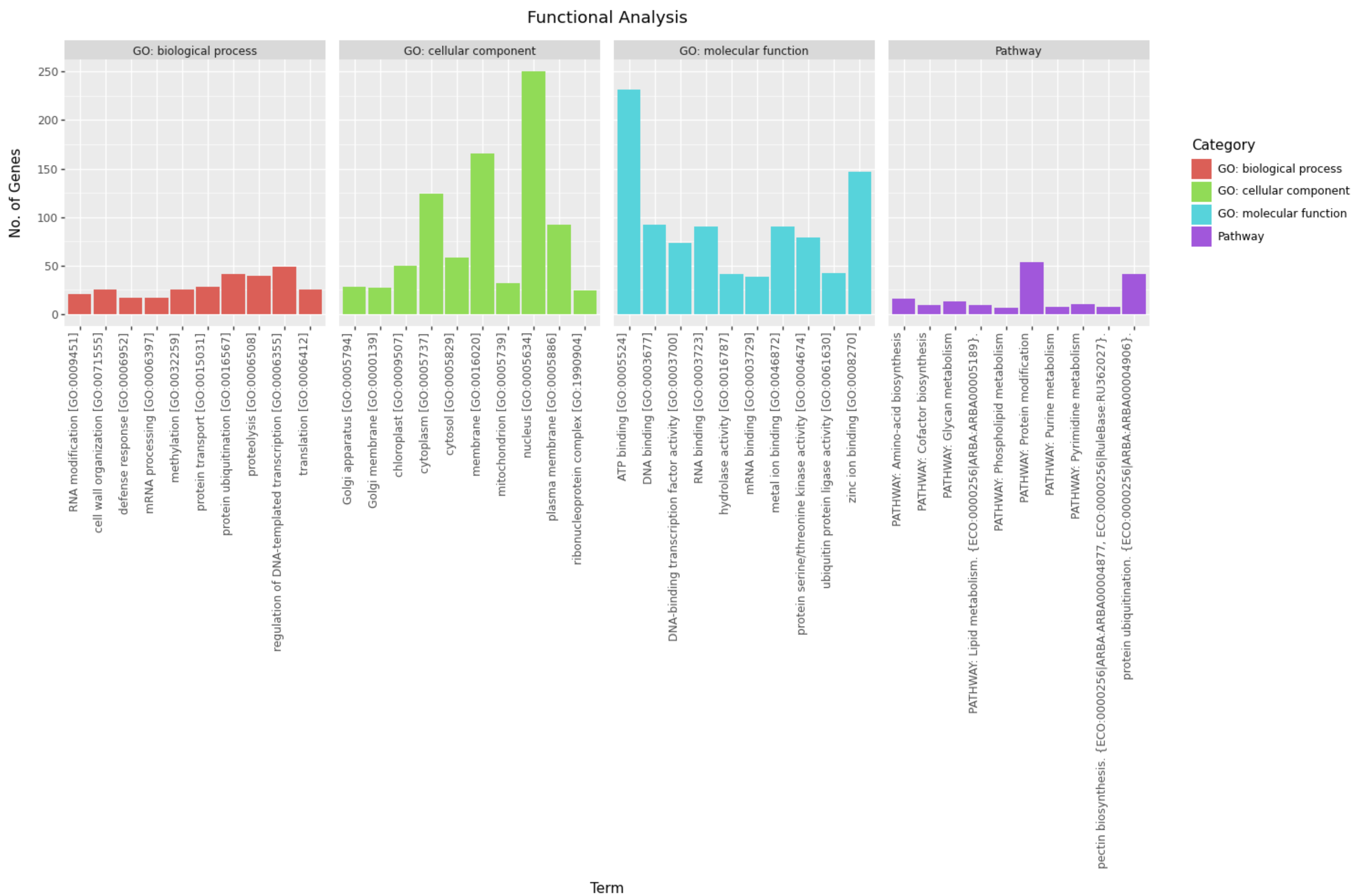
Functional Analysis
GO: biological process
GO: cellular component
GO: molecular function
Pathway
No. of Genes
250
200
150
100
50
0
RNA modification [GO:0009451]
cell wall organization [GO:0071555]
defense response [GO:0006952]
mRNA processing [GO:0006397]
methylation [GO:0032259]
protein transport [GO:0015031]
protein ubiquitination [GO:0016567]
proteolysis [GO:0006508]
regulation of DNA-templated transcription [GO:0006355]
translation [GO:0006412]
Golgi apparatus [GO:0005794]
Golgi membrane [GO:0000139]
chloroplast [GO:0009507]
cytoplasm [GO:0005737]
cytosol [GO:0005829]
membrane [GO:0016020]
mitochondrion [GO:0005739]
nucleus [GO:0005634]
plasma membrane [GO:0005886]
ribonucleoprotein complex [GO:1990904]
ATP binding [GO:0005524]
DNA binding [GO:0003677]
DNA-binding transcription factor activity [GO:0003700]
RNA binding [GO:0003723]
hydrolase activity [GO:0016787]
mRNA binding [GO:0003729]
metal ion binding [GO:0046872]
protein serine/threonine kinase activity [GO:0004674]
ubiquitin protein ligase activity [GO:0061630]
zinc ion binding [GO:0008270]
PATHWAY: Amino-acid biosynthesis
PATHWAY: Cofactor biosynthesis
PATHWAY: Glycan metabolism
PATHWAY: Lipid metabolism. {ECO:0000256|ARBA:ARBA00005189}.
PATHWAY: Phospholipid metabolism
PATHWAY: Protein modification
PATHWAY: Purine metabolism
PATHWAY: Pyrimidine metabolism
pectin biosynthesis. {ECO:0000256|ARBA:ARBA00004877, ECO:0000256|RuleBase:RU362027}.
protein ubiquitination. {ECO:0000256|ARBA:ARBA00004906}.
Term
Category
GO: biological process
GO: cellular component
GO: molecular function
Pathway

Figure. S17a

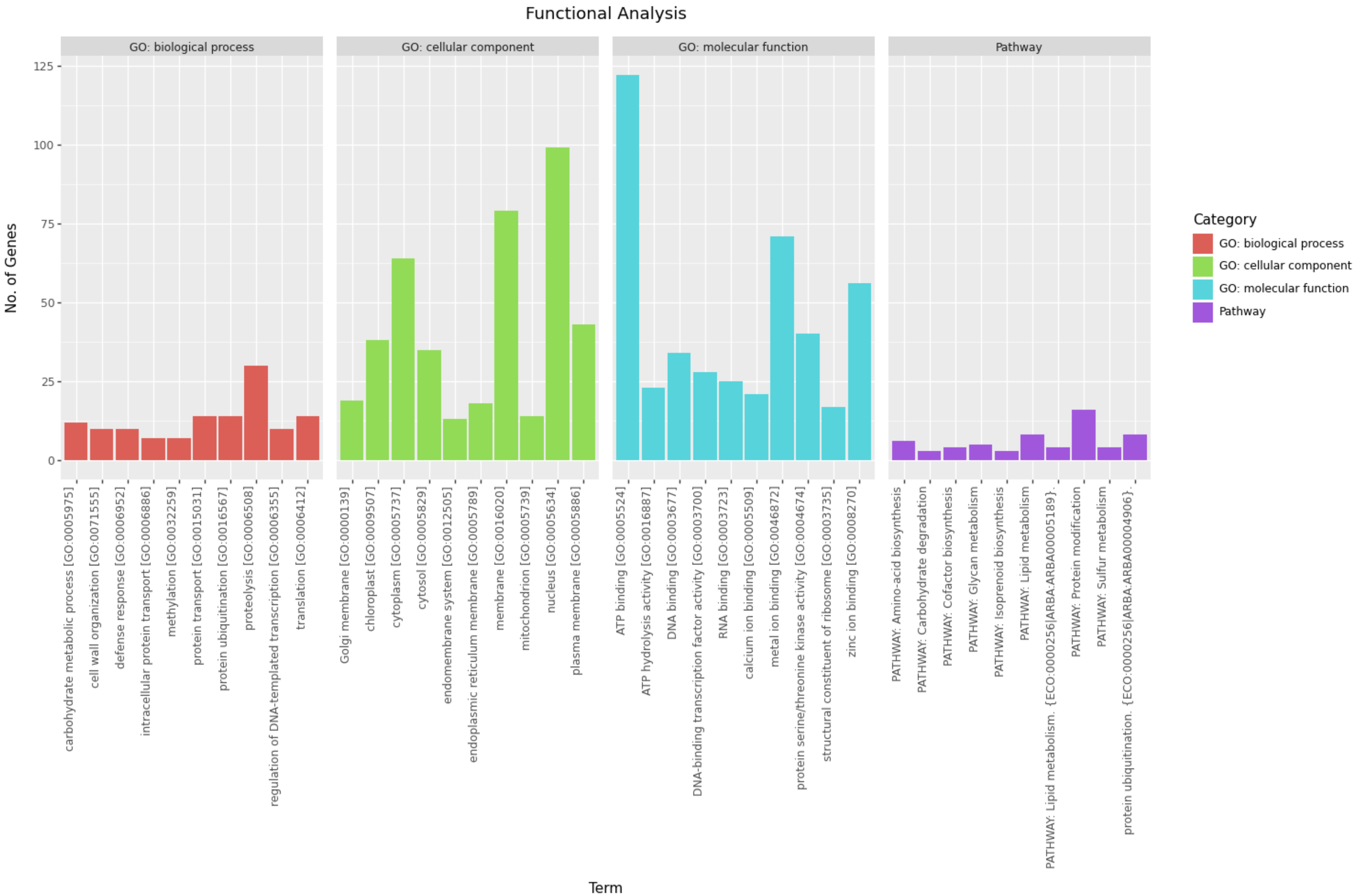

Functional Analysis
GO: biological process
GO: cellular component
GO: molecular function
Pathway
No. of Genes
125
100
75
50
25
0
carbohydrate metabolic process [GO:0005975]
cell wall organization [GO:0071555]
defense response [GO:0006952]
intracellular protein transport [GO:0006886]
methylation [GO:0032259]
protein transport [GO:0015031]
protein ubiquitination [GO:0016567]
proteolysis [GO:0006508]
regulation of DNA-templated transcription [GO:0006355]
translation [GO:0006412]
Golgi membrane [GO:0000139]
chloroplast [GO:0009507]
cytoplasm [GO:0005737]
cytosol [GO:0005829]
endomembrane system [GO:0012505]
endoplasmic reticulum membrane [GO:0005789]
membrane [GO:0016020]
mitochondrion [GO:0005739]
nucleus [GO:0005634]
plasma membrane [GO:0005886]
ATP binding [GO:0005524]
ATP hydrolysis activity [GO:0016887]
DNA binding [GO:0003677]
DNA-binding transcription factor activity [GO:0003700]
RNA binding [GO:0003723]
calcium ion binding [GO:0005509]
metal ion binding [GO:0046872]
protein serine/threonine kinase activity [GO:0004674]
structural constituent of ribosome [GO:0003735]
zinc ion binding [GO:0008270]
PATHWAY: Amino-acid biosynthesis
PATHWAY: Carbohydrate degradation
PATHWAY: Cofactor biosynthesis
PATHWAY: Glycan metabolism
PATHWAY: Isoprenoid biosynthesis
PATHWAY: Lipid metabolism
PATHWAY: Lipid metabolism. {ECO:0000256|ARBA:ARBA00005189}.
PATHWAY: Protein modification
PATHWAY: Sulfur metabolism
protein ubiquitination. {ECO:0000256|ARBA:ARBA00004906}.
Term
Category
GO: biological process
GO: cellular component
GO: molecular function
Pathway

Figure. S18a

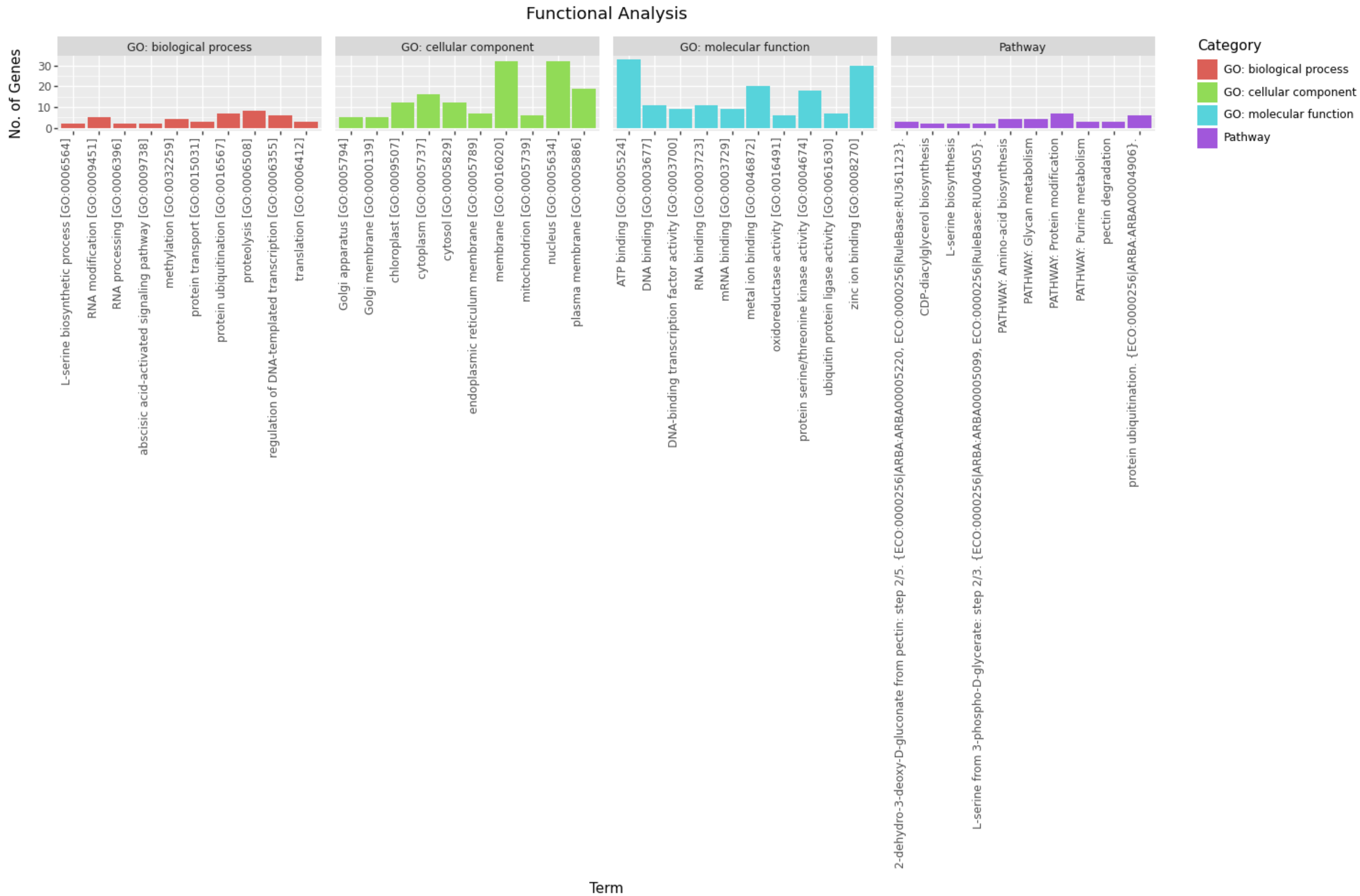
Functional Analysis
GO: biological process
GO: cellular component
GO: molecular function
Pathway
No. of Genes
30
20
10
0
L-serine biosynthetic process [GO:0006564]
RNA modification [GO:0009451]
RNA processing [GO:0006396]
abscisic acid-activated signaling pathway [GO:0009738]
methylation [GO:0032259]
protein transport [GO:0015031]
protein ubiquitination [GO:0016567]
proteolysis [GO:0006508]
regulation of DNA-templated transcription [GO:0006355]
translation [GO:0006412]
Golgi apparatus [GO:0005794]
Golgi membrane [GO:0000139]
chloroplast [GO:0009507]
cytoplasm [GO:0005737]
cytosol [GO:0005829]
endoplasmic reticulum membrane [GO:0005789]
membrane [GO:0016020]
mitochondrion [GO:0005739]
nucleus [GO:0005634]
plasma membrane [GO:0005886]
ATP binding [GO:0005524]
DNA binding [GO:0003677]
DNA-binding transcription factor activity [GO:0003700]
RNA binding [GO:0003723]
mRNA binding [GO:0003729]
metal ion binding [GO:0046872]
oxidoreductase activity [GO:0016491]
protein serine/threonine kinase activity [GO:0004674]
ubiquitin protein ligase activity [GO:0061630]
zinc ion binding [GO:0008270]
2-dehydro-3-deoxy-D-gluconate from pectin: step 2/5. {ECO:0000256|ARBA:ARBA00005220, ECO:0000256|RuleBase:RU361123}.
CDP-diacylglycerol biosynthesis
L-serine biosynthesis
L-serine from 3-phospho-D-glycerate: step 2/3. {ECO:0000256|ARBA:ARBA00005099, ECO:0000256|RuleBase:RU004505}.
PATHWAY: Amino-acid biosynthesis
PATHWAY: Glycan metabolism
PATHWAY: Protein modification
PATHWAY: Purine metabolism
pectin degradation
protein ubiquitination. {ECO:0000256|ARBA:ARBA00004906}.
Term
Category
GO: biological process
GO: cellular component
GO: molecular function
Pathway

Figure. S18b

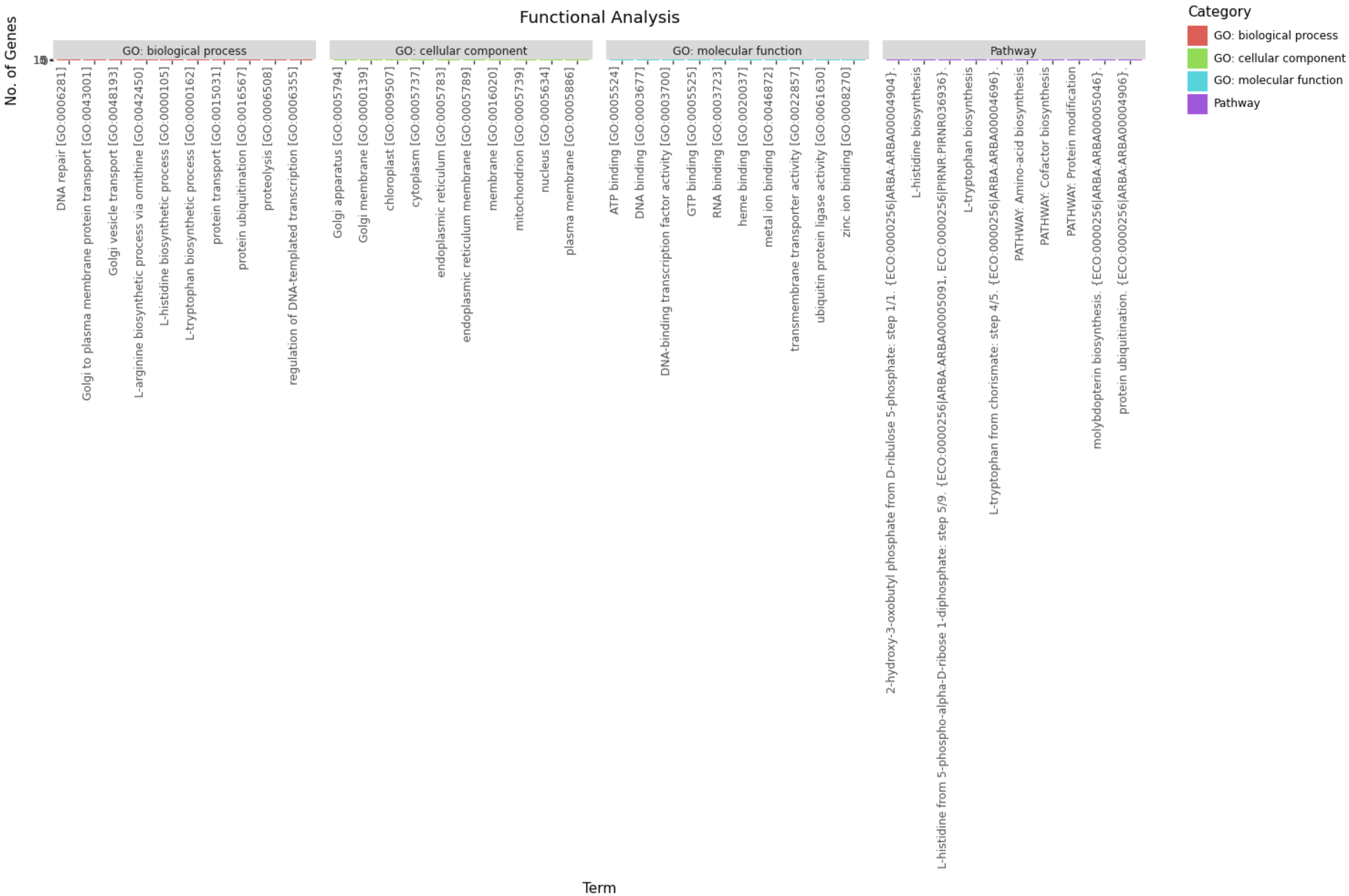
Functional Analysis
No. of Genes
10
5
GO: biological process
GO: cellular component
GO: molecular function
Pathway
DNA repair [GO:0006281]
Golgi to plasma membrane protein transport [GO:0043001]
Golgi vesicle transport [GO:0048193]
L-arginine biosynthetic process via ornithine [GO:0042450]
L-histidine biosynthetic process [GO:0000105]
L-tryptophan biosynthetic process [GO:0000162]
protein transport [GO:0015031]
protein ubiquitination [GO:0016567]
proteolysis [GO:0006508]
regulation of DNA-templated transcription [GO:0006355]
Golgi apparatus [GO:0005794]
Golgi membrane [GO:0000139]
chloroplast [GO:0009507]
cytoplasm [GO:0005737]
endoplasmic reticulum [GO:0005783]
endoplasmic reticulum membrane [GO:0005789]
membrane [GO:0016020]
mitochondrion [GO:0005739]
nucleus [GO:0005634]
plasma membrane [GO:0005886]
ATP binding [GO:0005524]
DNA binding [GO:0003677]
DNA-binding transcription factor activity [GO:0003700]
GTP binding [GO:0005525]
RNA binding [GO:0003723]
heme binding [GO:0020037]
metal ion binding [GO:0046872]
transmembrane transporter activity [GO:0022857]
ubiquitin protein ligase activity [GO:0061630]
zinc ion binding [GO:0008270]
2-hydroxy-3-oxobutyl phosphate from D-ribulose 5-phosphate: step 1/1. {ECO:0000256|ARBA:ARBA00004904}.
L-histidine biosynthesis
L-histidine from 5-phospho-alpha-D-ribose 1-diphosphate: step 5/9. {ECO:0000256|ARBA:ARBA00005091, ECO:0000256|PIRNR:PIRNR036936}.
L-tryptophan biosynthesis
L-tryptophan from chorismate: step 4/5. {ECO:0000256|ARBA:ARBA00004696}.
PATHWAY: Amino-acid biosynthesis
PATHWAY: Cofactor biosynthesis
PATHWAY: Protein modification
molybdopterin biosynthesis. {ECO:0000256|ARBA:ARBA00005046}.
protein ubiquitination. {ECO:0000256|ARBA:ARBA00004906}.
Term
Category
GO: biological process
GO: cellular component
GO: molecular function
Pathway
